# Conjugating effects of symbionts and environmental factors on gene expression in deep-sea hydrothermal vent mussels

**DOI:** 10.1186/1471-2164-12-530

**Published:** 2011-10-28

**Authors:** Isabelle Boutet, Raymond Ripp, Odile Lecompte, Carole Dossat, Erwan Corre, Arnaud Tanguy, François H Lallier

**Affiliations:** 1 CNRS, UMR 7144, Adaptation et Diversité en Milieu Marin, Station Biologique de Roscoff, 29682 Roscoff, France; 2 UPMC Université Paris 06, Station Biologique de Roscoff, 29682 Roscoff, France; 3 Department of Structural Biology and Genomics, Institut de Génétique et de Biologie Moléculaire et Cellulaire (IGBMC), INSERM U596, UMR CNRS 7104, Faculté des Sciences de la Vie, Université de Strasbourg, 67000 Strasbourg, France; 4 Génoscope, Centre National de Séquençage, UMR CNRS 8030, 2 Gaston Crémieux, CP 5706, 91507 Evry, France; 5 Service Informatique et Génomique FR2424, Analysis and Bioinformatics for Marine Science Platform (ABiMS), Station Biologique de Roscoff, 29682 Roscoff, France

**Keywords:** *Bathymodiolus azoricus*, symbiosis, gene expression, environment

## Abstract

**Background:**

The deep-sea hydrothermal vent mussel *Bathymodiolus azoricus *harbors thiotrophic and methanotrophic symbiotic bacteria in its gills. While the symbiotic relationship between *this hydrothermal mussel and *these chemoautotrophic bacteria has been described, the molecular processes involved in the cross-talking between symbionts and host, in the maintenance of the symbiois, in the influence of environmental parameters on gene expression, and in transcriptome variation across individuals remain poorly understood. In an attempt to understand how, and to what extent, this double symbiosis affects host gene expression, we used a transcriptomic approach to identify genes potentially regulated by symbiont characteristics, environmental conditions or both. This study was done on mussels from two contrasting populations.

**Results:**

Subtractive libraries allowed the identification of about 1000 genes putatively regulated by symbiosis and/or environmental factors. Microarray analysis showed that 120 genes (3.5% of all genes) were differentially expressed between the Menez Gwen (MG) and Rainbow (Rb) vent fields. The total number of regulated genes in mussels harboring a high versus a low symbiont content did not differ significantly. With regard to the impact of symbiont content, only 1% of all genes were regulated by thiotrophic (SOX) and methanotrophic (MOX) bacteria content in MG mussels whereas 5.6% were regulated in mussels collected at Rb. MOX symbionts also impacted a higher proportion of genes than SOX in both vent fields. When host transcriptome expression was analyzed with respect to symbiont gene expression, it was related to symbiont quantity in each field.

**Conclusions:**

Our study has produced a preliminary description of a transcriptomic response in a hydrothermal vent mussel host of both thiotrophic and methanotrophic symbiotic bacteria. This model can help to identify genes involved in the maintenance of symbiosis or regulated by environmental parameters. Our results provide evidence of symbiont effect on transcriptome regulation, with differences related to type of symbiont, even though the relative percentage of genes involved remains limited. Differences observed between the vent site indicate that environment strongly influences transcriptome regulation and impacts both activity and relative abundance of each symbiont. Among all these genes, those participating in recognition, the immune system, oxidative stress, and energy metabolism constitute new promising targets for extended studies on symbiosis and the effect of environmental parameters on the symbiotic relationships in *B. azoricus*.

## Background

Symbiosis, defined as an interdependent relationship between two species, is an important driver of evolution, diversity, and increased plasticity in eukaryotes. The underlying biological processes of these associations were highlighted by recent analyses coupling genomic and evolutionary data [1] that showed that a part of biological adaptation and phenotypic novelty in a species is due to the acquisition of functional systems from other species in a mutualistic symbiosis. In the ultimate mutualistic association, the symbionts are located in host cells and are transmitted vertically through successive generations. This kind of association was at the root of mitochondria and chloroplast establishment in eukaryotes [2]. Similarly, associations between chemoautotrophic bacteria (thiotrophic and/or methanotrophic) and invertebrates are ubiquitously described in reducing marine ecosystems, such as mangrove mud, anoxic sediments, hydrothermal vents and cold seeps [3]. The symbiotic relationship between chemoautotrophic bacteria and invertebrates at deep-sea hydrothermal vents and cold seeps, as well as the parameters influencing the regulation and variations of mRNA expression across individuals, remains poorly understood at the transcriptome level, even though symbiotic organisms are the major component of biomass in these ecosystems. Different studies have focused on the process of symbiont acquisition and characterization of genes differentially regulated between organisms at different symbiotic states [4-10]. These transcriptomic approaches are based on sequence libraries and expression levels determined by real-time PCR that are mainly descriptive and therefore do not give information about the source of inter-individual gene expression variations. For example, are these variations due to the symbionts and/or environmental conditions? Moreover, these studies were mainly conducted on organisms under laboratory conditions to identify regulated genes and analyze their mRNA expression during the process of symbiont acquisition. In their work, Voolstra et al. [9] followed gene expression of larvae of the corals *Acropora palmate *and *Montastraea faveolata *after exposure to *Symbiodinium *algal strains that differed in their ability to establish symbiosis. They showed that the corals' transcriptomes remained almost unchanged during infection by competent symbionts, but were altered by symbionts that failed to establish symbiosis. The authors suggested that successful coral-algal symbiosis depended mainly on the symbionts' ability to enter the host in a stealth manner rather than by provoking a more active response from the coral host. Environmental factors, such as water temperature, had a major impact on the symbiotic relationship between coral and zooxanthellae by compromising the acceptance of the symbiont by the host during the acquisition step, and consequently, variations in gene expression of the host were observed [10]. These studies on symbiotic marine organisms provide evidence of the combined impact of symbionts and environmental factors on the mRNA expression of the host.

Hydrothermal vent mussels of the genus *Bathymodiolus *are distributed worldwide and often constitute a major component of the fauna inhabiting hydrothermal vents and cold seeps. The majority of hydrothermal and cold-seep organisms developed a single endosymbiosis, generally with sulfur-oxidizing (SOX) bacteria, though occasionally with methanotrophs (MOX). Vent and seep mussels harbor either a single endosymbiont strain, like *B. thermophilus *(SOX bacteria) or *B. childressi *(MOX bacteria) [11,12], or possess a double symbioses with both SOX and MOX bacteria, such as *B. brooksii*, *B. heckerae*, *B. azoricus *and *B. puteoserpentis *[13-16]. In contrast to gutless chemosymbiotic organisms in which the relationship with bacteria is obligatory, these mussels possess a functional digestive tract and isotope analyses have shown that they are able to obtain food by suspension feeding when necessary [17-19]. Moreover, Fisher and Childress [20] used both stable isotopes and histology to demonstrate that nutrient transfer from symbionts to mussel tissues results from the digestion of symbionts rather than nutrient translocation. The phylogeny of symbionts, especially those of both vent and seep mussels, has been thoroughly studied [15,21,22], as well as their (co-) localization in gill filaments [15,21-23]. Large genome- and transcriptome-scale analyses of hydrothermal vent host organisms have so far been done on symbiosis in tubeworms [4,6] and heat adaptation [24-26]. No such studies have been done on symbiosis in tubeworms [4,6] and heat adaptation [24-26]. No such studies have been done on *Bathymodiolus *symbiosis, despite its importance in hydrothermal vent ecosystems.

In the context of investigating chemoautotrophic symbiosis in vent taxa, we focused on the effect of both environmental factors and symbiont content on the established double symbiosis of vent mussels at the transcriptome level. Our approach combined the analysis of microarrays comprised of differentially expressed genes determined through suppressive subtraction hybridization (SSH) between hydrothermal vent mussels of *B. azoricus *inhabiting two different vent fields of the Mid-Atlantic Ridge (MAR) - different in depth, fluid temperature, pH, and metal and methane concentrations. Our objective was to determine the effects of symbiont quantity and type, as well as environmental factors, on host gene expression at the transcriptome level in order to identify clusters of genes involved in the maintenance of symbiosis and/or in the response to environmental variations.

## Results

### Quantification of symbiont-specific gene expression

**Figure 1:**
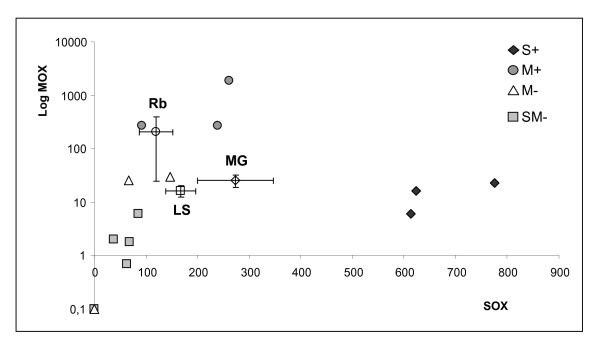
**Quantification of symbionts in the mussels *B. azoricus *collected at the three vent fields Menez Gwen (MG, *n *= 25), Lucky Strike (LS, *n *= 30) and Rainbow (Rb, *n *= 25)**. Symbiont quantities in mussels used in subtractive library design are presented individually. S+, high SOX content (*n *= 3 ind from MG); SM-, low SOX and low MOX content (*n *= 5 ind from MG, LS and Rb); M-, low MOX content (*n *= 3 ind from Rb); M+, high MOX content (*n *= 3 ind from Rb). Quantities of SOX and MOX are given as relative quantity in an arbitrary unit.

Real-time PCR measured significant differences (*P *< 0.05) of both MOX and SOX quantities, and of symbiont-specific gene expression of ATP sulfurylase and monooxygenase A (pmoA), between the three vent fields: Menez Gwen (MG), Lucky Strike (LS) and Rainbow (Rb) (Table 1, Figure 1). Mussels collected at MG had a higher SOX content (273) and ATP sulfurylase expression (1.1 × 10^-5^) compared to mussels collected at LS (167 and 8 × 10^-6^, respectively) and at Rb (119 and 4.5 × 10^-6^, respectively). Conversely, higher MOX content and pmoA expression were recorded in mussels collected at Rb (209 and 0.29, respectively) compared to mussels from MG (26 and 0.12, respectively) and LS (16 and 0.07, respectively).

**Table 1:** Main concentrations in the Menez Gwen, Lucky Strike and Rainbow vent fluids, adapted from Douville *et al*. (2002), Charlou *et al*. (2000, 2002).

	Menez Gwen (37°50'N)	Lucky Strike (37°17'N)	Rainbow (36°14'N)
***Physical and chemical characteristics***			
**Depth **(m)	850	1700	2300
**Temperature **(°C)	265-284	152-333	360-365
**pH**	4.2-4.8	3.5-4.9	2.8-3.1
**Fe **(mg l^-1^)	1.3-1.6	1.7-48	1339
**Mn **(mg l^-1^)	3.2-3.7	4.2-24.7	123
**Cu **(mg l^-1^)	40-180	60-1650	8900
**Zn **(mg l^-1^)	0.16-0.33	0.33-3.79	10.5
**Cd **(mg l^-1^)	1.01-1.34	2.02-8.85	14.6
**Pb **(mg l^-1^)	4.4-11.6	7.2-26.9	30.6
**H_2_S **(mM)	1.3-1.82	0.6-3.3	1-2.52
**CH_4 _**(mM)	1.7	0.52	2.5

***Symbiont quantification***			
***n***	25	30	25
**SOX**	272.95 ± 73.45	166.71 ± 28.92	119.35 ± 32.05
**MOX**	25.70 ± 6.70	16.38 ± 4.07	209.19 ± 184.40
**ATP sulfurylase**	1.1 10^-5 ^± 4.8 10^-6^	8 10^-6 ^± 3 10^-6^	4.6 10^-6 ^± 1.3 10^-6^
**pmoA**	0.12 ± 0.04	0.07 ± 0.02	0.29 ± 0.09

Relative quantities and activity of sulfide oxidizer bacteria (SOX and ATP sulfurylase) and methanotrophic bacteria (MOX and particulate methane monooxygenase A pmoA) were characterized in the present study and given in an arbitrary unit.

### Sequencing of subtractive libraries

**Figure 2:**
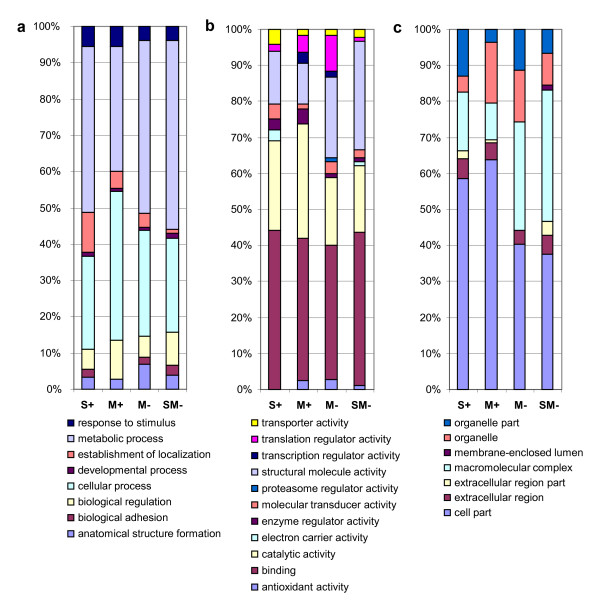
**Repartition of *B. azoricus *annotated proteins into the GO categories**. (a) Biological process, (b) Molecular function, (c) Cellular component. S+, high SOX content; M+, high MOX content; M-, low MOX content; SM-, low SOX and MOX content.

The sequencing of the SSH libraries allowed the identification of 1058 unique, expressed genes distributed as follows (arrow points to subtracted library, and +/- denotes relative level of S(OX) or M(OX)): S+ → M+: 90; M+ → S+: 139; S+ → SM-: 82; SM- → S+: 126; S+ → M-: 103; M- → S+: 110; M+ → M-: 108; M- → M+: 134; M+ → SM-: 50; SM- → M+: 116. The distribution of annotated proteins into the GO classes is shown in Figure 2. The sequences and their annotations are available online in a dedicated database using the following link: http://lbgi.igbmc.fr/Bathymodiolus/(Accession number: Genbank dbEST JK480449-JK483708).

### Microarray data analysis

We focused our analysis on MG and Rb vent fields, the two most contrasted in terms of chemical environment and relative abundance of SOX/MOX, to highlight the genes potentially regulated by symbionts and/or environmental parameters.

#### Gene expression according to vent site characteristics

**Figure 3:**
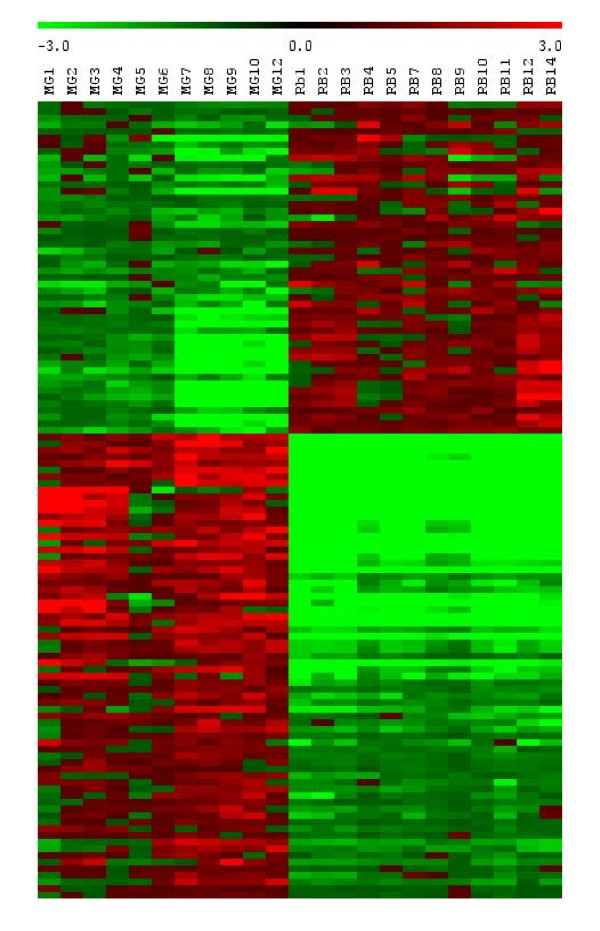
**Gene expression differentially regulated between Menez Gwen and Rainbow vent fields according to SAM results obtained with TmeV**.

To assess the influence of environmental parameters, the ratio of expression of each gene was calculated by dividing signal intensity of the gene for one given sample by the mean intensity of the gene in all samples in both populations. Our analyses showed that 120 genes (3.5% of all genes) are differentially expressed between MG and Rb: 50 genes had higher expression at Rb than at MG and 70 genes had higher expression at MG than at Rb (Figure 3, list available in Additional File 1: Tables 1, 2).

**Table 2:** Number of genes (and corresponding percentage to total number of genes) regulated by symbiont content within each vent field, commonly regulated by the two types of symbiont within vent field and by each type of symbiont across vent field.

	Menez Gwen		Rainbow		Regulated in the 2 vent fields	
			
Low SOX	128		286			
		*8%*		*16%*	36	*1.1%*
High SOX	133		259			
**Low MOX**	180		359			
		*14%*		*20%*	102	*3%*
**High MOX**	285		311			

Regulated by SOX and MOX						
	35	*1%*	192	*5.6%*	2	*0.1%*

#### Gene expression according to quantity and type of symbiont at a vent field scale

In order to identify genes potentially regulated by symbiont content and their respective activity, we conducted eight distinct analyses by classifying individuals according to their MOX and SOX content and ATP sulfurylase and pmoA expression levels for each population separately. For this study, the ratio of expression of each gene was calculated by dividing signal intensity of the gene for one given sample by the mean intensity of the gene in all samples from the same population. Our analyses indicated that symbiont quantity did not significantly affect the number of regulated genes (Table 2). One exception was observed for MOX quantity at MG, where a high MOX content regulated more genes than in mussels with a low MOX content (285 *vs*. 180, Table 2). We further analyzed the genes commonly regulated by both symbionts within each site. At the MG vent field, only 1% of all genes were regulated by SOX and MOX (Table 3), while 5.6% were regulated in mussels collected at Rb (Table 2). Interestingly, MOX symbiont impacted a higher proportion of genes than SOX in both vent fields (14-20% *vs*. 8-16%, Table 2), as well as genes commonly regulated by each symbiont in the two vent fields (1.1% *vs*. 3% for SOX and MOX, respectively, Table 2). A list of regulated genes in all conditions is available in Additional File 1: Tables 3-14. Regarding symbiont gene expression, we obtained a result in accordance with symbiont quantity at each site: ATP sulfurylase expression is higher at MG where SOX are abundant and pmoA expression is higher at Rb where MOX are abundant (Table 1, Additional File 1: Tables 15-26).

**Table 3:** Number of genes (and corresponding percentage to total number of genes) regulated by symbiont activity within each vent field, and commonly regulated by both symbiont quantity and its corresponding activity.

	Menez Gwen		Rainbow	
		
Low ATP sulfurylase	97		91	
		*9%*		*6%*
High ATP sulfurylase	195		114	
Regulated by SOX and ATP sulfurylase	17	*0.5%*	17	*0.5%*
		
**Low pmoA**	28		180	
		*6%*		*10%*
**High pmoA**	172		154	

Regulated by MOX and pmoA	86	*2.5%*	96	*2.8%*

## Discussion

Deciphering effects of symbionts and environmental factors on gene expression in field populations of symbiotic organisms is an important goal in molecular ecology. In the case of the vent mussel *B. azoricus*, genes regulated and/or involved in its symbiotic relationship with two symbionts were unknown. To address this issue, we used a combination of subtractive libraries and a cDNA microarray approach to characterize putative differentially expressed genes in the hydrothermal vent mussel *B. azoricus *inhabiting two physically and chemically contrasting vent fields. Then, we determined if the expression of these differentially expressed genes was, in part, influenced by symbiont content and/or symbiont metabolism, and/or environmental factors. Because we used mussels collected in their natural environment, we did not expect to identify genes involved in the establishment of symbiosis, but rather genes involved in an established symbiosis that were differentially regulated with respect to symbiont type, quantity and activity, and environmental factors.

### Vent field environmental conditions impact activity and relative abundance of both symbionts as well as host gene expression

The quantification of symbiotic bacteria in mussel gills showed a significant difference of both SOX and MOX content across the two vent fields. The relative abundance of the two symbionts is influenced by the hydrothermal fluid characteristics, especially methane and sulfide concentration. This correlation explains, for example, the higher abundance of MOX in mussels collected at Rb vent field where high concentrations of methane were detected [27,28], compared to individuals from the MG vent field [14,15,23]. The average quantity of each symbiont was significantly different between the two vent fields, but we observed a large inter-individual variation in symbiont abundance within each field. This symbiotic plasticity allows mussels from one vent field to harbor the same amount and/or proportion of symbionts as mussels from another field. Mussels from each vent field were collected in a restricted area of the mussel bed, but given the highly chaotic mixing conditions encountered at hydrothermal vents, we have to consider variations in both sulfide and methane availability, even at the scale of the sampling patch. These variations in gas availability in vent fields impact the distribution and diet of vent fauna at a micro-spatial scale [19] and may explain these differences in symbiont content.

The symbiotic vent mussel *B. azoricus *inhabits a variable environment due to the highly chaotic mixing of hydrothermal fluid with seawater within the site [29,30], and because of the bathymetric position of these hydrothermal fields [31]. Thus, a strong effect for environmental factors was expected due to the very different characteristics of the vent fluid in the two populations sampled (especially gas and heavy metals concentrations, temperature and pressure), and therefore specific signatures of the mRNA expression in mussels were expected for the different vent fields. Our microarray data showed that 120 genes (3.5% of all genes) clearly distinguish both sites, indicating a relatively moderate effect of source vent on transcriptome regulation. However, cluster analysis of all individuals showed a clear separation between samples from MG and Rb, indicating that the physical characteristics of the two vent fields were strong enough to influence transcriptome expression in a way that distinguishes populations.

Among the up-regulated genes in mussels collected at the Rainbow vent field, we identified a 60S acidic ribosomal protein and a selenoprotein. The 60S acidic ribosomal protein, known as a P protein, is mainly associated with the protein elongation step of translation, but potential roles in transcription, DNA repair [32], in response to pesticide exposure [33], and in intracellular iron sequestration [34] have also been described. The selenoprotein has multiple functions such as antioxidant defense, selenium transport and heavy metal chelation [35]. Up-regulation of these genes in mussels from the Rb vent field is consistent with the high metal concentrations at this site, the highest observed in the MAR hydrothermal area [27,36].

Among the genes significantly up-regulated in mussels collected at the MG vent field, we identified some metabolic genes such as arginine kinase and carbonic anhydrase (CA). Arginine kinase is known to play a key role in cellular energy metabolism in invertebrates [37] and its regulation in response to temperature has been previously described in *B. azoricus *[25]. Carbonic anhydrase is known to be involved in the transfer of CO_2 _from the environment to the cell in many symbiotic animals. This enzyme catalyzes the reversible hydration of CO_2_, and was found to be regulated at the transcriptome level according to the state of symbiosis, in both plants and animals [38], but also in *B. azoricus *in response to temperature variations [25].

Regarding the limited number of known genes and the lack of experimental studies performed on *B. azoricus *in response to various stressors, as well as the lack of information about the micro-environmental characteristics around mussels (especially for concentrations of H_2_S and CH_4_), it remains difficult to link these significantly regulated genes to environmental parameters. Moreover, our sampling strategy of collecting mussels directly from their environment did not allow us to assess a cause and effect relationship. For example, we cannot establish whether a change in environmental factors drives symbiont metabolism, and in turn host physiology; or conversely, if a change in environment directly effects host physiology leading to a regulation of symbiont population. This point will be discussed more extensively below.

### Do symbiotic bacteria drive gene expression in *Bathymodiolus azoricus*?

Double symbiosis enables *B. azoricus *to colonize sulfide and/or methane rich environments, in which the primary production of the symbionts ensures a part of the host's nutrition. Additionally, these mussels are able to filter feed and can survive senescent vent conditions. Mixotrophy is a major advantage in highly variable environments. In the particular case of *B. azoricus*, the mussel has to host two different symbionts that are present in varying abundance in different individuals. The identification of genes showing a similar regulation according to SOX and/or MOX content in two contrasted populations should help to distinguish genes that are mainly regulated by symbiont content from those that are regulated by both symbiont content and environmental parameters. The microarray analysis showed a relatively low number of genes significantly regulated by either SOX (8%) or MOX (14%) content in the MG population compared to the Rb population which had 16% regulated by SOX content and 20% by MOX content. These results suggest that symbiont content is less influential on the transcriptome in mussels from MG. However, because of the lack of studies on this particular dual-symbiont model, we have no information about a potential competition between SOX and MOX symbionts, and in turn, how mussels control each kind of symbiont. One could hypothesize that this difference is partly due to the bathymetric position of the two vent fields. We noticed that very few genes seem to be commonly regulated by both SOX and MOX content at MG, only 1% of all genes, suggesting that each symbiont may affect different pathways in mussels inhabiting this vent field, compared to Rb where 5.6% of all genes are commonly influenced by SOX and MOX content. The MG vent field is located at a depth of 800 m versus 2300 m for the Rainbow vent field. Thus, the mussels at MG benefit from a higher particle flux [39] which lessens the contribution to carbon nutrition needed from the symbiont, and possibly also the impact of symbionts on the host's transcriptome. In contrast, the mussels at the deeper Rainbow vent field experience lower particle flux and rely more on symbionts to meet their carbon needs. The pattern of gene expression obtained in this study could reflect the relative carbon contribution of symbionts compared to the availability of particles to host nutrition.

While the number of sequences available for bivalves has increased dramatically during the past few years [40-43], very few genes are either fully annotated and/or functionally characterised, often leading to a mean proportion of unknown sequences higher than 50%. In a bivalve such as *B. azoricus*, it is particularly difficult to find a relationship between the regulation of gene expression and a symbiotic state, even if the gene was described as involved in symbiosis-related functions in other organisms. However, we identified several genes previously described in host/symbiont relationships in other marine models, such as the sea anemone *Anemonia viridis *[8], the squid *Euprymna scolopes *[44], and the hydrothermal tubeworms *Ridgeia piscesae *and *Riftia pachyptila *[4,6], and one could hypothesize that their roles are potentially quite similar in hydrothermal mussels. It is, for example, well established that participation of sugar residues and lectins is a major process in host-microorganism recognition [45]. In this study, we identified five lectins belonging to different families and showing significant regulation according to symbiont quantity or activity. Lectins are known to bind carbohydrate structures on foreign cells [46,47]. Previous work conducted on corals [48] showed that specific lectins may bind to both pathogens and algal symbionts, suggesting that lectins may have been co-opted from an ancient innate immune system into a role of selecting and maintaining the photosynthetic endosymbionts in host tissues. In our analysis, we also identified a lysozyme and observed that this gene is more expressed in mussels with a high SOX content (MG vent field). The ancestral function of this enzyme is in defense against pathogens by degrading bacterial wall [49,50], but its implication in digestion in ruminants [51] and mollusks [52] has also been demonstrated. Lysozyme is strongly involved in the control and maintenance of the bacterial flora in the aphid bacteriocytes [53] and in the digestion of chemoautotrophic bacteria by their deep-sea bivalve hosts [54]. This change of lysozyme function from anti-biotic defense to digestion may have arisen through convergent evolution *via *positive selection [55]. An example of such change has been recently identified in the Eastern oyster, *Crassostrea virginica *in the i-type lysozymes family [52]. The regulation of lysozyme and lectins in *B. azoricus *agrees with previous observations, an indication that these two gene families are potentially good candidates for proteins that might be involved in the control and maintenance of symbionts. We also noticed the regulation of several genes directly or indirectly implicated in immune defense and inflammatory reaction. Among them are some receptors to melatonin [56], acetylcholine and laminin [57], synthaxin [58], kininogen, cystatin [59] and prostaglandin E2 synthetase and receptor [60], and all are significantly regulated by symbiont abundance and/or activity in *B. azoricus*. However, due to the multi-functionality of these proteins coupled with a lack of knowledge about their roles in hydrothermal mussels, we cannot be conclusive about their respective function(s) in the mussel/symbiont relationship. Complementary analyses of function and biochemical properties should help to determine to what extent these proteins are involved in the breakdown of symbionts and the elimination of microbial intruders in hydrothermal vent mussels.

The influence of bacteria on the cytoskeleton of host cells has been extensively studied in both host-pathogen interactions [61,62], and host-symbiont relationships [44,63,64]. These studies showed that various pathogens and symbionts increase their intimacy with the host tissues by altering the host cytoskeleton. For example, several microfilament and microtubule proteins are strongly regulated at both RNA and protein stages during the establishment of the symbiotic association between the squid *E. scolopes *and *Vibrio fischeri *[44,63]. In *B. azoricus*, some genes encoding cytoskeleton proteins are differentially regulated according to symbiont content, suggesting a potential effect of symbionts on host cell structure. Among these genes, five (tubulin, dynein, annexin, beta-thymosin and actin-related protein 2/3) present an interesting pattern of up-regulation in mussels hosting a high symbiont content, especially those with a high MOX level.

### Disentangling environment and symbiont effects on host gene expression: what is the order of event?

We established that the expression of several genes is correlated with either symbionts (quantity and/or activity) or environmental factors, but we were not able to determine which factor is directly responsible for transcriptome variations in mussels. The analysis of the transcriptome of a symbiotic organism often generates confusion when considering the combined effect of both symbionts and interrelated environmental factors. While they used a robust experimental design, DeSalvo et al. [65] were not able to determine if a thermal challenge changed coral (*Montastraea faveolata*) physiology which, in turn, induced a change in symbiont type dominance, or if a thermal stress directly changed symbiont type dominance and, in turn, the physiology of the host. In our case, we could assume that environmental factors directly influenced symbiont abundance in mussels [present study; [23]]. But we also observed a large inter-individual variation in symbiont abundance (measured at both sites), indicating that environmental factors alone do not drive symbiont quantity, but probably in association with host and/or symbiont need and/or physiological state.

The expression pattern of several genes was also ambiguous. For example, ferritins are significantly regulated by symbiont content in both populations of vent mussels, and their regulation in host-pathogen as well as in host-symbiont interactions has been previously demonstrated [66,67]. But ferritins are also known to play a pivotal role in iron homeostasis and the oxidative stress response. In our study, ferritins are more expressed in mussels harboring high SOX and MOX content collected at Rb vent field, compared to MG mussels in which ferritins are more expressed in low MOX content mussels. In this case, it was not possible to link the ferritin expression pattern to either symbiont content or the high level of iron measured at Rb vent field. A similar analysis applies to carbonic anhydrase (CA). We showed that CA is regulated by environmental factors (see discussion above). But, it has previously been demonstrated that CA plays a major role in transport and supply of CO_2 _to autotrophic symbionts housed in host tissues, such as in the two hydrothermal worms *R. pachyptila *and *R. piscesae *[6,68-70]. In these two species, the metabolism of the thiotrophic symbionts (sulphide oxidation) entails a fast and high production of protons, with which the worms have to cope, partly by induction of CA at both the level of enzymatic activity and mRNA expression. In our study, we observed that CA is highly expressed in the mussels collected at MG in which a higher SOX content was observed compared to Rb.

## Conclusion

In this study, we derived a list of candidate genes whose evolutionary trajectory in symbiont acquisition and host mechanisms for symbiont content regulation can now be explored. We also showed that in *B. azoricus*, the transcriptome appears to be regulated by symbiont content with a strong effect of vent field characteristics. However, we do not exclude that some of the genes in this study identified as being regulated could also be associated with parameters other than symbiont content and environment. Sampling and transport to the surface can modify transcriptome expression. However, we submit that those effects would be similar for all samples, mitigating their effect on the analysis. Adaptive evolution at the molecular level is more likely to be discovered from genes associated with regulatory networks underlying the expression of symbiosis related genes. Our study has produced a preliminary description of a transcriptomic response in a hydrothermal mussel symbiotic model, which we hope can help identify genes that progressively evolved to be involved in the acquisition and regulation of symbiosis on both ecological and evolutionary timescales.

## Methods

### Biological samples

The hydrothermal vent mussels, *B. azoricus*, were collected during the MoMARETO cruise [71] along the Mid-Atlantic Ridge with the N/O Pourquoi Pas? and ROV Victor 6000. Samples were collected at three vent fields, MG (37°50' N, 31°31' W; n = 25), LS (37°17' N, 32°17' W; n = 30) and Rb (36°14' N, 33°54' W; n = 25), which have contrasting physical and chemical characteristics (Table 1). Samples were collected at the end of the dive, kept in hermetic boxes containing vent seawater, brought onboard about 1.5 hours later, and immediately measured and dissected to minimize sampling effect. Harvested gill tissues were swiftly frozen in liquid nitrogen.

### Detection of symbiont quantity and symbiont gene expression by real-time PCR

#### Symbiont quantification

Genomic DNA of both mussel and bacteria was extracted together from gill tissue using a CTAB/PVP extraction procedure (2% CTAB, 1% PVP, 1.4 M NaCl, 0.2%^®^-mercaptoethanol, 100 mM Tris HCl pH8, 0.1 mg.mL^-1 ^proteinase K, 1 mg.mL^-1 ^lysozyme). After complete digestion of tissues (1 h at 60°C), the mixture was incubated with 1 μL of RNase for 30 min at 37°C. An equal volume of chloroform-isoamyl alcohol (24:1) was then added and tubes were slowly mixed by inversion for 3 min before a 10 min centrifugation at 14,000 rpm and 4°C. The supernatant was collected in a fresh tube, and DNA was precipitated with 2/3 volume of cold isopropanol (1 h at -20°C). The DNA pellet was recovered by centrifugation (14,000 rpm at 4°C for 20 min), washed with 75% cold ethanol, air-dried and suspended in 100 μL of sterile water. Genomic DNA from muscle was extracted by using the same protocol and used as a negative control in real-time PCR amplification. The relative quantity of symbionts was estimated by real-time PCR amplification using 16S specific primers designed according to the probes developed previously for FISH analysis [15] (Table 4). All experiments were carried out using a Chroma4 thermal cycler (Bio-Rad Laboratories Inc, Hercules, CA) and 1× ABsolute™QPCR SYBR^® ^Green mix (ABgene, Epsom, UK), 70 nM of each primer, and diluted DNA (2.5 ng) in a final volume of 10 μl. A 120 bp-fragment of cytosolic malate dehydrogenase gene (MDH) from the host was used as an internal PCR control (Table 4). The relative quantity of each symbiont type was estimated by using the comparative Ct method using the formula: RQ = 2^-⊗Ct ^(⊗Ct = Ct_16S_-Ct_MDH_). No amplification of MOX or SOX 16S was recorded when muscle genomic DNA (negative control) was used in amplification reactions. Significant differences in bacteria content between vent fields were detected with a nonparametric Wilcoxon-Mann-Whitney test with multiple test correction of Holm [72] (R-language 'stats' package).

**Table 4:** Primers used in real-time PCR amplification of bacteria and host gene.

Gene	Primer sequence 5'-3'
**Sulfide oxidizer symbiont 16S**	Forward GAGTAACGCGTAGGAATCTGC
	Reverse CGAAGGTCCTCCACTTTACTCCATAGAG
**Methanotrophic symbiont 16S**	Forward GTGCCAGCMGCCGCGGTAA
	Reverse GCTCCGCCACTAAGCCTATAAATAGACC
**Cytosolic malate dehydrogenase **(host)	Forward ATGGAGGAAAGAGATATGGCACTGAGCGT
	Reverse TAACATTAAACATAGCCTAGGAACCTAATG
**ATP sulfurylase **(SOX)	Forward GTGCGTGATGCCGCTATCCGCACCATG
	Reverse GGTCCGGCATAGAGCATGTCAAACGGATA
**Particulate methane monooxygenase A **(MOX)	Forward GAGTGGATTAACAGATATTTGAACTTCTGG
	Reverse CATACCACCAACAACAGCTGTAAGTACAAA
**Ribosomal protein L15 **(host)	Forward TATGGTAAACCTAAGACACAAGGAGT
	Reverse TGGAATGGATCAATCAAAATGATTTC

#### Symbiont gene expression

*We study the *expression of two bacterial genes, the ATP sulfurylase which is specific to SOX and catalyses the reaction of sulfate at the expense of ATP to generate adenosine phosphosulfate and the particulate methane monoxygenase pmoA which is specific to MOX and involved in methane oxidation) was followed to estimate the activity of each symbiont. Total RNA of both mussels and bacteria was extracted together from gill tissue by using Tri-Reagent (Sigma, St. Louis, MO) according to the manufacturer's instructions. Five μg of total RNA were reverse transcripted using M-MLV reverse transcriptase (Promega, Madison, WI), random hexamers (Promega) and an anchor-oligo(dT) primer (5'-CGCTCTAGAACTAGTGGATCT_(17)_-3'). The relative gene expression of symbionts was estimated by real-time PCR amplification using specific ATP sulfurylase and pmoA primers (Table 4; GenBank accession numbers AB178052 and AY945761, respectively). A volume of 4.6 μl of each diluted reverse transcription product (1:20) was subjected to real-time PCR in a final volume of 10 μl containing 70 nM of primers and 1× ABsolute™ QPCR SYBR^® ^Green Mix (ABgene). The amplification was carried out as follows: initial enzyme activation at 94°C for 15 min, then 40 cycles of 94°C for 15 sec and 60°C for 1 min. A fragment of ribosomal protein L15 gene (RpL15) from the host was used as an internal PCR control (Table 4). Relative expression of each gene was calculated according to comparative Ct method using the formula: RQ = 2^-⊗Ct^(⊗Ct = Ct_ATP sulf or pmoA_-Ct_RpL15_). Significant differences in bacteria gene expression between vent fields were detected by using a non parametric Wilcoxon Mann-Whitney test with multiple test correction of Holm [72] (R package).

### Suppressive Subtraction Hybridization (SSH)

Mussels from three hydrothermal vent fields Menez Gwen, Lucky Strike and Rainbow have been used in the SSH design in order to optimize the chance to characterize genes potentially regulated by symbiont content but also by environmental parameters. Total RNA was isolated from frozen gill tissues with Tri Reagent following the manufacturer's instructions (Sigma). Four mussel groups, named S+ (n = 3, high SOX content), M+ (n = 3, high MOX content), M- (n = 3, low MOX content) and SM- (n = 8, low SOX and MOX content), were created based on their respective bacteria content (Figure 1) and used in the following suppression subtraction hybridization (SSH) design: S+ ↔ M+, S+ ↔ M-, S+ ↔ SM-, M+ ↔ M- and M+ ↔ SM-, with S (+/-) and M (+/-) designated the level of SOX and MOX, respectively. Poly(A+) RNA were isolated from each of the 4 pools of total RNA using the PolyATract^® ^mRNA Isolation system (Promega) following the manufacturer's instructions. The SSH were obtained by using the PCR-Select™ cDNA Subtraction kit (Clontech, Mountain View, CA), amplified with Advantage^® ^cDNA PCR kit, and finally cloned into pGEM^®^-T vector (Promega) following the manufacturer's instructions. The ligation mixtures were used to transform DH5〈*E. coli *competent cells and colonies were then grown in liquid ampicillin-LB medium supplemented with 7.5% glycerol. Bacteria cultures were transferred to 384-plates and the sequencing of a total of 3840 clones was performed at Genoscope (Evry, France) using an ABI 3730 automatic capillary sequencer and the ABI BigDye Terminator v.3.1 sequencing kit.

### Sequence annotation

Prior to clustering and contig construction, the sequence traces were analyzed and trimmed of low quality 5' and 3' extremities (quality value <15), using the phred software [73,74]. Sequences were cleaned to remove low complexity regions, short length (<100 bp), and vector and adaptor sequence using seqclean [75]. Clustering and contig construction was performed using the TGICL software from TIGR [75]. Contig and singleton sequences were compared to protein sequences of the UniprotKB database [76] using BLASTX [77]. Coding frames were deduced from BLASTX best hit alignments (E-value ≤ 1e-03) and the CDS were created according to the protocol detailed in Gagniere et al. [78]. The protein sequences were then aligned to their homologs using the PipeAlign toolkit [79]. Gene Ontology (GO) [80] annotations for the *B. azoricus *sequences were provided by GOAnno [81] after analysis of the GO terms mined from the protein family.

### cDNA microarray preparation, hybridization and analysis

For this study, we used a microarray containing 3425 clones from *B. azoricus*: clones issued from the present SSH libraries and from a previous cDNA library [41]. Protocols for slide printing, hybridization and analysis of the microarray were carried out according to those established by the Plateforme Puces à ADN (Biogenouest^®^, Nantes, France, http://cardioserve.nantes.inserm.fr/ptf-puce). All clones have been submitted to PCR and purified according to standard protocols. Microarray slides were then printed with a Lucidea Arrayer (Amersham) on Epoxy slides. Each slide contained a total of 3425 features spotted. Printed slides were stored in a dark cool dry location until use.

#### Microarray hybridizations

A quantity of 20 μg of total RNA of each sample was directly labeled by reverse transcription (using random hexamer and dT primer) using a master mix containing 1 nmol of Cy5 or Cy3 dUTP (GE Healthcare). A loop design was used (one individual from Menez Gwen vent field against one individual from Rainbow vent field) in order to generate a replicate for each sample (dye swap). Following RT, single-stranded RNA was treated with RNAse A. Then, RT reactions were cleaned using Illustra CyScribe GFX purification Kit (GE Healthcare). Equimolar amounts of cDNA from both samples were mixed in a single pool with hybridization buffer, boiled for 2 min at 99°C then placed at 37°C for 30 min. Hybridization took place in Corning hybridization chambers overnight at 42°C. Microarrays were washed once in 2× SSC and 0.1% SDS followed by a rinse in 1× SSC and two rinses in 0.2× SSC and finally dried by centrifugation.

#### Microarray scanning and normalization

Slides were immediately scanned after centrifugation using an Axon 4000B scanner (Axon Instruments Inc.) with standard dual laser excitation at 532 nm (17 mW) and 635 nm (10 mW) according to the following parameters: Cy 5 Photo Multiplier Tube (PMT) 570 and Cy 3 PMT 610. This process was repeated for each of the 24 hybridized slides with a 5-μm resolution mode. The images (16-bit TIF images) were then analyzed with Genepix pro 5.1 software (Axon Instruments Inc.) according to the manufacturer's instructions. The spot density files output from GenePix Pro 6.0 were analyzed by eyes to remove bad spots. The normalization was then carried out using the programming language R/BioConductor [82] and Limma library [83]. The background correction of the probe intensity was carried out using the normexp method [84]. Then lowess normalization, intra- and inter-slide normalization were applied to remove intensity dependent trends. Replicated values of each gene were then averaged. The data obtained from the microarray and used in the following analysis have been deposited in Gene Expression Omnibus (GEO) at the National Center for Biotechnology Information (NCBI), with the series accession number (under process).

#### Microarray data analysis

All genes kept for analysis were used for hierarchical clustering analysis using TmeV [85] (http://www.tm4.org/mev.html) with Pearson correlation and complete linkage clustering parameters. KMC support parameters were used to identify clusters of genes that behave most similarly in all samples. Differentially expressed genes were identified by significance analysis of microarray using a fold-change of 2. False-discovery rate is estimated by analyzing permutations of the measurements and expresses the percentage of genes identified as significant by chance for a given value of a threshold parameter delta. This rate was manually adjusted to zero in order to only include a reasonable number of candidate genes with acceptable and well-defined error probabilities.

## Authors' contributions

IB and AT conceived and supervised the study. RR, OL and EC analysed the sequence data. RR and OL developed the web-data base. IB analysed the microarray data and wrote the manuscript. CD produced all sequences. AT participated to the microarray construction. FL supervised the study and participated in its coordination. All authors read and approved the final manuscript.

## Supplementary Material

Additional file 1 **Tables including showing genes presenting a differential expression in the analyses conducted (SOX/MOX content and quantity, hydrothermal vent origin)**. Click here for file
