# Supplementary material for: Conjugating effects of symbionts and environmental factors on gene expression in deep-sea hydrothermal vent mussels
Source: BMC Genomics. 2011 Oct 28;12:530. doi: 10.1186/1471-2164-12-530 (PMC3218092; doi:10.1186/1471-2164-12-530)
Supplement: Additional file 1 — Tables including showing genes presenting a differential expression in the analyses conducted (SOX/MOX content and quantity, hydrothermal vent origin). [file 1471-2164-12-530-S1.DOC]

Table S1. Genes presented a higher expression at Rb than at MG.

| EST76; EST108; EST179; EST183; EST214; EST263; EST277; EST320; EST384; EST391; EST395; EST449; EST597; EST612; EST800; EST881; EST981; EST1153; EST1186; EST1199; EST1380 |
| --- |
| 4 sequences no_hits_found |
| Q3zlc7 *oreochromis mossambicus* (mozambique tilapia) (tilapia mossambica). selenoprotein w2a. |
| Q4ykd0 *plasmodium berghei*. hypothetical protein. |
| Q4tes1 *tetraodon nigroviridis* (green puffer). chromosome undetermined scaf5157, whole genome shotgun sequence. (fragment). |
| Q7z2b5 *strongylocentrotus purpuratus* (purple sea urchin). polycystic kidney disease protein 2. |
| Q8is80 *euprymna scolopes*. 60s acidic ribosomal protein (fragment). |
| Q5m9l3 *mus musculus* (mouse). ribosomal protein s21 |
| AGAP007503-PA [*Anopheles gambiae* str. PEST] |
| K1033_MOUSE Uncharacterized protein KIAA1033 - *Mus musculus* (Mouse) |
| RPM1_CAEEL E3 ubiquitin-protein ligase rpm-1 - *Caenorhabditis elegans* |
| PIGM_ARATH GPI mannosyltransferase 1 - *Arabidopsis thaliana* (Mouse-ear cress) |
| UNC5D_MOUSE Netrin receptor UNC5D precursor - *Mus musculus* (Mouse) |
| GCP_PROMS Probable O-sialoglycoprotein endopeptidase - *Prochlorococcus marinus* (strain AS9601) |
| RL36_IXOSC 60S ribosomal protein L36 - *Ixodes scapularis* (Black-legged tick) (Deer tick) |
| apical endosomal glycoprotein [*Strongylocentrotus purpuratus*] |
| 209L2_MACMU CD209 antigen-like protein 2 - *Macaca mulatta* (Rhesus macaque) |
| IBPL1_MOUSE Insulin-like growth factor-binding protein-like 1 precursor - *Mus musculus* (Mouse) |
| RPOB_TOXGO DNA-directed RNA polymerase subunit beta - *Toxoplasma gondii* |
| FYV10_COCIM Protein FYV10 - *Coccidioides immitis* |
| Q70sh0 cepaea hortensis. sialic acid binding lectin precursor. |
| NOT1_SCHPO General negative regulator of transcription subunit 1-*Schizosaccharomyces pombe* (Fission yeast) |
| GO:0000151; C:ubiquitin ligase complex; IEA GO:0016567; P:protein ubiquitination; IEA |
| GO:0005840; C:ribosome; IEA GO:0006412; P:protein biosynthesis; IEA |
| PHLPP_RAT PH domain leucine-rich repeat protein phosphatase - *Rattus norvegicus* (Rat) |
| P41824 *aplysia californica* (california sea hare). y-box factor homolog (apy1). |
| NU3M_MICPE NADH-ubiquinone oxidoreductase chain 3 - *Microtus pennsylvanicus* (Meadow vole) |

Table S2. Genes presented a higher expression at MG than at Rb.

| EST81; EST113; EST256; EST314; EST376; EST461; EST467; EST545; EST722; EST752; EST824; EST873; EST879; EST909; EST932; EST995; EST1000; EST1065; EST1145; EST1210; EST1237; EST1245; EST1299; EST1365; EST1372; EST1439 |
| --- |
| 7 sequences no_hits_found |
| ATP9_MANSE ATP synthase lipid-binding protein, mitochondrial precursor - *Manduca sexta* (Tobacco hawkmoth) (Tobacco hornworm) |
| CAH6_CANFA Carbonic anhydrase 6 precursor - *Canis familiaris* (Dog) |
| CC130_DICDI Coiled-coil domain-containing protein 130 homolog - *Dictyostelium discoideum* (Slime mold) |
| CO1A1_CYNPY Collagen alpha-1(I) chain precursor - *Cynops pyrrhogaster* (Japanese common newt) |
| COBA2_MOUSE Collagen alpha-2(XI) chain precursor - *Mus musculus* (Mouse) |
| DAPF_BUCBP Diaminopimelate epimerase - *Buchnera aphidicola* subsp. *Baizongia pistaciae* |
| DDX17_MOUSE Probable ATP-dependent RNA helicase DDX17 - *Mus musculus* (Mouse) |
| DUS7_RAT Dual specificity protein phosphatase 7 - *Rattus norvegicus* (Rat) |
| FADD_BOVIN Protein FADD - *Bos taurus* (Bovine) |
| GO:0005737; C:cytoplasm; IEA GO:0008152; P:metabolism; IEA |
| GO:0005743; C:mitochondrial inner membrane; IEA GO:0006120; P:mitochondrial electron transport, NADH |
| GO:0016459; C:myosin; IEA GO:0003774; F:motor activity; IEA |
| GO:0016491; F:oxidoreductase activity; IEA GO:0008152; P:metabolism; IEA |
| HMX2_HUMAN Homeobox protein HMX2 - *Homo sapiens* (Human) |
| IMB_DROME Importin subunit beta - *Drosophila melanogaster* (Fruit fly) |
| KARG_LIOJA Arginine kinase - *Liolophura japonica* (Acanthopleura japonica) |
| LAB_XENLA Lupus La protein homolog B - *Xenopus laevis* (African clawed frog) |
| LACR_STAXY Lactose operon transcription activator - *Staphylococcus xylosus* |
| O08815 *rattus norvegicus* (rat). protein kinase. 3/2004 |
| POMP_BOVIN Proteasome maturation protein - *Bos taurus* (Bovine) |
| Q32np0 *xenopus laevis* (african clawed frog). hypothetical protein. |
| Q4fk29 m clecsf9 protein (bone marrow macrophage cdna, riken full-length enriched library, clone:i830018a02 product:c-type (calcium dependent, carbohydrate recognition domain) lectin, superfamily member 9, full insert sequence) (bone marrow mac). |
| Q4l223 *penaeus monodon* (penoeid shrimp). signal transducer and activator of transcription. |
| Q503m5 *brachydanio rerio* (zebrafish) (danio rerio). loc569147 protein (fragment). |
| Q5bl46 *xenopus tropicalis* (western clawed frog) (*silurana tropicalis*). lsm2 homolog, u6 small nuclear rna associated |
| Q5bn45 *mus musculus* (mouse). pierce 1 (1700007k13rik protein). |
| Q640k1 *xenopus laevis* (african clawed frog). loc494655 protein. |
| Q6usc1 *chlamys farreri*. cyclophilin a. |
| Q9czj2 *mus musculus* (mouse). heat shock 70 kda protein 12b. |
| Q9i7v8 *drosophila melanogaster* (fruit fly). cg9696-pa, isoform a. |
| Q9vay2 *drosophila melanogaster* (fruit fly). cg5520-pa (ld23641p). |
| RF1_CLONN Peptide chain release factor 1 - *Clostridium novyi* (strain NT) |
| RL7_DROME 60S ribosomal protein L7 - *Drosophila melanogaster* (Fruit fly) |
| SWF1_SCHPO Palmitoyltransferase swf1 - *Schizosaccharomyces pombe* (Fission yeast) |
| TCPA_DROME T-complex protein 1 subunit alpha - *Drosophila melanogaster* (Fruit fly) |
| TEKT3_MOUSE Tektin-3 - *Mus musculus* (Mouse) |
| YBH7_YEAST Putative uncharacterized protein YBL077W - *Saccharomyces cerevisiae* (Baker's yeast) |

Table S3. Genes with higher expression in MG mussels with high SOX content

| EST112; EST157; EST203; EST207; EST276; EST294; EST297; EST314; EST325; EST355; EST363; EST369; EST37; EST374; EST381; EST385; EST406; EST418; EST437; EST463; EST464; EST470; EST481; EST486; EST498; EST521; EST523; EST572; EST579; EST593; EST597; EST608; EST686; EST687; EST703; EST765; EST775; EST783; EST784; EST795; EST812; EST817; EST829; EST830; EST832; EST946; EST1102; EST1127; EST1137; EST1171; EST1191; EST1224; EST1237; EST1257; EST1282; EST1290; EST1298; EST1328; EST1330; EST1339; EST1343; EST1382; EST1402; EST1412; EST1440; EST1460; EST1491 |
| --- |
| 4 sequences no_hits_found |
| Aminoadipate aminotransferase [*Bos taurus*] |
| ATRX_MACEU Transcriptional regulator ATRX - *Macropus eugenii* (Tammar wallaby) |
| CHIA_RAT Acidic mammalian chitinase precursor - *Rattus norvegicus* (Rat) |
| CSK21_YEAST Casein kinase II subunit alpha - *Saccharomyces cerevisiae* (Baker's yeast) |
| CSLC4_ARATH Xyloglucan glycosyltransferase 4 - *Arabidopsis thaliana* (Mouse-ear cress) |
| DPO3B_CHLTR DNA polymerase III subunit beta - *Chlamydia trachomatis* |
| EIF3G_MOUSE Eukaryotic translation initiation factor 3 subunit G - *Mus musculus* (Mouse) |
| FADS2_DANRE Fatty acid desaturase 2 - *Danio rerio* (Zebrafish) (*Brachydanio rerio*) |
| FTSK_BORBU DNA translocase ftsK - *Borrelia burgdorferi* (Lyme disease spirochete) |
| GO:0005529; F:sugar binding; IEA |
| GO:0006950; P:response to stress; IEA |
| GO:0016020; C:membrane; IEA GO:0008654; P:phospholipid biosynthesis; IEA |
| GR58B_DROME Putative gustatory receptor 58b - *Drosophila melanogaster* (Fruit fly) |
| H33_XENTR Histone H3.3 - *Xenopus tropicalis* (Western clawed frog) (*Silurana tropicalis*) |
| HM18_CAEEL Homeobox protein ceh-18 - *Caenorhabditis elegans* |
| HS12A_MOUSE Heat shock 70 kDa protein 12A - *Mus musculus* (Mouse) |
| IAP3_NPVOP Apoptosis inhibitor 3 - *Orgyia pseudotsugata* multicapsid polyhedrosis virus (OpMNPV) |
| K1033_MOUSE Uncharacterized protein KIAA1033 - *Mus musculus* (Mouse) |
| MATK_ATRJA Maturase K - *Atractylodes japonica* |
| NCAN_RAT Neurocan core protein precursor - *Rattus norvegicus* (Rat) |
| NUD1_YEAST Protein NUD1 - *Saccharomyces cerevisiae* (Baker's yeast) |
| P00730 *bos taurus* (bovine). carboxypeptidase a1 precursor (ec 3.4.17.1). |
| P43143 *rattus norvegicus* (rat). neuronal acetylcholine receptor protein, alpha-6 subunit precursor. |
| P5CR3_MOUSE Pyrroline-5-carboxylate reductase 3 - *Mus musculus* (Mouse) |
| P91387 *caenorhabditis elegans*. hypothetical protein k12d9.1. |
| Q3hri5 *mytilus edulis* (blue mussel). x-box binding protein 1 (fragment). |
| Q3t0v7 *bos taurus* (bovine). mgc127033 protein. |
| Q3yl59 *pinctada fucata* (pearl oyster). mantle gene 7 (mantle gene 1). |
| Q4fzm5 *xenopus laevis* (african clawed frog). hypothetical protein. |
| Q4pm01 *ixodes scapularis* (black-legged tick) (deer tick). sorting nexin 3. |
| Q4pm80 *ixodes scapularis* (black-legged tick) (deer tick). atp synthase c subunit. |
| Q4rf58 *tetraodon nigroviridis* (green puffer). chromosome 14 scaf15120, whole genome shotgun sequence. |
| Q4spx2 *tetraodon nigroviridis* (green puffer). chromosome 7 scaf14536, whole genome shotgun sequence. (fragment). |
| Q4vbt4 *brachydanio rerio* (zebrafish) (danio rerio). translation elongation factor 1-gamma. |
| Q54ql8 *dictyostelium discoideum* (slime mold). hypothetical protein. |
| Q5blj9 m ribosomal protein l27 (10, 11 days embryo whole body cdna, riken full- length enriched library, clone:2810446a16 product:ribosomal protein l27, full insert sequence) (crl-1722 l5178y-r cdna, riken full-length enriched library, clone:i7. |
| Q5dbb2 *schistosoma japonicum* (blood fluke). sjchgc04204 protein. |
| Q5f424 *gallus gallus* (chicken). hypothetical protein. |
| Q5snx8 *brachydanio rerio* (zebrafish) (*danio rerio*). novel protein similar to vertebrate lectin, galactose binding, soluble 8 (lgals8) (fragment). |
| Q61hc5 *caenorhabditis briggsae*. hypothetical protein cbg10794. |
| Q6ddm3 *xenopus laevis* (african clawed frog). pecr-prov protein. |
| Q6ddx3 *xenopus laevis* (african clawed frog). mgc81610 protein. |
| Q6gq33 *xenopus laevis* (african clawed frog). mgc80379 protein. |
| Q6pw74 *capitella capitata*. cytochrome p450 cyp331a1. |
| Q7pyg5 *anopheles gambiae* str. pest. ensangp00000018356 (fragment). |
| Q7sz08 *xenopus laevis* (african clawed frog). mgc64332 protein. |
| Q7t2q9 *brachydanio rerio* (zebrafish) (*danio rerio*). transketolase (ec 2.2.1.1). |
| Q8i8y1 *manduca sexta* (tobacco hawkmoth) (tobacco hornworm). laccase 1 (ec 1.10.3.2). |
| Q8itu1 *Bat azoricus*. lysozyme. |
| Q95241 s amyloid beta a4 protein precursor (app) (alzheimer's disease amyloid protein homolog) [contains: soluble app-alpha (s-app-alpha); soluble app-beta (s-app-beta); c99; beta-amyloid protein 42 (beta-app42); beta-amyloid protein 40 (beta-a. |
| Q95w89 *branchiostoma belcheri* (amphoxius). 60s ribosomal protein l37a. |
| Q9i9e4 *pleurodeles waltlii* (iberian ribbed newt). putative nuclear movement protein pnudc. |
| Q9jix0 *mus musculus* (mouse). e(y)2 protein (12 days embryo male wolffian duct includes surrounding region cdna, riken full-length enriched library, clone:6720481i12 product:e(y)2 homolog (dc6) homolog). |
| Q9lgz9 *arabidopsis thaliana* (mouse-ear cress). arabidopsis thaliana genomic dna, chromosome 3, bac clone:f1d9. |
| Q9vji8 *drosophila melanogaster* (fruit fly). cg17905-pa (re01566p). |
| Q9vq62 *drosophila melanogaster* (fruit fly). hypothetical protein npc2 precursor (niemann pick type c2 protein homolog). |
| Q9z1p6 *mus musculus* (mouse). nadh-ubiquinone oxidoreductase subunit b14.5a (ec 1.6.5.3) (ec 1.6.99.3) (complex i-b14.5a) (ci-b14.5a). |
| RPOB_PLAFA DNA-directed RNA polymerase subunit beta - *Plasmodium falciparum* |
| similar to odorant receptor [*Danio rerio*] |
| SO1A5_RAT Solute carrier organic anion transporter family member 1A5 - *Rattus norvegicus* (Rat) |
| TOR_CAEEL Target of rapamycin homolog - *Caenorhabditis elegans* |
| Y591_RICPR Uncharacterized protein RP591 - *Rickettsia prowazekii* |

Table S4. Genes with higher expression in MG mussels with low SOX content

| EST1048; EST1051; EST1062; EST107; EST1101; EST1112; EST1115; EST1145; EST1159; EST1213; EST1233; EST1245; EST13; EST130; EST1358; EST1367; EST1403; EST144; EST1443; EST1526; EST185; EST214; EST271; EST280; EST31; EST329; EST377; EST431; EST438; EST49; EST562; EST60; EST610; EST625; EST627; EST639; EST645; EST651; EST704; EST718; EST764; EST792; EST8; EST801; EST876; EST887; EST891; EST916; EST922; EST995 |
| --- |
| 7 sequences no_hits_found |
| ACT_CRAGI Actin - *Crassostrea gigas* (Pacific oyster) (Crassostrea angulata) |
| ANK3_HUMAN Ankyrin-3 - *Homo sapiens* (Human) |
| ANT3_SHEEP Antithrombin-III precursor - *Ovis aries* (Sheep) |
| ARLY_RAT Argininosuccinate lyase - *Rattus norvegicus* (Rat) |
| CA151_MOUSE UPF0327 protein C1orf151 homolog - *Mus musculus* (Mouse) |
| CAPSD_TTVV9 Capsid protein - *Torque teno* virus (isolate Japanese macaque/Japan/Mf-TTV9/2000) (TTV) |
| DAF19_CAEEL RFX-like transcription factor daf-19 - *Caenorhabditis elegans* |
| DDC_RAT Aromatic-L-amino-acid decarboxylase - *Rattus norvegicus* (Rat) |
| DISP1_DANRE Protein dispatched homolog 1 - *Danio rerio* (Zebrafish) (*Brachydanio rerio*) |
| EAA3_MOUSE Excitatory amino acid transporter 3 - *Mus musculus* (Mouse) |
| EFG1_TREDE Elongation factor G 1 - *Treponema denticola* |
| ENGB_LACDB Probable GTP-binding protein engB - *Lactobacillus delbrueckii* subsp. bulgaricus (strain ATCC BAA-365) |
| ENV_HV190 Envelope glycoprotein gp160 precursor - Human immunodeficiency virus type 1 (isolate 90CF056 group M subtype H) (HIV-1) |
| FOJO_DROME Protein four-jointed [Contains: Protein four-jointed, secreted isoform] - *Drosophila melanogaster* (Fruit fly) |
| glutathione peroxidase 2 [*Macaca mulatta*] |
| GO:0004252; F:serine-type endopeptidase activity; IEA GO:0006508; P:proteolysis; IEA |
| GO:0004563; F:beta-N-acetylhexosaminidase activity; IEA GO:0005975; P:carbohydrate metabolism; IEA |
| GO:0005840; C:ribosome; IEA GO:0006412; P:protein biosynthesis; IEA |
| GO:0016021; C:integral to membrane; IEA GO:0005488; F:binding; IEA |
| GYP6_YEAST GTPase-activating protein GYP6 - *Saccharomyces cerevisiae* (Baker's yeast) |
| HEM1_PROM9 Glutamyl-tRNA reductase - *Prochlorococcus marinus* (strain MIT 9312) |
| HS90B_DANRE Heat shock protein HSP 90-beta - *Danio rerio* (Zebrafish) (*Brachydanio rerio*) |
| hypothetical protein GSPATT00002614001 [*Paramecium tetraurelia* |
| ICB1_HUMAN Induced by contact to basement membrane 1 protein - *Homo sapiens* (Human) |
| KARG_LIOJA Arginine kinase - Liolophura japonica (*Acanthopleura japonica*) |
| MAK5_ASPTN ATP-dependent RNA helicase mak5 - *Aspergillus terreus* (strain NIH 2624) |
| MDMC_STRMY O-methyltransferase mdmC - *Streptomyces mycarofaciens* |
| MDTK_KLEP7 Multidrug resistance protein mdtK - *Klebsiella pneumoniae* subsp. *pneumoniae* (strain ATCC 700721 / MGH 78578) |
| MTER1_RAT mTERF domain-containing protein 1, mitochondrial precursor - *Rattus norvegicus* (Rat) |
| P82596 *haliotis laevigata* (abalone). perlucin. |
| PPIF_RAT Peptidyl-prolyl cis-trans isomerase, mitochondrial precursor - *Rattus norvegicus* (Rat) |
| Predicted acyl esterase [*Synechococcus* sp. WH 7803] |
| Q3t073 *bos taurus* (bovine). mgc127909 protein. |
| Q3uj73 *mus musculus* (mouse). cdna, riken full-length enriched library, clone:i920030a10 product:tubulin beta-2 chain homolog. |
| Q4s0q0 *tetraodon nigroviridis* (green puffer). chromosome 2 scaf14781, whole genome shotgun sequence. |
| Q4sbv8 *tetraodon nigroviridis* (green puffer). chromosome undetermined scaf14663, whole genome shotgun sequence. |
| Q56tz4 *rattus norvegicus* (rat). macrophage-inducible c-type lectin. |
| Q5bl46 *xenopus tropicalis* (western clawed frog) (*silurana tropicalis*). lsm2 homolog, u6 small nuclear rna associated. |
| Q5f1m8 *mytilus galloprovincialis* (mediterranean mussel). nadh dehydrogenase subunit 4. |
| Q5xi65 *rattus norvegicus* (rat). hypothetical protein mgc94915. |
| Q6glr8 *xenopus laevis* (african clawed frog). loc443706 protein (fragment). |
| Q76ik4 *ciona intestinalis*. pol-like protein. |
| Q86bu1 *musca domestica* (house fly). defensin. |
| Q8i6v6 *euprymna scolopes*. ganglioside gm2 activator (fragment). |
| Q8vcx5 m 7 days embryo whole body cdna, riken full-length enriched library, clone:c430019e07 product:similar to calcium binding atopy-related autoantigen 1 homolog (adult male liver tumor cdna, riken full-length enriched library, clone:c730016l. |
| Q8wp36 *suberites domuncula* (sponge). col protein. |
| Q8ws60 *branchiostoma floridae* (florida lancelet) (amphioxus). endonuclease/reverse transcriptase. |
| Q95nn1 *tribolium castaneum* (red flour beetle). tryptophan oxygenase. |
| Q9cpu2 *mus musculus* (mouse). nadh-ubiquinone oxidoreductase aggg subunit, mitochondrial precursor (ec 1.6.5.3) (ec 1.6.99.3) (complex i-aggg) (ci-aggg). |
| Q9vf83 *drosophila melanogaster* (fruit fly). cg31301-pa (ld33178p). |
| RIR1_CAEEL Ribonucleoside-diphosphate reductase large subunit - *Caenorhabditis elegans* |
| RL1_CARHZ 50S ribosomal protein L1 - *Carboxydothermus hydrogenoformans* (strain Z-2901 / DSM 6008) |
| RL12_RAT 60S ribosomal protein L12 - *Rattus norvegicus* (Rat) |
| RPOC2_PHYPA DNA-directed RNA polymerase subunit beta'' - *Physcomitrella patens* (Moss) |
| RS2_URECA 40S ribosomal protein S2 - *Urechis caupo* (Innkeeper worm) (Spoonworm) |
| RSMF_SHEON Ribosomal RNA small subunit methyltransferase F - *Shewanella oneidensis* |
| RUD3_YEAST GRIP domain-containing protein RUD3 - *Saccharomyces cerevisiae* (Baker's yeast) |
| SAR1B_BOVIN GTP-binding protein SAR1b - *Bos taurus* (Bovine) |
| SBP1_DANRE Selenium-binding protein 1 - *Danio rerio* (Zebrafish) (*Brachydanio rerio*) |
| SNX2_MOUSE Sorting nexin-2 - *Mus musculus* (Mouse) |
| STOM_HUMAN Erythrocyte band 7 integral membrane protein - *Homo sapiens* (Human) |
| T23O_XENTR Tryptophan 2,3-dioxygenase - *Xenopus tropicalis* (Western clawed frog) (Silurana tropicalis) |
| TCPB_MACFA T-complex protein 1 subunit beta - *Macaca fascicularis* (Crab eating macaque) (Cynomolgus monkey) |
| TEKT3_MOUSE Tektin-3 - *Mus musculus* (Mouse) |
| TRME_PROM0 tRNA modification GTPase trmE - *Prochlorococcus marinus* (strain MIT 9301) |
| UBIQ_XENLA Ubiquitin - *Xenopus laevis* (African clawed frog) |
| unnamed protein product [*Candida glabrata*] |
| Y223_MYCGE Uncharacterized protein MG223 - *Mycoplasma genitalium* |
| YCF1_LOTJA Putative membrane protein ycf1 - *Lotus japonicus* |
| YCX2_CYAPA Uncharacterized 24.3 kDa protein in psbH-rpl11 intergenic region - *Cyanophora paradoxa* |
| YKAA_CAEEL Uncharacterized amino-acid permease B0303.11 - *Caenorhabditis elegans* |

Table S5. Genes with higher expression in MG mussels with high MOX content

| EST1033; EST1048; EST1049; EST1054; EST1062; EST1070; EST1123; EST1161; EST1184; EST119; EST1200; EST1201; EST1205; EST1210; EST1213; EST1226; EST1228; EST1241; EST1259; EST126; EST1263; EST1264; EST1273; EST1299; EST1316; EST1318; EST1322; EST1327; EST1337; EST1350; EST1360; EST1365; EST1373; EST1379; EST1394; EST1400; EST1410; EST1414; EST1418; EST1426; EST143; EST1432; EST1437; EST1446; EST1448; EST1455; EST1459; EST1466; EST147; EST1470; EST1476; EST1477; EST1479; EST1481; EST1482; EST1486; EST1497; EST1509; EST1516; EST1520; EST1537; EST1539; EST1557; EST161; EST201; EST216; EST256; EST292; EST296; EST301; EST341; EST349; EST367; EST377; EST387; EST39; EST399; EST409; EST414; EST47; EST490; EST492; EST504; EST508; EST512; EST520; EST53; EST530; EST537; EST551; EST588; EST591; EST611; EST619; EST623; EST631; EST633; EST643; EST644; EST647; EST651; EST664; EST675; EST705; EST738; EST745; EST773; EST787; EST798; EST858; EST897; EST9; EST966; EST978; EST993 |
| --- |
| 17 sequences no_hits_found |
| AGAP007259-PA [*Anopheles gambiae* str. PEST] |
| AICDA_HUMAN Activation-induced cytidine deaminase - *Homo sapiens* (Human) |
| ALGL_PSEPF Alginate lyase precursor - *Pseudomonas fluorescens* (strain PfO-1) |
| ARPC3_HUMAN Actin-related protein 2/3 complex subunit 3 - *Homo sapiens* (Human) |
| ASPM_SAIBB Abnormal spindle-like microcephaly-associated protein homolog - *Saimiri boliviensis boliviensis* (Bolivian squirrel monkey) |
| ATM_YEAST Serine/threonine-protein kinase TEL1 - *Saccharomyces cerevisiae* (Baker's yeast) |
| ATPBM_HEVBR ATP synthase subunit beta, mitochondrial precursor - *Hevea brasiliensis* (Para rubber tree) |
| ATPK_DROME Putative ATP synthase subunit f, mitochondrial - *Drosophila melanogaster* (Fruit fly) |
| ATX1_PLAFA Probable cation-transporting ATPase 1 - *Plasmodium falciparum* |
| BSC6_YEAST Bypass of stop codon protein 6 - *Saccharomyces cerevisiae* (Baker's yeast) |
| CA151_MOUSE UPF0327 protein C1orf151 homolog - *Mus musculus* (Mouse) |
| CAH1_MONDO Carbonic anhydrase 1 - *Monodelphis domestica* (Short-tailed gray opossum) |
| calreticulin [*Crassostrea gigas*] |
| CC50A_CHICK Cell cycle control protein 50A - *Gallus gallus* (Chicken) |
| CF153_HUMAN UPF0399 protein C6orf153 - *Homo sapiens* (Human) |
| CLPH_ONCVO Calponin homolog OV9M - *Onchocerca volvulus* |
| COCA1_RABIT Collagen alpha-1(XII) chain - *Oryctolagus cuniculus* (Rabbit) |
| CQ061_BUFBG UPF0451 protein C17orf61 homolog precursor - *Bufo bufo gargarizans* (Asian toad) |
| CXA4_XENLA Gap junction alpha-4 protein - *Xenopus laevis* (African clawed frog) |
| DAF19_CAEEL RFX-like transcription factor daf-19 - *Caenorhabditis elegans* |
| DBF20_YEAST Serine/threonine-protein kinase DBF20 - *Saccharomyces cerevisiae* (Baker's yeast) |
| DHX36_HUMAN Probable ATP-dependent RNA helicase DHX36 - *Homo sapiens* (Human) |
| DISP1_DANRE Protein dispatched homolog 1 - *Danio rerio* (Zebrafish) (*Brachydanio rerio*) |
| DOCK2_MOUSE Dedicator of cytokinesis protein 2 - *Mus musculus* (Mouse) |
| DYHC_ONCMY Dynein heavy chain - *Oncorhynchus mykiss* (Rainbow trout) (*Salmo gairdneri*) |
| EBP_CAVPO 3-beta-hydroxysteroid-Delta(8),Delta(7)-isomerase - *Cavia porcellus* (Guinea pig) |
| EF1A_SPOFR Elongation factor 1-alpha - *Spodoptera frugiperda* (Fall armyworm) |
| ELP3_DANRE Elongator complex protein 3 - *Danio rerio (*Zebrafish) (*Brachydanio rerio*) |
| FLP_YEAST Site-specific recombinase Flp - *Saccharomyces cerevisiae* (Baker's yeast) |
| FOJO_DROME Protein four-jointed [Contains: Protein four-jointed, secreted isoform] - *Drosophila melanogaster* (Fruit fly) |
| glyceraldehyde-3-phosphate dehydrogenase, type I [*Clostridium cellulolyticum* H10] |
| GO:0005634; C:nucleus; IEA GO:0008270; F:zinc ion binding; IEA |
| GO:0016491; F:oxidoreductase activity; IEA GO:0008152; P:metabolism; IEA |
| HEY2_MOUSE Hairy/enhancer-of-split related with YRPW motif protein 2 - *Mus musculus* (Mouse) |
| HMX2_HUMAN Homeobox protein HMX2 - *Homo sapiens* (Human) |
| hypothetical protein PVX_080190 [*Plasmodium vivax* SaI-1] |
| hypothetical protein TA05475 [*Theileria annulata* strain Ankara] |
| IF2B_BOVIN Eukaryotic translation initiation factor 2 subunit 2 - *Bos taurus* (Bovine) |
| INSR_XENLA Insulin receptor precursor - *Xenopus laevis* (African clawed frog) |
| Interleukin 8 receptor, alpha [*Homo sapiens*] |
| KCC1_YEAST Calcium/calmodulin-dependent protein kinase I - *Saccharomyces cerevisiae* (Baker's yeast) |
| kruppel-like factor [*Lehmannia valentiana*] |
| LIMK2_HUMAN LIM domain kinase 2 - *Homo sapiens* (Human) |
| MET8_YEAST Siroheme biosynthesis protein MET8 [Includes: Precorrin-2 dehydrogenase - *Saccharomyces cerevisiae* (Baker's yeast) |
| MLTA_BUCAP Membrane-bound lytic murein transglycosylase A homolog - *Buchnera aphidicola* subsp. *Schizaphis graminum* |
| MTR1L_SHEEP Melatonin-related receptor - *Ovis aries* (Sheep) |
| MVP_DISOM Major vault protein - *Discopyge ommata* (Electric ray) |
| NOG1_RAT Nucleolar GTP-binding protein 1 - *Rattus norvegicus* (Rat) |
| NUDC_RAT Nuclear migration protein nudC - *Rattus norvegicus* (Rat) |
| O13075 *gallus gallus* (chicken). nuclear factor nf-kb1. |
| OPDA_ECOLI Oligopeptidase A - *Escherichia coli* (strain K12) |
| OXAA_BLOFL Inner membrane protein oxaA - *Blochmannia floridanus* |
| P35003 *haliotis rufescens* (california red abalone). chymotrypsin-like serine proteinase precursor (ec 3.4.21.-). |
| P56974 *mus musculus* (mouse). pro-neuregulin-2, membrane-bound isoform precursor (pro-nrg2) [contains: neuregulin-2 (nrg-2) (divergent of neuregulin 1) (don-1)]. |
| PAI2_RAT Plasminogen activator inhibitor 2 type A - *Rattus norvegicus* (Rat) |
| PMPI_CHLTR Probable outer membrane protein pmpI precursor - *Chlamydia trachomatis* |
| POMP_BOVIN Proteasome maturation protein - *Bos taurus* (Bovine) |
| predicted protein [*Nematostella vectensis*] |
| PTC1_CAEEL Protein patched homolog 1 - *Caenorhabditis elegans* |
| Q24537 *drosophila melanogaster* (fruit fly). high mobility group protein dsp1 (dorsal switch protein 1). |
| Q27802 *tripneustes gratilla* (hawaian sea urchin). dynein heavy chain isotype 1b (ec 3.6.1.3). |
| Q30hu9 *crassostrea virginica* (eastern oyster). serine protease inhibitor 1. |
| Q32n81 *xenopus laevis* (african clawed frog). hypothetical protein. |
| Q3t0c5 *bos taurus* (bovine). hypothetical protein. |
| Q3uj73 *mus musculus* (mouse). cdna, riken full-length enriched library, clone:i920030a10 product:tubulin beta-2 chain homolog. |
| Q4gx99 *biphyllus lunatus*. ribosomal protein l36e. |
| Q4gxm8 *georissus* sp. apv-2005. ribosomal protein l6e (fragment). |
| Q4pm17 *ixodes scapularis* (black-legged tick) (deer tick). ribosomal protein l35a. |
| Q4sh63 *tetraodon nigroviridis* (green puffer). chromosome 8 scaf14587, whole genome shotgun sequence. |
| Q4spx2 *tetraodon nigroviridis* (green puffer). chromosome 7 scaf14536, whole genome shotgun sequence. |
| Q4sw70 *tetraodon nigroviridis* (green puffer). chromosome 9 scaf13686, whole genome shotgun sequence. |
| Q4t624 *tetraodon nigroviridis* (green puffer). chromosome undetermined scaf8975, whole genome shotgun sequence. |
| Q4tmv7 *erythrobacter litoralis* htcc2594. hypothetical protein. |
| Q56tz4 *rattus norvegicus* (rat). macrophage-inducible c-type lectin. |
| Q5mkl0 *schistocerca gregaria* (desert locust). sparc. |
| Q5u275 *xenopus laevis* (african clawed frog). loc495666 protein |
| Q5u3u7 *brachydanio rerio* (zebrafish) (danio rerio). zgc:101615. |
| Q68er8 *xenopus tropicalis* (western clawed frog) (silurana tropicalis). mgc89305 protein. |
| Q6deg0 *brachydanio rerio* (zebrafish) (danio rerio). zgc:92367. |
| Q6e6j7 *cricetulus griseus* (chinese hamster). heterochromatin protein 1 beta. |
| Q6iwn5 *branchiostoma belcheri* tsingtaunese. ependymin related protein-1. |
| Q6lfd1 *plasmodium falciparum* (isolate 3d7). hypothetical protein. |
| Q6pc91 *brachydanio rerio* (zebrafish) (danio rerio). basic transcription factor 3-like 4. |
| Q6qm13 *lytechinus variegatus* (sea urchin). guanine nucleotide-binding protein g(q) alpha subunit (ec 3.6.5.1). |
| Q6usc1 *chlamys farreri*. cyclophilin a. |
| Q70mp2 *crassostrea gigas* (pacific oyster). ribosomal protein s3a (fragment). |
| Q7pqg7 *anopheles gambiae* str. pest. ensangp00000011832. |
| Q7yzz5 *oikopleura dioica*. putative alpha-tubulin. |
| Q803v5 *brachydanio rerio* (zebrafish) (*danio rerio*). zgc:55455 protein (g protein beta subunit-like). |
| Q86d04 *caenorhabditis elegans*. hypothetical protein col-135. |
| Q86iv5 *dictyostelium discoideum* (slime mold). similar to dictyostelium discoideum (slime mold). countin (component of the counting factor (cf) complex). |
| Q86lp9 *branchiostoma belcheri* (amphoxius). hypothetical gaba(a) receptor-associated protein like-2. |
| Q86pl3 *perinereis aibuhitensis* (clamworm). 40s ribosomal protein. |
| Q8avd5 *xenopus laevis* (african clawed frog). bzrp-prov protein. |
| Q8bfq8 m bc023835 protein (10 days neonate cerebellum cdna, riken full-length enriched library, clone:b930098g02 product:hypothetical thij/pfpi family containing protein, full insert sequence) (12 days embryo eyeball cdna, riken full-length enr. |
| Q8bmr6 *mus musculus* (mouse). 0 day neonate head cdna, riken full-length enriched library, clone:4833440h06 product:procollagen, type iii, alpha 1, full insert sequence. (fragment). |
| Q8cdt7 *mus musculus* (mouse). protein c10orf107 homolog. |
| Q8ita7 *aequipecten irradians* (bay scallop). ribosomal protein l21. |
| Q8k0a3 *mus musculus* (mouse). bai3 protein (fragment). |
| Q8mv00 *tribolium castaneum* (red flour beetle). polyubiquitin. |
| Q8r562 *mus musculus* (mouse). ribonucleoprotein. |
| Q8t697 *aplysia californica* (california sea hare). beta-thymosin (thymosin-beta). |
| Q8t783 *branchiostoma floridae* (florida lancelet) (amphioxus). hypothetical protein. |
| Q8tup8 *methanosarcina acetivorans*. dynein heavy chain. |
| Q8vhs2 *mus musculus* (mouse). crumbs protein homolog 1 precursor. |
| Q8wp36 *suberites domuncula* (sponge). col protein. |
| Q8ws60 *branchiostoma floridae* (florida lancelet) (amphioxus). endonuclease/reverse transcriptase. |
| Q92797 *homo sapiens* (human). symplekin. |
| Q94760 *strongylocentrotus purpuratus* (purple sea urchin). mitochondrial atp synthase alpha subunit precursor. |
| Q95v82 *rhyzopertha dominica* (lesser grain borer). cytochrome c oxidase subunit va (ec 1.9.3.1). |
| Q95w89 *branchiostoma belcheri* (amphoxius). 60s ribosomal protein l37a. |
| Q99jc0 *rattus norvegicus* (rat). rrna promoter binding protein. |
| Q9cqk5 m 18-day embryo whole body cdna, riken full-length enriched library, clone:1110002k21 product:hypothetical protein, full insert sequence (keratinocytes associated protein 2) (adult male kidney cdna, riken full-length enriched library, cl. |
| Q9gv70 *tegula pfeifferi* (pfeiffer's top shell). vitelline coat protein 41. |
| Q9ib90 *cyprinus carpio* (common carp). c-type lectin. |
| Q9jhg1 *mus musculus* (mouse). phosphatidylinositol n-acetylglucosaminyltransferase subunit p (ec 2.4.1.198) (phosphatidylinositol-glycan biosynthesis, class p protein) (pig-p) (down syndrome critical region protein 5 homolog). |
| Q9u5r6 *entodinium caudatum*. putative ribosomal protein (fragment). |
| Q9xnj8 Bat sp. nadh dehydrogenase subunit 4 (fragment). |
| Q9y156 *drosophila melanogaster* (fruit fly). cg4778-pa (bcdna.gh02976). |
| Q9z125 *mus musculus* (mouse). oasis protein. |
| RL18_RAT 60S ribosomal protein L18 - *Rattus norvegicus* (Rat) |
| RL21_RAT 60S ribosomal protein L21 - *Rattus norvegicus* (Rat) |
| RL34_AEDTR 60S ribosomal protein L34 - *Aedes triseriatus* (Mosquito) (Ochlerotatus triseriatus) |
| RL4_MOUSE 60S ribosomal protein L4 - *Mus musculus* (Mouse) |
| RL4_RAT 60S ribosomal protein L4 - *Rattus norvegicus* (Rat) |
| RPB4_MOUSE DNA-directed RNA polymerase II subunit RPB4 - *Mus musculus* (Mouse) |
| RR3_EPIVI Plastid 30S ribosomal protein S3 - *Epifagus virginiana* (Beechdrops) |
| RS14_DROME 40S ribosomal protein S14 - *Drosophila melanogaster* (Fruit fly) |
| RS17_BOVIN 40S ribosomal protein S17 - *Bos taurus* (Bovine) |
| RS7_DANRE 40S ribosomal protein S7 - *Danio rerio* (Zebrafish) (Brachydanio rerio) |
| RUD3_YEAST GRIP domain-containing protein RUD3 - *Saccharomyces cerevisiae* (Baker's yeast) |
| S230_PLAFO Transmission-blocking target antigen S230 precursor - *Plasmodium falciparum* (isolate NF54) |
| S27A6_HUMAN Long-chain fatty acid transport protein 6 - *Homo sapiens* (Human) |
| SAHH_CAEEL Adenosylhomocysteinase - *Caenorhabditis elegans* |
| SECA2_MYCTU Protein translocase subunit secA 2 - *Mycobacterium tuberculosis* |
| SPD2A_DANRE SH3 and PX domain-containing protein 2A - *Danio rerio* (Zebrafish) (*Brachydanio rerio*) |
| STOM_HUMAN Erythrocyte band 7 integral membrane protein - *Homo sapiens* (Human) |
| TCPA_DROME T-complex protein 1 subunit alpha - *Drosophila melanogaster* (Fruit fly) |
| transmembrane BAX inhibitor motif containing 4 [*Homo sapiens*] |
| TRME_PROM0 tRNA modification GTPase trmE - *Prochlorococcus marinus* (strain MIT 9301) |
| TYRO_STRAT Tyrosinase - *Streptomyces antibioticus* |
| UB17L_HUMAN Ubiquitin carboxyl-terminal hydrolase 17-like protein - *Homo sapiens* (Human) |
| unnamed protein product [*Candida glabrata*] |
| unnamed protein product [*Tetraodon nigroviridis*] |
| YBH7_YEAST Putative uncharacterized protein YBL077W - *Saccharomyces cerevisiae* (Baker's yeast) |
| YCF2_EPIVI Protein ycf2 - *Epifagus virginiana* (Beechdrops) |
| YCF2_PHYPA Protein ycf2 - *Physcomitrella patens* (Moss) |
| YCX2_CYAPA Uncharacterized 24.3 kDa protein in psbH-rpl11 intergenic region - *Cyanophora paradoxa* |
| YKAA_CAEEL Uncharacterized amino-acid permease B0303.11 - *Caenorhabditis elegans* |
| YNEF_ECOLI Uncharacterized protein yneF - *Escherichia coli* (strain K12) |
| YOR6_TTV1K Uncharacterized 8.9 kDa protein - *Thermoproteus tenax* virus 1 (strain KRA1) (TTV1) |
| ZDHC6_DICDI Putative ZDHHC-type palmitoyltransferase 6 - *Dictyostelium discoideum* (Slime mold) |
| ZFHX4_CHICK Zinc finger homeobox protein 4 - *Gallus gallus* (Chicken) |

Table S6. Genes with higher expression in MG mussels with low MOX content

| EST10; EST1006; EST1027; EST1071; EST1079; EST1091; EST1096; EST111; EST112; EST1130; EST1137; EST1186; EST1196; EST1199; EST1208; EST1230; EST1260; EST1278; EST1366; EST1464; EST1485; EST15; EST1511; EST1544; EST1549; EST155; EST159; EST171; EST177; EST178; EST179; EST184; EST192; EST20; EST202; EST203; EST21; EST228; EST232; EST241; EST246; EST263; EST264; EST265; EST277; EST278; EST30; EST319; EST324; EST328; EST385; EST413; EST437; EST459; EST475; EST487; EST517; EST54; EST541; EST544; EST564; EST58; EST635; EST652; EST668; EST679; EST687; EST693; EST75; EST759; EST82; EST825; EST84; EST886; EST898; EST910; EST911; EST923; EST928; EST936; EST975; EST994 |
| --- |
| 13 sequences no_hits_found |
| ACT_PLAMG Actin, adductor muscle - *Placopecten magellanicus* (Sea scallop) |
| ALMS1_MOUSE Alstrom syndrome protein 1 homolog - *Mus musculus* (Mouse) |
| ANX11_BOVIN Annexin A11 - *Bos taurus* (Bovine) |
| ATPG2_ARATH ATP synthase gamma chain 2, chloroplast precursor - *Arabidopsis thaliana* (Mouse-ear cress) |
| C518B_DICDI Probable cytochrome P450 518B1 - *Dictyostelium discoideum* (Slime mold) |
| COX3_LUMTE Cytochrome c oxidase subunit 3 - *Lumbricus terrestris* (Common earthworm) |
| CSLC4_ARATH Xyloglucan glycosyltransferase 4 - *Arabidopsis thaliana* (Mouse-ear cress) |
| DPO4_METAC DNA polymerase IV - *Methanosarcina acetivorans* |
| EAF7_YEAST Chromatin modification-related protein EAF7 - *Saccharomyces cerevisiae* (Baker's yeast) |
| EF2_PONPY Elongation factor 2 - *Pongo pygmaeus* (Bornean orangutan) |
| ERF1X_ARATH Eukaryotic peptide chain release factor subunit 1-1 - *Arabidopsis thaliana* (Mouse-ear cress) |
| FYV10_COCIM Protein FYV10 - *Coccidioides immitis* |
| GCP_PROMS Probable O-sialoglycoprotein endopeptidase - *Prochlorococcus marinus* (strain AS9601) |
| GO:0005515; F:protein binding; IPI |
| GO:0016021; C:integral to membrane; IEA GO:0015031; P:protein transport; IEA |
| HEM1_PROM9 Glutamyl-tRNA reductase - *Prochlorococcus marinus* (strain MIT 9312) |
| HSP7C_ICTPU Heat shock cognate 71 kDa protein - *Ictalurus punctatus* (Channel catfish) |
| hypothetical protein GSPATT00005209001 [*Paramecium tetraurelia* strain d4-2] |
| ISCU_HUMAN Iron-sulfur cluster assembly enzyme ISCU, mitochondrial precursor - *Homo sapiens* (Human) |
| ITPR_DROME Inositol 1,4,5-trisphosphate receptor - *Drosophila melanogaster* (Fruit fly) |
| LPQH_MYCTU Lipoprotein lpqH precursor - *Mycobacterium tuberculosis* |
| MANC_DICDI Alpha-mannosidase C precursor - *Dictyostelium discoideum* (Slime mold) |
| MATK_LOIPR Maturase K - *Loiseleuria procumbens* (Alpine azalea) (*Azalea procumbens*) |
| MU162_SCHPO Meiotically up-regulated gene 162 protein - *Schizosaccharomyces pombe* (Fission yeast) |
| NFYA_RAT Nuclear transcription factor Y subunit alpha - *Rattus norvegicus* (Rat) |
| NOL11_PONPY Nucleolar protein 11 - *Pongo pygmaeus* (Bornean orangutan) |
| O44231 *anthocidaris crassispina* (sea urchin). outer arm dynein light chain 1. |
| P41824 *aplysia californica* (california sea hare). y-box factor homolog (apy1). |
| P42578 *lymnaea stagnalis* (great pond snail). yolk ferritin precursor (ec 1.16.3.1). |
| PHLPP_RAT PH domain leucine-rich repeat protein phosphatase - *Rattus norvegicus* (Rat) |
| Q16960 *anthocidaris crassispina* (sea urchin). dynein intermediate chain 3, ciliary. |
| Q32sg8 *brachydanio rerio* (zebrafish) (danio rerio). sodium calcium exchanger 1h. |
| Q3hnk3 *mytilus edulis* (blue mussel). 60s ribosomal protein l13a. |
| Q3hri5 *mytilus edulis* (blue mussel). x-box binding protein 1 (fragment). |
| Q3kvl7 *codakia orbicularis*. 15 kda major gill protein. |
| Q3zlc7 *oreochromis mossambicus* (mozambique tilapia) (*tilapia mossambica*). selenoprotein w2a. |
| Q4i111 *gibberella zeae* (fusarium graminearum). hypothetical protein. |
| Q4kth3 *suberites domuncula* (sponge). l13. |
| Q4rel3 *tetraodon nigroviridis* (green puffer). chromosome 10 scaf15123, whole genome shotgun sequence. |
| Q4rf58 *tetraodon nigroviridis* (green puffer). chromosome 14 scaf15120, whole genome shotgun sequence. |
| Q4syh6 *tetraodon nigroviridis* (green puffer). chromosome undetermined scaf12061, whole genome shotgun sequence. |
| Q4szj2 *tetraodon nigroviridis* (green puffer). chromosome undetermined scaf11605, whole genome shotgun sequence. (fragment). |
| Q4tes1 *tetraodon nigroviridis* (green puffer). chromosome undetermined scaf5157, whole genome shotgun sequence. (fragment). |
| Q4v910 *brachydanio rerio* (zebrafish) (*danio rerio*). loc553269 protein (fragment). |
| Q4vbt4 *brachydanio rerio* (zebrafish) (*danio rerio*). translation elongation factor 1-gamma. |
| Q4w7i9 *nasutitermes takasagoensis*. hypotheical protein (fragment). |
| Q5i0s5 *xenopus tropicalis* (western clawed frog) (silurana tropicalis). c1ql1-prov protein (fragment). |
| Q5m9l3 *mus musculus* (mouse). ribosomal protein s21. |
| Q5tx21 *anopheles gambiae* str. pest. ensangp00000028140 (fragment). |
| Q5xj47 *brachydanio rerio* (zebrafish) (danio rerio). h1m protein (fragment). |
| Q5zk08 *gallus gallus* (chicken). hypothetical protein. |
| Q5zm92 *gallus gallus* (chicken). hypothetical protein. |
| Q65zx2 *borrelia garinii*. translation initiation factor 2. |
| Q66kq6 *xenopus laevis* (african clawed frog). mgc85528 protein. |
| Q6e2n2 *brachydanio rerio* (zebrafish) (danio rerio). transcriptional intermediary factor 1 alpha. |
| Q6gq33 *xenopus laevis* (african clawed frog). mgc80379 protein. |
| Q6j0s6 *branchiostoma belcheri* tsingtaunese. duf614 protein. |
| Q6nwf4 *brachydanio rerio* (zebrafish) (danio rerio). vacuolar protein sorting protein 25. |
| Q6ppc9 *haliotis rubra*. nadh dehydrogenase subunit 5. |
| Q6pti1 *modiolus americanus* (american horsemussel). atp synthase beta subunit (fragment). |
| Q6xi82 *drosophila yakuba* (fruit fly). similar to drosophila melanogaster cg13298 (fragment). |
| Q7q191 *anopheles gambiae* str. pest. ensangp00000013345 (fragment). |
| Q7qia8 *anopheles gambiae* str. pest. ensangp00000020311. |
| Q7qlc5 *anopheles gambiae* str. pest. ensangp00000001833. |
| Q7yzr8 *crassostrea gigas* (pacific oyster). ferritin gf2. |
| Q7zyn6 *xenopus laevis* (african clawed frog). loc398452 protein (fragment). |
| Q800v7 *brachydanio* *rerio* (zebrafish) (danio rerio). cardiac troponin c. |
| Q86ei2 *schistosoma japonicum* (blood fluke). clone zzd355 mrna sequence. |
| Q8jhv9 *xenopus* *laevis* (african clawed frog). iap-like protein (xeiap). |
| Q8mpm1 *lumbricus* *terrestris* (common earthworm). gelsolin-like protein. |
| Q8wp36 *suberites* *domuncula* (sponge). col protein. |
| Q91423 catfish. phosphoinositide-specific phospholipase c (fragment). |
| Q95w89 *branchiostoma* *belcheri* (amphoxius). 60s ribosomal protein l37a. |
| Q9cqm5 m 10, 11 days embryo whole body cdna, riken full-length enriched library, clone:2810417d24 product:hypothetical thioredoxin containing protein, full insert sequence (hypothetical protein txnl5) (10 day old male pancreas cdna, riken full-. |
| Q9i9e4 *pleurodeles* *waltlii* (iberian ribbed newt). putative nuclear movement protein pnudc. |
| RSHL3_HUMAN Radial spokehead-like protein 3 - *Homo* *sapiens* (Human) |
| TERT_YEAST Telomerase reverse transcriptase - *Saccharomyces* *cerevisiae* (Baker's yeast) |
| TETX_CLOTE Tetanus toxin precursor - *Clostridium* *tetani* |
| thioredoxin 2 [*Gallus* *gallus*] |
| Y1252_HAEIN Uncharacterized ABC transporter ATP-binding protein HI1252 - *Haemophilus* *influenzae* |
| Y1828_AQUAE Uncharacterized protein aq_1828 - *Aquifex* *aeolicus* |
| YCF1_SOLBU Putative membrane protein ycf1 - *Solanum* *bulbocastanum* (Wild potato) |
| YR570_MIMIV Uncharacterized protein R570 - *Acanthamoeba* *polyphaga* mimivirus (APMV) |
| YURN_BACSU Probable ABC transporter permease protein yurN - *Bacillus* *subtilis* |
| YX0F_CAEEL Uncharacterized protein C03B1.15 - *Caenorhabditis* *elegans* |

Table S7. Genes with higher expression in Rb mussels with high SOX content

| EST100; EST1014; EST1024; EST1029; EST1034; EST1038; EST1040; EST1043; EST1066; EST1075; EST1076; EST1081; EST1085; EST1092; EST1095; EST1130; EST115; EST116; EST1180; EST1216; EST1224; EST1230; EST1238; EST1241; EST1255; EST1278; EST1285; EST129; EST1298; EST1320; EST134; EST1343; EST135; EST1351; EST136; EST1362; EST1373; EST1382; EST1408; EST1468; EST1487; EST151; EST1511; EST1540; EST164; EST181; EST184; EST197; EST202; EST220; EST226; EST239; EST244; EST253; EST259; EST278; EST285; EST29; EST299; EST3; EST352; EST355; EST37; EST385; EST389; EST390; EST392; EST393; EST434; EST437; EST45; EST461; EST462; EST472; EST473; EST481; EST497; EST513; EST541; EST557; EST560; EST571; EST602; EST635; EST649; EST658; EST663; EST668; EST681; EST727; EST74; EST761; EST763; EST78; EST789; EST80; EST82; EST823; EST825; EST832; EST836; EST843; EST85; EST854; EST860; EST867; EST877; EST880; EST884; EST894; EST896; EST900; EST902; EST908; EST916; EST923; EST928; EST935; EST936; EST940; EST951; EST96; EST960; EST977; EST983; EST990 |
| --- |
| 17 sequences no_hits_found |
| ABCG5_RAT ATP-binding cassette sub-family G member 5 - *Rattus* *norvegicus* (Rat) |
| ACOD_CYPCA Acyl-CoA desaturase - *Cyprinus* *carpio* (Common carp) |
| CD97_BOVIN CD97 antigen precursor - *Bos* *taurus* (Bovine) |
| CDPK4_PLAYO Calcium-dependent protein kinase 4 - *Plasmodium* *yoelii* *yoelii* |
| CO1A1_CYNPY Collagen alpha-1(I) chain precursor - *Cynops* *pyrrhogaster* (Japanese common newt) |
| COAT4_MIMIV Probable capsid protein 4 - *Acanthamoeba* *polyphaga* mimivirus (APMV) |
| COX3_LUMTE Cytochrome c oxidase subunit 3 - *Lumbricus* *terrestris* (Common earthworm) |
| CRCB_PSEA7 Protein crcB homolog - *Pseudomonas* *aeruginosa* (strain PA7) |
| CS018_HUMAN Uncharacterized protein C19orf18 precursor - *Homo* *sapiens* (Human) |
| cytochrome c oxidase polypeptide VIb [*Botryotinia* *fuckeliana* |
| DAZP1_MOUSE DAZ-associated protein 1 - *Mus* *musculus* (Mouse) |
| DDX17_MOUSE Probable ATP-dependent RNA helicase DDX17 - *Mus* *musculus* (Mouse) |
| DFFA_HUMAN DNA fragmentation factor subunit alpha - *Homo* *sapiens* (Human) |
| DHGL_DROME Glucose dehydrogenase [acceptor] precursor - *Drosophila* *melanogaster* (Fruit fly) |
| diaphanous [*Aedes* *aegypti*] |
| DPO3B_CHLTR DNA polymerase III subunit beta - *Chlamydia* *trachomatis* |
| DPYL3_PONPY Protein dpy-19 homolog 3 - *Pongo* *pygmaeus* (Bornean orangutan) |
| ERF1X_ARATH Eukaryotic peptide chain release factor subunit 1-1 - *Arabidopsis* *thaliana* (Mouse-ear cress) |
| esterase/lipase hi0193 [*Plasmodium* *yoelii* *yoelii* str |
| FB92_ARATH F-box protein At1g78280 - *Arabidopsis* *thaliana* (Mouse-ear cress) |
| GBLP_NICPL Guanine nucleotide-binding protein subunit beta-like protein - *Nicotiana* *plumbaginifolia* (Leadwort-leaved tobacco) |
| GCSP_SCHPO Putative glycine dehydrogenase [decarboxylating], mitochondrial precursor - *Schizosaccharomyces* *pombe* (Fission yeast) |
| GO:0005524; F:ATP binding; IEA GO:0000166; F:nucleotide binding; IEA |
| GO:0005529; F:sugar binding; IEA |
| GO:0006950; P:response to stress; IEA |
| GO:0016021; C:integral to membrane; IEA GO:0015031; P:protein transport; IEA |
| GPA2_SCHPO Guanine nucleotide-binding protein alpha-2 subunit - *Schizosaccharomyces* *pombe* (Fission yeast) |
| GPDA_BACSU Glycerol-3-phosphate dehydrogenase [NAD(P)+] - *Bacillus* *subtilis* |
| GRN_HUMAN Granulins precursor - *Homo* *sapiens* (Human) |
| HNRPL_HUMAN Heterogeneous nuclear ribonucleoprotein L - *Homo* *sapiens* (Human) |
| HSP7C_ICTPU Heat shock cognate 71 kDa protein - *Ictalurus* *punctatus* (Channel catfish) |
| IAA3_ARATH Auxin-responsive protein IAA3 - *Arabidopsis* *thaliana* (Mouse-ear cress) |
| ITBX_DROME Integrin beta-PS precursor - *Drosophila* *melanogaster* (Fruit fly) |
| KTHY_HUMAN Thymidylate kinase - *Homo* *sapiens* (Human) |
| LICH_RAT Lysosomal acid lipase/cholesteryl ester hydrolase precursor - *Rattus* *norvegicus* (Rat) |
| MCAT_MOUSE Mitochondrial carnitine/acylcarnitine carrier protein - *Mus* *musculus* (Mouse) |
| MDM31_YEAST Mitochondrial distribution and morphology protein 31, mitochondrial precursor - *Saccharomyces* *cerevisiae* (Baker's yeast) |
| MEGF6_MOUSE Multiple epidermal growth factor-like domains 6 precursor - *Mus* *musculus* (Mouse) |
| MOEB_PORYE Probable molybdopterin biosynthesis protein moeB - *Porphyra* *yezoensis* |
| NDUA5_BOVIN NADH dehydrogenase [ubiquinone] 1 alpha subcomplex subunit 5 - *Bos* *taurus* (Bovine) |
| NOL11_PONPY Nucleolar protein 11 - *Pongo* *pygmaeus* (Bornean orangutan) |
| O14463 *schizosaccharomyces* *pombe* (fission yeast). thioredoxin (tr). |
| O44231 *anthocidaris* *crassispina* (sea urchin). outer arm dynein light chain 1. 6/2002 |
| O65639 *arabidopsis* *thaliana* (mouse-ear cress). glycine-rich protein. |
| O96789 *strongylocentrotus* *purpuratus* (purple sea urchin). nad(p) transhydrogenase (ec 1.6.1.1) (fragment). |
| ORF-2 [*Lactobacillus* *sakei*] |
| P2RX5_RAT P2X purinoceptor 5 - *Rattus* *norvegicus* (Rat) |
| P41824 *aplysia* *californica* (california sea hare). y-box factor homolog (apy1). |
| P42578 *lymnaea* *stagnalis* (great pond snail). yolk ferritin precursor (ec 1.16.3.1). |
| P80251 *mytilus* *edulis* (blue mussel). metallothionein 20-i isoforms a and b (mt-20-ia and mt-20-ib). |
| PGES2_DANRE Prostaglandin E synthase 2 - *Danio* r*e*rio (Zebrafish) (*Brachydanio* *rerio*) |
| POL1_RRVC RNA1 polyprotein - Raspberry ringspot virus (strain cherry) (RpRSV) |
| Q32sg8 *brachydanio* *rerio* (zebrafish) (*danio* *rerio*). sodium calcium exchanger 1h. |
| Q33604 *lamna* *nasus* (porbeagle) (*squalus* *nasus*). cytochrome b. |
| Q3uqz8 *mus* *musculus* (mouse). 12 days embryo spinal ganglion cdna, riken full-length enriched library, clone:d130065h16 product:heat shock 70kda protein 12a, full insert sequence. |
| Q4h3a4 *ciona* *intestinalis*. jagged protein (fragment). |
| Q4rhs1 *tetraodon* *nigroviridis* (green puffer). chromosome 8 scaf15044, whole genome shotgun sequence. |
| Q4s0q0 *tetraodon* *nigroviridis* (green puffer). chromosome 2 scaf14781, whole genome shotgun sequence. |
| Q4s2q2 *tetraodon* *nigroviridis* (green puffer). chromosome 17 scaf14760, whole genome shotgun sequence. |
| Q4szj2 *tetraodon* *nigroviridis* (green puffer). chromosome undetermined scaf11605, whole genome shotgun sequence. (fragment). |
| Q4x807 *plasmodium* *chabaudi*. hypothetical protein (fragment). |
| Q502k2 *brachydanio* *rerio* (zebrafish) (danio rerio). loc553453 protein (fragment). |
| Q5i0s5 *xenopus* *tropicalis* (western clawed frog) (silurana tropicalis). c1ql1-prov protein (fragment). |
| Q5m8z2 *xenopus* *tropicalis* (western clawed frog) (silurana tropicalis). hypothetical loc496649. |
| Q5tmp1 *anopheles* *gambiae* str. pest. ensangp00000028262. |
| Q6dbu2 *brachydanio* *rerio* (zebrafish) (danio rerio). hypothetical protein zgc:91844. |
| Q6ddm3 *xenopus* *laevis* (african clawed frog). pecr-prov protein. |
| Q6fhl2 *homo* *sapiens* (human). birc5 protein. |
| Q6jwu8 *brachydanio* *rerio* (zebrafish) (*danio* *rerio*). coatomer protein complex subunit beta 1. |
| Q6pbr1 *brachydanio* *rerio* (zebrafish) (*danio* *rerio*). loc402878 protein (fragment). |
| Q6pby4 *brachydanio* *rerio* (zebrafish) (*danio* *rerio*). hypothetical protein smfn. |
| Q6pdk0 *mus* *musculus* (mouse). hypothetical protein. |
| Q6pi45 *brachydanio* *rerio* (zebrafish) (*danio* *rerio*). similar to immunoglobulin (cd79a) binding protein 1. |
| Q6pti1 *modiolus* *americanus* (american horsemussel). atp synthase beta subunit (fragment). |
| Q70ph4 *crassostrea* *gigas* (pacific oyster). omega class glutathione s-transferase. |
| Q75c49 *ashbya* *gossypii* (yeast) (eremothecium gossypii). acr068wp. |
| Q7kse4 *drosophila* *melanogaster* (fruit fly). cg31240-pa (gh05443p). |
| Q7q7q5 *anopheles* *gambiae* str. pest. ensangp00000021588 (fragment). |
| Q7zvn5 *brachydanio* *rerio* (zebrafish) (*danio* *rerio*). signal recognition particle 54. |
| Q800v7 *brachydanio* *rerio* (zebrafish) (*danio* *rerio*). cardiac troponin c. |
| Q868z7 *strongylocentrotus* *purpuratus* (purple sea urchin). heat shock protein gp96. |
| Q8ita5 *aequipecten* *irradians* (bay scallop). ribosomal protein l9. |
| Q8itb8 *aequipecten* *irradians* (bay scallop). ribosomal protein l23a (fragment). |
| Q8itc2 *aequipecten* *irradians* (bay scallop). ribosomal protein l11. |
| Q8ta69 *crassostrea* *gigas* (pacific oyster). actin 2. |
| Q8tfa7 neocall*i*mastix *frontalis* (rumen fungus). adp/atp carrier. |
| Q94613 *mya* *arenaria*. 40s ribosomal protein s19. |
| Q9i9e4 *pleurodeles* *waltlii* (iberian ribbed newt). putative nuclear movement protein pnudc. |
| Q9u8w8 *tachypleus* *tridentatus* (japanese horseshoe crab). techylectin-5a. |
| Q9vf83 *drosophila* *melanogaster* (fruit fly). cg31301-pa (ld33178p). |
| Q9z0r9 *mus* *musculus* (mouse). delta-6 fatty acid desaturase (fatty acid desaturase 2) (12 days embryo whole body cdna, riken full-length enriched library, clone:e970008a17 product:fatty acid desaturase 2, full insert sequence). |
| RIR2_DROME Ribonucleoside-diphosphate reductase subunit M2 - *Drosophila* *melanogaster* (Fruit fly) |
| RL17_RAT 60S ribosomal protein L17 - *Rattus* *norvegicus* (Rat) |
| RL5_STYCL 60S ribosomal protein L5 - *Styela* *clava* (Sea squirt) |
| RNAS6_PAPHA Ribonuclease K6 precursor - *Papio* *hamadryas* (Hamadryas baboon) |
| RPN1_RAT Dolichyl-diphosphooligosaccharide--protein glycosyltransferase subunit 1 precursor - *Rattus* *norvegicus* (Rat) |
| RS21_MOUSE 40S ribosomal protein S21 - *Mus* *musculus* (Mouse) |
| RSBNL_XENTR Round spermatid basic protein 1-like - *Xenopus* *tropicalis* (Western clawed frog) (*Silurana* *tropicalis*) |
| SOBP_RAT Sine oculis-binding protein homolog - *Rattus* *norvegicus* (Rat) |
| SODM_PASMU Superoxide dismutase [Mn] - *Pasteurella* *multocida* |
| SP2M_BACSU Stage II sporulation protein M - *Bacillus* *subtilis* |
| STUB_DROME Serine proteinase stubble - *Drosophila* *melanogaster* (Fruit fly) |
| SYH_CYTH3 Histidyl-tRNA synthetase - *Cytophaga* *hutchinsonii* (strain ATCC 33406 / NCIMB 9469) |
| SYL_FERNB Leucyl-tRNA synthetase-*Fervidobacterium* *nodosum* (strain ATCC 35602/DSM 5306/Rt17-B1) |
| TBB1_BRUPA Tubulin beta-1 chain - *Brugia* *pahangi* (Filarial nematode worm) |
| TRPP_BACSU Probable tryptophan transport protein - *Bacillus* *subtilis* |
| TRUA_MYCMS tRNA pseudouridine synthase A - *Mycoplasma* *mycoides* subsp. mycoides SC |
| U384_RAT UPF0384 protein CGI-117 homolog - *Rattus* *norvegicus* (Rat) |
| UBP21_MOUSE Ubiquitin carboxyl-terminal hydrolase 21 - *Mus* *musculus* (Mouse) |
| VSH_MUMPK Small hydrophobic protein - Mumps virus (strain Kilham) |
| YCF2_PHYPA Protein ycf2 - *Physcomitrella* *patens* (Moss) |
| YCF2_SPIOL Protein ycf2 - *Spinacia* *oleracea* (Spinach) |
| YP23_CAEEL Uncharacterized protein AH6.3 - *Caenorhabditis* *elegans* |
| YR541_MIMIV Putative BTB/POZ domain-containing protein R541-*Acanthamoeba* *polyphaga* mimivirus (APMV) |
| YR837_MIMIV Putative ankyrin repeat protein R837 - *Acanthamoeba* *polyphaga* mimivirus (APMV) |
| YSM6_CAEEL Uncharacterized protein F52C9.6 - *Caenorhabditis* *elegans* |

Table S8. Genes with higher expression in Rb mussels with low SOX content

| EST1010; EST1015; EST102; EST1022; EST1033; EST1042; EST1048; EST1053; EST1070; EST1073; EST1080; EST1122; EST1129; EST1146; EST1172; EST1173; EST1182; EST1185; EST1189; EST1190; EST1213; EST1249; EST1250; EST1259; EST1269; EST1270; EST1271; EST1292; EST1294; EST1307; EST1317; EST1324; EST1327; EST1329; EST1361; EST1377; EST1379; EST1392; EST1394; EST1398; EST1409; EST1413; EST1415; EST1446; EST1474; EST1498; EST1520; EST1525; EST1529; EST1531; EST1548; EST1557; EST161; EST174; EST185; EST191; EST281; EST286; EST291; EST31; EST317; EST32; EST323; EST345; EST354; EST360; EST384; EST391; EST405; EST438; EST440; EST47; EST483; EST490; EST52; EST548; EST552; EST599; EST604; EST610; EST619; EST623; EST629; EST630; EST633; EST643; EST657; EST664; EST673; EST675; EST68; EST680; EST694; EST695; EST699; EST705; EST706; EST716; EST724; EST732; EST733; EST742; EST743; EST760; EST764; EST772; EST773; EST774; EST776; EST787; EST793; EST798; EST802; EST813; EST838; EST846; EST848; EST897; EST914; EST918; EST966; EST972; EST985; EST993 |
| --- |
| 15 sequences no_hits_found |
| 209L2_MACMU CD209 antigen-like protein 2 - *Macaca* *mulatta* (Rhesus macaque) |
| AAS_KLEP7 Bifunctional protein aas [Includes: 2-acylglycerophosphoethanolamine acyltransferase - *Klebsiella* *pneumoniae* subsp. *pneumoniae* (strain ATCC 700721 / MGH 78578) |
| ACTP2_CYMEC Echotoxin-2 precursor - *Cymatium* *echo* (Giant triton) (Monoplex echo) |
| ANTA_HYDMA Antistasin precursor - *Hydra* *magnipapillata* (Hydra) |
| apical endosomal glycoprotein [*Strongylocentrotus* *purpuratus*] |
| ARI3A_XENLA AT-rich interactive domain-containing protein 3A - *Xenopus* *laevis* (African clawed frog) |
| ATPK_DROME Putative ATP synthase subunit f, mitochondrial - *Drosophila* *melanogaster* (Fruit fly) |
| ATX1_PLAFA Probable cation-transporting ATPase 1 - *Plasmodium* *falciparum* |
| BSC6_YEAST Bypass of stop codon protein 6 - *Saccharomyces* *cerevisiae* (Baker's yeast) |
| CA151_MOUSE UPF0327 protein C1orf151 homolog - *Mus* *musculus* (Mouse) |
| CHS1_CRYNV Chitin synthase 1 - *Cryptococcus* *neoformans* var. *grubii* (Filobasidiella neoformans var. grubii) |
| COX1_MYTED Cytochrome c oxidase subunit 1 - *Mytilus* *edulis* (Blue mussel) |
| CP4E2_DROME Cytochrome P450 4e2 - *Drosophila* *melanogaster* (Fruit fly) |
| CTNA_DICDI Countin-1 precursor - *Dictyostelium* *discoideum* (Slime mold) |
| CX6A1_MOUSE Cytochrome c oxidase polypeptide VIa-liver, mitochondrial precursor-*Mus* *musculus* (Mouse) |
| cytochrome bd-type quinol oxidase subunit 2 [*Exiguobacterium* *sibiricum* 255-15] |
| DAF19_CAEEL RFX-like transcription factor daf-19 - *Caenorhabditis* *elegans* |
| DBF20_YEAST Serine/threonine-protein kinase DBF20 - *Saccharomyces* *cerevisiae* (Baker's yeast) |
| DCPS_HUMAN Scavenger mRNA-decapping enzyme DcpS - *Homo* *sapiens* (Human) |
| DYHC_ONCMY Dynein heavy chain - *Oncorhynchus* *mykiss* (Rainbow trout) (*Salmo* *gairdneri*) |
| EAA3_MOUSE Excitatory amino acid transporter 3 - *Mus* *musculus* (Mouse) |
| ETV6_MOUSE Transcription factor ETV6 - *Mus* *musculus* (Mouse) |
| FTSK_BORBU DNA translocase ftsK - *Borrelia* *burgdorferi* (Lyme disease spirochete) |
| GMPPB_DANRE Mannose-1-phosphate guanyltransferase beta - *Danio* *rerio* (Zebrafish) (*Brachydanio* *rerio*) |
| GO:0000151; C:ubiquitin ligase complex; IEA GO:0006355; P:regulation of transcription, DNA-dependent; IEA |
| GO:0004364; F:glutathione transferase activity; NAS |
| GO:0004563; F:beta-N-acetylhexosaminidase activity; IEA GO:0005975; P:carbohydrate metabolism; IEA |
| GO:0005488; F:binding; IEA |
| GO:0005694; C:chromosome; IEA GO:0006281; P:DNA repair; IEA |
| GO:0016021; C:integral to membrane; IEA GO:0005488; F:binding; IEA |
| GO:0019028; C:viral capsid; IEA |
| GPDA_LACRF Glycerol-3-phosphate dehydrogenase [NAD(P)+] - *Lactobacillus* *reuteri* (strain ATCC 23272 / DSM 20016 / F275) |
| GSPN_KLEPN General secretion pathway protein N - *Klebsiella* *pneumoniae* |
| H90A1_DANRE Heat shock protein HSP 90-alpha 1 - *Danio* *rerio* (Zebrafish) (*Brachydanio* *rerio*) |
| HIP_MYTED Heavy metal-binding protein HIP - *Mytilus* *edulis* (Blue mussel) |
| IBPL1_MOUSE Insulin-like growth factor-binding protein-like 1 precursor - *Mus* *musculus* (Mouse) |
| ISPZ_AERHH Probable intracellular septation protein - *Aeromonas* *hydrophila* subsp. *hydrophila* (strain ATCC 7966 / NCIB 9240) |
| KHDR2_HUMAN KH domain-containing, RNA-binding, signal transduction-associated protein 2 - *Homo* *sapiens* (Human) |
| KHDR3_MOUSE KH domain-containing, RNA-binding, signal transduction-associated protein 3 - *Mus* *musculus* (Mouse) |
| KNG1_MOUSE Kininogen-1 precursor [Contains: Kininogen-1 heavy chain; Bradykinin; Kininogen-1 light chain] - *Mus* *musculus* (Mouse) |
| LEF2_NPVOP Late expression factor 2 - *Orgyia* *pseudotsugata* |
| LIMK2_HUMAN LIM domain kinase 2 - *Homo* *sapiens* (Human) |
| LRP8_HUMAN Low-density lipoprotein receptor-related protein 8 precursor - *Homo* *sapiens* (Human) |
| *Marsilea* *quadrifolia* mRNA for NAD+-dependent glyceraldehyde-3-phosphate dehydrogenase |
| MDMC_STRMY O-methyltransferase mdmC - *Streptomyces* *mycarofaciens* |
| MGR2_XENTR Protein MGR2 homolog - *Xenopus* *tropicalis* (Western clawed frog) (*Silurana* *tropicalis*) |
| MID1_YEAST Stretch-activated cation channel MID1 precursor - *Saccharomyces* *cerevisiae* (Baker's yeast) |
| MOT4_HUMAN Monocarboxylate transporter 4 - *Homo* *sapiens* (Human) |
| MTB2_BACSU Modification methylase BglII - *Bacillus* *subtilis* |
| MU162_SCHPO Meiotically up-regulated gene 162 protein - *Schizosaccharomyces* *pombe* (Fission yeast) |
| MVP_DISOM Major vault protein - *Discopyge* *ommata* (Electric ray) |
| NASP_RAT Nuclear autoantigenic sperm protein - *Rattus* *norvegicus* (Rat) |
| NCAM2_MOUSE Neural cell adhesion molecule 2 precursor - *Mus* *musculus* (Mouse) |
| NCTR1_MACFA Natural cytotoxicity triggering receptor 1 precursor - *Macaca* *fascicularis* (Crab eating macaque) (Cynomolgus monkey) |
| NST1_PICGU Stress response protein NST1 - *Pichia* *guilliermondii* (Yeast) (*Candida* *guilliermondii*) |
| NU2M_MYTED NADH-ubiquinone oxidoreductase chain 2 - *Mytilus* *edulis* (Blue mussel) |
| NU4LM_MYXGL NADH-ubiquinone oxidoreductase chain 4L - *Myxine* *glutinosa* (Atlantic hagfish) |
| NUDC_RAT Nuclear migration protein nudC - *Rattus* *norvegicus* (Rat) |
| OPDA_ECOLI Oligopeptidase A - *Escherichia* *coli* (strain K12) |
| P08991 *strongylocentrotus* *purpuratus* (purple sea urchin). histone h2a variant (fragment). |
| PDE3B_RAT cGMP-inhibited 3',5'-cyclic phosphodiesterase B - *Rattus* *norvegicus* (Rat) |
| PK2L2_MOUSE Polycystic kidney disease 2-like 2 protein - *Mus* *musculus* (Mouse) |
| PPK25_SCHPO Serine/threonine-protein kinase ppk25 - *Schizosaccharomyces* *pombe* (Fission yeast) |
| predicted protein [*Nematostella* *vectensis* |
| Q17286 *barbatia* *virescens*. hemoglobin (heterodimeric). |
| Q28290 *canis* *familiaris* (dog). cell adhesion molecule precursor (fragment). |
| Q30hu9 *crassostrea* *virginica* (eastern oyster). serine protease inhibitor 1. |
| Q3s337 *mytilus* *edulis* (blue mussel). collagen protein (fragment). |
| Q3zm03 *xenopus* *laevis* (african clawed frog). 67kd laminin receptor. |
| Q4h451 *crassostrea* *gigas* (pacific oyster). ribosomal protein s5. |
| Q4pm17 *ixodes* *scapularis* (black-legged tick) (deer tick). ribosomal protein l35a. |
| Q4rmm1 *tetraodon* *nigroviridis* (green puffer). chromosome 10 scaf15019, whole genome shotgun sequence. |
| Q4ruz5 *tetraodon* *nigroviridis* (green puffer). chromosome 12 scaf14993, whole genome shotgun sequence. |
| Q4te05 *tetraodon* *nigroviridis* (green puffer). chromosome undetermined scaf5903, whole genome shotgun sequence. |
| Q5f1m8 *mytilus* *galloprovincialis* (mediterranean mussel). nadh dehydrogenase subunit 4. |
| Q5rgn5 *brachydanio* *rerio* (zebrafish) (danio rerio). novel protein similar to human and mouse complement component 1, q subcomponent-like 1 (c1ql1) (fragment). |
| Q5u3u7 *brachydanio* *rerio* (zebrafish) (*danio* *rerio*). zgc:101615. |
| Q5xua5 *toxoptera* *citricida* (brown citrus aphid). probable transport protein sec61 alpha subunit. |
| Q5zkx0 *gallus* *gallus* (chicken). hypothetical protein. |
| Q613d1 *caenorhabditis* *briggsae*. hypothetical protein cbg16448 (fragment). |
| Q67uu9 *oryza* *sativa* (japonica cultivar-group). putative keratin associated protein. |
| Q68er8 *xenopus* *tropicalis* (western clawed frog) (*silurana* *tropicalis*). mgc89305 protein. |
| Q6bd10 *mytilus* *galloprovincialis* (mediterranean mussel). elongation factor 1 alpha. |
| Q6ddc1 *xenopus* *tropicalis* (western clawed frog) (*silurana* *tropicalis*). psmd7 protein (fragment). |
| Q6ddl4 *xenopus* *laevis* (african clawed frog). loc398446 protein. |
| Q6iqj2 *brachydanio* *rerio* (zebrafish) (*danio* *rerio*). zgc:55461 protein. |
| Q6pc91 *brachydanio* rerio (zebrafish) (danio rerio). basic transcription factor 3-like 4. |
| Q6psl2 *aplysia* *brasiliana* (mottled sea hare). temptin (fragment). |
| Q6qr01 *chiromantes* *haematocheir*. hsp-90. |
| Q6wn55 *branchiostoma* *belcheri* tsingtaunese. adenosylhomocysteinase. |
| Q75r50 *lymnaea* *stagnalis* (great pond snail). dec-3. |
| Q7kr73 *drosophila* *melanogaster* (fruit fly). cg18076-ph, isoform h. |
| Q7pqg7 *anopheles* *gambiae* str. pest. ensangp00000011832. |
| Q7pyg5 *anopheles* *gambiae* str. pest. ensangp00000018356 (fragment |
| Q7q7z4 *anopheles* *gambiae* str. pest. ensangp00000022226 (fragment). |
| Q7q8r0 *anopheles* *gambiae* str. pest. ensangp00000017984 (fragment). |
| Q7zwt6 *xenopus* *laevis* (african clawed frog). tcerg1 protein (fragment). |
| Q86lp9 *branchiostoma* *belcheri* (amphoxius). hypothetical gaba(a) receptor-associated protein like-2. |
| Q86pl3 *perinereis* *aibuhitensis* (clamworm). 40s ribosomal protein. |
| Q8bfq8 m bc023835 protein (10 days neonate cerebellum cdna, riken full-length enriched library, clone:b930098g02 product:hypothetical thij/pfpi family containing protein, full insert sequence) (12 days embryo eyeball cdna, riken full-length enr. |
| Q8bmr6 *mus* *musculus* (mouse). 0 day neonate head cdna, riken full-length enriched library, clone:4833440h06 product:procollagen, type iii, alpha 1, full insert sequence. (fragment). |
| Q8cdt7 *mus* *musculus* (mouse). protein c10orf107 homolog. |
| Q8is80 *euprymna* *scolopes*. 60s acidic ribosomal protein (fragment). |
| Q8itb7 *aequipecten* *irradians* (bay scallop). ribosomal protein s4 (fragment). |
| Q8n5l1 *homo* *sapiens* (human). emilin2 protein (fragment). |
| Q8ni36 *homo* *sapiens* (human). wd-repeat protein 36 (t-cell activation wd repeat protein) (ta-wdrp). |
| Q8tup8 *methanosarcina* *acetivorans*. dynein heavy chain. |
| Q8ws60 *branchiostoma* *floridae* (florida lancelet) (amphioxus). endonuclease/reverse transcriptase. |
| Q94760 *strongylocentrotus* *purpuratus* (purple sea urchin). mitochondrial atp synthase alpha subunit precursor. |
| Q95nn1 *tribolium* *castaneum* (red flour beetle). tryptophan oxygenase. |
| Q99jc0 *rattus* *norvegicus* (rat). rrna promoter binding protein. |
| Q9czj2 *mus* *musculus* (mouse). heat shock 70 kda protein 12b. |
| Q9grj2 *lumbricus* *rubellus* (humus earthworm). actin related protein 2/3 protein complex subunit p16. |
| Q9lii5 *arabidopsis* *thaliana* (mouse-ear cress). *arabidopsis* *thaliana* genomic dna, chromosome 3, tac clone: k24m9. |
| Q9u5r6 *entodinium* *caudatum*. putative ribosomal protein (fragment). |
| Q9y092 *drosophila* *melanogaster* (fruit fly). cg8604-pa (amphiphysin) (ld19810p). |
| RDH13_HUMAN Retinol dehydrogenase 13 - *Homo* *sapiens* (Human) |
| RL8_DROME 60S ribosomal protein L8 - *Drosophila* *melanogaster* (Fruit fly) |
| RR3_EPIVI Plastid 30S ribosomal protein S3 - *Epifagus* *virginiana* (Beechdrops) |
| RS14_DROME 40S ribosomal protein S14 - *Drosophila* *melanogaster* (Fruit fly) |
| RS4_DROME 40S ribosomal protein S4 - *Drosophila* *melanogaster* (Fruit fly) |
| RS4_IXOSC 40S ribosomal protein S4 - *Ixodes* *scapularis* (Black-legged tick) (Deer tick) |
| RSMF_SHEON Ribosomal RNA small subunit methyltransferase F - *Shewanella* *oneidensis* |
| S230_PLAFO Transmission-blocking target antigen S230 precursor - *Plasmodium* *falciparum* (isolate NF54) |
| S5A2M_BOVIN Steroid 5-alpha-reductase 2-like 2 protein - *Bos* *taurus* (Bovine) |
| SAHH_CAEEL Adenosylhomocysteinase - *Caenorhabditis* *elegans* |
| SFTPA_RAT Pulmonary surfactant-associated protein A precursor - *Rattus* *norvegicus* (Rat) |
| SRS2_YEAST ATP-dependent DNA helicase SRS2 - *Saccharomyces* *cerevisiae* (Baker's yeast) |
| SYF1_DICDI Pre-mRNA-splicing factor SYF1 - *Dictyostelium* *discoideum* (Slime mold) |
| TATB_NITEU Sec-independent protein translocase protein tatB homolog - *Nitrosomonas* *europaea* |
| TBAT_ONCMY Tubulin alpha chain, testis-specific - *Oncorhynchus* *mykiss* (Rainbow trout) (*Salmo* *gairdneri*) |
| TCTP_BRABE Translationally-controlled tumor protein - *Branchiostoma* *belcheri* (Amphioxus) |
| TMBI4_HUMAN Transmembrane BAX inhibitor motif-containing protein 4 - *Homo* *sapiens* (Human) |
| transmembrane BAX inhibitor motif containing 4 [*Homo* *sapiens*] |
| UNC5D_MOUSE Netrin receptor UNC5D precursor - *Mus* *musculus* (Mouse) |
| Vacuolar H ATPase family member (vha-17) [*Caenorhabditis* *elegans*] |
| VP26B_PONPY Vacuolar protein sorting-associated protein 26B - *Pongo* *pygmaeus* (Bornean orangutan) |
| VPS52_SCHPO Vacuolar protein sorting-associated protein 52 - *Schizosaccharomyces* *pombe* (Fission yeast) |
| Y114_CHLMU Uncharacterized protein TC_0114 - *Chlamydia* *muridarum* |
| Y539_AQUAE Uncharacterized protein aq_539 - *Aquifex* *aeolicus* |
| Y816_AERPE UPF0130 protein APE_0816 - *Aeropyrum* *pernix* |
| YCF2_MORIN Protein ycf2 - *Morus* *indica* (Mulberry) |
| YD387_YEAST Probable metabolite transport protein YDR387C - *Saccharomyces* *cerevisiae* (Baker's yeast) |
| YL769_MIMIV Putative ankyrin repeat protein L769 - Acant*h*amoeba *polyphaga* mimivirus (APMV) |
| YOR6_TTV1K Uncharacterized 8.9 kDa protein - *Thermoproteus* *tenax* virus 1 (strain KRA1) (TTV1) |
| ZDHC6_DICDI Putative ZDHHC-type palmitoyltransferase 6 - *Dictyostelium* *discoideum* (Slime mold) |
| ZMYM2_PONPY MYM-type zinc finger protein 2 - *Pongo* *pygmaeus* (Bornean orangutan) |

Table S9. Genes with higher expression in Rb mussels with high MOX content

| EST100; EST1001; EST1006; EST1020; EST103; EST1031; EST1040; EST1063; EST1067; EST1075; EST1081; EST1091; EST1118; EST1139; EST1154; EST1157; EST1160; EST1196; EST1218; EST1230; EST1235; EST1248; EST129; EST1298; EST130; EST1304; EST1323; EST1326; EST1332; EST134; EST1343; EST135; EST136; EST1362; EST1363; EST1382; EST1390; EST1434; EST1484; EST1487; EST1505; EST1511; EST152; EST1539; EST164; EST170; EST171; EST182; EST192; EST202; EST219; EST233; EST239; EST242; EST253; EST258; EST265; EST295; EST310; EST313; EST322; EST348; EST352; EST355; EST36; EST367; EST37; EST371; EST389; EST390; EST392; EST398; EST4; EST411; EST424; EST428; EST445; EST461; EST487; EST493; EST505; EST51; EST513; EST524; EST54; EST543; EST555; EST560; EST571; EST573; EST6; EST60; EST602; EST605; EST635; EST641; EST658; EST659; EST668; EST727; EST739; EST761; EST763; EST78; EST789; EST796; EST80; EST817; EST82; EST821; EST825; EST832; EST839; EST843; EST844; EST849; EST854; EST884; EST888; EST889; EST893; EST894; EST902; EST928; EST929; EST955; EST960; EST969; EST974; EST975; EST976; EST977; EST98; EST989; EST992; EST998 |
| --- |
| 22 sequences no_hits_found |
| ACOD_CYPCA Acyl-CoA desaturase - *Cyprinus* *carpio* (Common carp) |
| ACTP2_CYMEC Echotoxin-2 precursor - *Cymatium* *echo* (Giant triton) (Monoplex echo) |
| ANT3_SHEEP Antithrombin-III precursor - *Ovis* *aries* (Sheep) |
| ANT3_SHEEP Antithrombin-III precursor - *Ovis* *aries* (Sheep) |
| ANX11_BOVIN Annexin A11 - *Bos* *taurus* (Bovine) |
| APAH_METFK Bis(5'-nucleosyl)-tetraphosphatase, symmetrical - *Methylobacillus* *flagellatus* (strain KT / ATCC 51484 / DSM 6875) |
| APJ_XENTR Apelin receptor - *Xenopus* *tropicalis* (Western clawed frog) (*Silurana* *tropicalis*) |
| ARY_ANTPE Arylphorin precursor - *Antheraea* *pernyi* (Chinese oak silk moth) |
| CANB1_RAT Calcineurin subunit B isoform 1 - *Rattus* *norvegicus* (Rat) |
| CCCP_DROYA Circadian clock-controlled protein precursor - *Drosophila* *yakuba* (Fruit fly) |
| CD97_BOVIN CD97 antigen precursor - *Bos* *taurus* (Bovine) |
| CO1A2_CHICK Collagen alpha-2(I) chain precursor - *Gallus* *gallus* (Chicken) |
| COAT4_MIMIV Probable capsid protein 4 - *Acanthamoeba* *polyphaga* mimivirus (APMV) |
| COIA1_MOUSE Collagen alpha-1(XVIII) chain precursor [Contains: Endostatin] - *Mus* *musculus* (Mouse) |
| COX1_LUMTE Cytochrome c oxidase subunit 1 - *Lumbricus* *terrestris* (Common earthworm) |
| COX41_BOVIN Cytochrome c oxidase subunit 4 isoform 1, mitochondrial precursor - *Bos* *taurus* (Bovine) |
| CQ061_BUFBG UPF0451 protein C17orf61 homolog precursor - *Bufo* *bufo* *gargarizans* (Asian toad) |
| CS018_HUMAN Uncharacterized protein C19orf18 precursor - *Homo* *sapiens* (Human) |
| CYAA_DICDI Adenylate cyclase, aggregation specific - *Dictyostelium* *discoideum* (Slime mold) |
| DAZP1_XENLA DAZ-associated protein 1 - *Xenopus* *laevis* (African clawed frog) |
| DHGL_DROME Glucose dehydrogenase [acceptor] precursor - *Drosophila* *melanogaster* (Fruit fly) |
| diaphanous [*Aedes* *aegypti*] |
| Dynein light chain 1, cytoplasmic [*Brugia* *malayi*] |
| EAF7_YEAST Chromatin modification-related protein EAF7 - *Saccharomyces* *cerevisiae* (Baker's yeast) |
| EF1A_BOMMO Elongation factor 1-alpha - *Bombyx* *mori* (Silk moth) |
| ERF1X_ARATH Eukaryotic peptide chain release factor subunit 1-1 - *Arabidopsis* *thaliana* (Mouse-ear cress) |
| FLHB_BUCBP Flagellar biosynthetic protein flhB - *Buchnera* *aphidicola* subsp. *Baizongia* *pistaciae* |
| GBLP_NICPL Guanine nucleotide-binding protein subunit beta-like protein - *Nicotiana* *plumbaginifolia* (Leadwort-leaved tobacco) |
| GGT1_RAT Gamma-glutamyltranspeptidase 1 precursor - *Rattus* *norvegicus* (Rat) |
| GIDB_MYCS5 Methyltransferase gidB - *Mycoplasma* *synoviae* (strain 53) |
| GO:0005488; F:binding; IEA |
| GO:0005529; F:sugar binding; IEA |
| GO:0015934; C:large ribosomal subunit; IEA GO:0006412; P:protein biosynthesis; IEA |
| GO:0016021; C:integral to membrane; IEA GO:0015031; P:protein transport; IEA |
| GRN_HUMAN Granulins precursor - *Homo* *sapiens* (Human) |
| HMGB2_CHICK High mobility group protein B2 - *Gallus* *gallus* (Chicken) |
| HNRPL_HUMAN Heterogeneous nuclear ribonucleoprotein L - *Homo* *sapiens* (Human) |
| HNRPR_HUMAN Heterogeneous nuclear ribonucleoprotein R - *Homo* *sapiens* (Human) |
| HYPD_ECOLI Hydrogenase isoenzymes formation protein hypD - *Escherichia* *coli* (strain K12) |
| hypothetical protein OsI_028693 [*Oryza* *sativa* (indica cultivar-group)] |
| hypothetical protein PFL1535w [*Plasmodium* *falciparum* 3D7] |
| IAA3_ARATH Auxin-responsive protein IAA3 - *Arabidopsis* *thaliana* (Mouse-ear cress) |
| IMB_DROME Importin subunit beta - *Drosophila* *melanogaster* (Fruit fly) |
| interferon-inducible GTPase_a [*Salmo* *salar*] |
| KTHY_HUMAN Thymidylate kinase - *Homo* *sapiens* (Human) |
| LEU3_BUCUN 3-isopropylmalate dehydrogenase - *Buchnera* *aphidicola* subsp. *Uroleucon* *sonchi* |
| LPE10_CANGA Inner membrane magnesium transporter LPE10, mitochondrial precursor - *Candida* *glabrata* (Yeast) (Torulopsis glabrata) |
| MCAT_MOUSE Mitochondrial carnitine/acylcarnitine carrier protein - *Mus* *musculus* (Mouse) |
| MDM31_YEAST Mitochondrial distribution and morphology protein 31, mitochondrial precursor - *Saccharomyces* *cerevisiae* (Baker's yeast) |
| MUC1_XENLA Integumentary mucin C.1 - *Xenopus* *laevis* (African clawed frog) |
| NADH dehydrogenase subunit 2 [*Mytilus* *galloprovincialis*] |
| NCAN_RAT Neurocan core protein precursor - *Rattus* *norvegicus* (Rat) |
| NOL11_PONPY Nucleolar protein 11 - Pongo pygmaeus (*Bornean* *orangutan*) |
| NU4M_APILI NADH-ubiquinone oxidoreductase chain 4 - *Apis* *mellifera* *ligustica* (Common honeybee) |
| NUD1_YEAST Protein NUD1 - *Saccharomyces* *cerevisiae* (Baker's yeast) |
| O14463 *schizosaccharomyces* *pombe* (fission yeast). thioredoxin (tr). |
| O42468 *tetraodon* *fluviatilis* (puffer fish). pp2a inhibitor. |
| O44231 *anthocidaris* *crassispina* (sea urchin). outer arm dynein light chain 1. |
| P41824 *aplysia* *californica* (california sea hare). y-box factor homolog (apy1). |
| P42578 *lymnaea* *stagnalis* (great pond snail). yolk ferritin precursor (ec 1.16.3.1). |
| P51544 *nordotis* *madaka* (giant abalone). arginine kinase (ec 2.7.3.3) (ak). |
| P56974 *mus* *musculus* (mouse). pro-neuregulin-2, membrane-bound isoform precursor (pro-nrg2) [contains: neuregulin-2 (nrg-2) (divergent of neuregulin 1) (don-1)]. |
| PE2R2_CANFA Prostaglandin E2 receptor EP2 subtype - *Canis* *familiaris* (Dog) |
| PF2L_PLAFP PPF2L antigen - *Plasmodium* *falciparum* (isolate Palo Alto / Uganda) |
| PGES2_DANRE Prostaglandin E synthase 2 - *Danio* *rerio* (Zebrafish) (*Brachydanio* *rerio*) |
| phospholipase A2, group IVB [*Bos* *taurus*] |
| PLMN_MOUSE Plasminogen precursor - *Mus* *musculus* (Mouse) |
| PPAX_BACP2 Pyrophosphatase ppaX - *Bacillus* *pumilus* (strain SAFR-032) |
| PPCK_DROME Phosphoenolpyruvate carboxykinase [GTP] - *Drosophila* *melanogaster* (Fruit fly) |
| PPIB_RAT Peptidyl-prolyl cis-trans isomerase B precursor - *Rattus* *norvegicus* (Rat) |
| predicted protein [*Nematostella* *vectensis*] |
| programmed cell death [*Culex* *pipiens* *quinquefasciatus*] |
| protein kinase [*Plasmodium* *berghei* strain ANKA] |
| PXDC1_MOUSE Plexin domain-containing protein 1 precursor - *Mus* *musculus* (Mouse) |
| Q27123 *urechis* *caupo* (innkeeper worm) (spoonworm). cytochrome c oxidase subunit iv. |
| Q32sg8 *brachydanio* *rerio* (zebrafish) (danio rerio). sodium calcium exchanger 1h. |
| Q33604 *lamna* *nasus* (porbeagle) (squalus nasus). cytochrome b. |
| Q4h352 *ciona* *intestinalis*. ci-nf45 protein. |
| Q4pm50 *ixodes* *scapularis* (black-legged tick) (deer tick). nadh dehydrogenase 1 alpha subcomplex-like. |
| Q4rf58 *tetraodon* *nigroviridis* (green puffer). chromosome 14 scaf15120, whole genome shotgun sequence. |
| Q4s2q2 *tetraodon* *nigroviridis* (green puffer). chromosome 17 scaf14760, whole genome shotgun sequence. |
| Q4sas9 *tetraodon* *nigroviridis* (green puffer). chromosome 3 scaf14679, whole genome shotgun sequence. |
| Q4v910 *brachydanio* *rerio* (zebrafish) (*danio* *rerio*). loc553269 protein (fragment). |
| Q503l9 *brachydanio* *rerio* (zebrafish) (*danio* *rerio*). hypothetical protein zgc:110449. |
| Q542v3 m 8 days embryo whole body cdna, riken full-length enriched library, clone:5730499p16 product:splicing factor, arginine/serine-rich 4 (srp75), full insert sequence (activated spleen cdna, riken full- length enriched library, clone:f83011 |
| Q5bvt5 *schistosoma* *japonicum* (blood fluke). sjchgc07766 protein (fragment). |
| Q5i0s5 *xenopus* *tropicalis* (western clawed frog) (silurana tropicalis). c1ql1-prov protein (fragment). |
| Q5m8z1 *xenopus* *tropicalis* (western clawed frog) (*silurana* *tropicalis*). loc496650 protein (fragment). |
| Q5rgn5 *brachydanio* *rerio* (zebrafish) (*danio* *rerio*). novel protein similar to human and mouse complement component 1, q subcomponent-like 1 (c1ql1) (fragment). |
| Q5tmp1 *anopheles* *gambiae* str. pest. ensangp00000028262. |
| Q5ttj1 *anopheles* *gambiae* str. pest. ensangp00000028072 (fragment). |
| Q5xj47 *brachydanio* *rerio* (zebrafish) (*danio* *rerio*). h1m protein (fragment). |
| Q5ya31 *urechis* *caupo* (innkeeper worm) (spoonworm). cytochrome oxidase subunit iii. |
| Q6axk1 *brachydanio* *rerio* (zebrafish) (*danio* *rerio*). zgc:100952. |
| Q6f6a1 *oryzias* *latipes* (medaka fish) (japanese ricefish). cathepsin l. |
| Q6gq33 *xenopus* *laevis* (african clawed frog). mgc80379 protein. |
| Q6iah9 *homo* *sapiens* (human). loc56901 protein. |
| Q6j0s6 *branchiostoma* *belcheri* tsingtaunese. duf614 protein. |
| Q6nyv9 *brachydanio* *rerio* (zebrafish) (*danio rerio*). senescence downregulated leo1-like. |
| Q6pbr1 *brachydanio* *rerio* (zebrafish) (*danio rerio*). loc402878 protein (fragment). |
| Q70ph4 *crassostrea gigas* (pacific oyster). omega class glutathione s-transferase. |
| Q7qia8 *anopheles gambiae* str. pest. ensangp00000020311. |
| Q7qlc5 *anopheles gambiae* str. pest. ensangp00000001833. |
| Q7zyn6 *xenopus laevis* (african clawed frog). loc398452 protein (fragment). |
| Q8cmb1 *vibrio vulnificus*. hypothetical protein (chlorobium tepidum orf122 like protein). |
| Q8ita5 *aequipecten irradians* (bay scallop). ribosomal protein l9. |
| Q8itb0 *aequipecten irradians* (bay scallop). ribosomal protein s13. |
| Q8itb8 *aequipecten irradians* (bay scallop). ribosomal protein l23a (fragment). |
| Q8itc2 *aequipecten irradians* (bay scallop). ribosomal protein l11. |
| Q8iu34 *crassostrea gigas* (pacific oyster). vitellogenin |
| Q8t6a0 *aplysia californica* (california sea hare). 60s ribosomal protein l18. |
| Q8tfa7 *neocallimastix frontalis* (rumen fungus). adp/atp carrier. |
| Q9bpl8 *metagonimus* *yokogawai*. cysteine proteinase (fragment). |
| Q9i9e4 *pleurodeles waltlii* (iberian ribbed newt). putative nuclear movement protein pnudc. |
| RL9_RAT 60S ribosomal protein L9 - *Rattus norvegicus* (Rat) |
| ROP1L_XENLA Ropporin-1-like protein - *Xenopus laevis* (African clawed frog) |
| RPAP2_MOUSE RNA polymerase II-associated protein 2 - *Mus musculus* (Mouse) |
| RPN1_RAT Dolichyl-diphosphooligosaccharide--protein glycosyltransferase subunit 1 precursor - *Rattus norvegicus* (Rat) |
| RPOA_EAVBU Replicase polyprotein 1ab - *Equine arteritis* virus (strain Bucyrus) (EAV) |
| RS6_APLCA 40S ribosomal protein S6 - *Aplysia californica* (California sea hare) |
| RSBNL_XENTR Round spermatid basic protein 1-like - *Xenopus tropicalis* (Western clawed frog) (*Silurana tropicalis*) |
| SBP1_BOVIN Selenium-binding protein 1 - *Bos taurus* (Bovine) |
| similar to type XXIII collagen alpha 1 chain [*Gallus gallus*] |
| SMRD2_HUMAN SWI/SNF-related matrix-associated actin-dependent regulator of chromatin subfamily D member 2 - *Homo sapiens* (Human) |
| SP2M_BACSU Stage II sporulation protein M - *Bacillus subtilis* |
| SPG3_YEAST Stationary phase protein 3 - *Saccharomyces cerevisiae* (Baker's yeast) |
| SQSTM_RAT Sequestosome-1 - *Rattus norvegicus* (Rat) |
| SQSTM_RAT Sequestosome-1 - *Rattus norvegicus* (Rat) |
| SSRB_CANFA Translocon-associated protein subunit beta precursor - *Canis familiaris* (Dog) |
| STX7A_DICDI Syntaxin-7A - *Dictyostelium discoideum* (Slime mold) |
| SYH_CYTH3 Histidyl-tRNA synthetase - *Cytophaga hutchinsonii* (strain ATCC 33406 / NCIMB 9469) |
| SYL_FERNB Leucyl-tRNA synthetase - *Fervidobacterium nodosum* (strain ATCC 35602/DSM 5306/Rt17-B1) |
| TBA_WHEAT Tubulin alpha chain - *Triticum aestivum* (Wheat) |
| TBA3_RAT Tubulin alpha-3 chain - *Rattus norvegicus* (Rat) |
| TEN4_DANRE Teneurin-4 - *Danio rerio* (Zebrafish) (*Brachydanio rerio*) |
| TETX_CLOTE Tetanus toxin precursor - *Clostridium tetani* |
| thioredoxin 2 [*Gallus gallus*] |
| TM104_PONPY Transmembrane protein 104 - *Pongo pygmaeus* (Bornean orangutan) |
| TPPP3_XENLA Tubulin polymerization-promoting protein family member 3-*Xenopus laevis* (African clawed frog) |
| TR112_CAEEL TRM112-like protein - *Caenorhabditis elegans* |
| TRPP_BACSU Probable tryptophan transport protein - *Bacillus subtilis* |
| UBIQ_XENLA Ubiquitin - *Xenopus laevis* (African clawed frog) |
| VG2_SPV1R Gene 2 protein - Spiroplasma virus SpV1-R8A2 B (SpV1) (Spiroplasma virus 1) |
| VSH_MUMPK Small hydrophobic protein - Mumps virus (strain Kilham) |
| Y1252_HAEIN Uncharacterized ABC transporter ATP-binding protein HI1252 - *Haemophilus influenzae* |
| Y242_MYCGE Uncharacterized protein MG242 - *Mycoplasma genitalium* |
| Y351_BUCAP UPF0020/UPF0064 protein BUsg_351 - *Buchnera aphidicola* subsp. *Schizaphis graminum* |
| Y397_MYCGE Uncharacterized protein MG397 - *Mycoplasma genitalium* |
| YCF1_SOLBU Putative membrane protein ycf1 - *Solanum bulbocastanum* (Wild potato) |
| YCF2_PHYPA Protein ycf2 - *Physcomitrella patens* (Moss) |
| YMF16_NEPOL Uncharacterized tatC-like protein ymf16 - *Nephroselmis olivacea* |
| YO13B_YEAST Putative uncharacterized protein YOL013W-B - *Saccharomyces cerevisiae* (Baker's yeast) |
| YURN_BACSU Probable ABC transporter permease protein yurN - *Bacillus subtilis* |

Table S10. Genes with higher expression in Rb mussels with low MOX content

| EST1002; EST1015; EST102; EST1022; EST1037; EST1041; EST1042; EST1048; EST105; EST1059; EST107; EST1122; EST1129; EST1145; EST1146; EST1156; EST1159; EST1161; EST1164; EST1165; EST1172; EST1181; EST1182; EST1194; EST120; EST1203; EST1219; EST122; EST1220; EST1221; EST1222; EST1228; EST1245; EST1249; EST1250; EST1258; EST1259; EST1264; EST1273; EST1279; EST1284; EST1286; EST1294; EST1307; EST131; EST1313; EST1315; EST1322; EST1327; EST1331; EST1335; EST1336; EST1344; EST1350; EST1352; EST1360; EST1361; EST1364; EST1368; EST1377; EST1394; EST140; EST1411; EST1415; EST1428; EST143; EST1432; EST1436; EST1446; EST1454; EST1458; EST147; EST1475; EST1480; EST1488; EST1491; EST1495; EST1506; EST1514; EST1517; EST1522; EST1524; EST1525; EST1529; EST1541; EST161; EST241; EST256; EST262; EST280; EST281; EST289; EST305; EST312; EST318; EST326; EST327; EST33; EST336; EST339; EST34; EST346; EST349; EST354; EST369; EST384; EST387; EST39; EST416; EST460; EST47; EST52; EST521; EST550; EST559; EST583; EST589; EST596; EST611; EST631; EST644; EST651; EST655; EST669; EST671; EST675; EST688; EST693; EST694; EST695; EST699; EST706; EST709; EST733; EST735; EST736; EST742; EST743; EST745; EST774; EST778; EST779; EST786; EST787; EST79; EST790; EST793; EST802; EST811; EST813; EST827; EST829; EST830; EST846; EST92; EST925; EST94; EST964; EST965; EST966; EST97; EST995 |
| --- |
| 17 sequences no_hits_found |
| 209L2_MACMU CD209 antigen-like protein 2 - *Macaca mulatta* (Rhesus macaque) |
| AAS_KLEP7 Bifunctional protein aas [Includes: 2-acylglycerophosphoethanolamine acyltransferase - *Klebsiella pneumoniae* subsp. *pneumoniae* (strain ATCC 700721 / MGH 78578) |
| AGAP007259-PA [*Anopheles gambiae* str. PEST] |
| ANTA_HYDMA Antistasin precursor - *Hydra magnipapillata* (Hydra) |
| APE2_SULTO Probable aminopeptidase 2 - *Sulfolobus tokodaii* |
| ATX1_PLAFA Probable cation-transporting ATPase 1 - *Plasmodium falciparum* |
| BSC6_YEAST Bypass of stop codon protein 6 - *Saccharomyces cerevisiae* (Baker's yeast) |
| CAV1_ATEAB Caveolin-1 - *Atelerix albiventris* (Middle-African hedgehog) |
| CC130_DICDI Coiled-coil domain-containing protein 130 homolog - *Dictyostelium discoideum* (Slime mold) |
| CHS1_CRYNV Chitin synthase 1-*Cryptococcus neoformans* var. *grubii* (*Filobasidiella neoformans* var. *grubii*) |
| CL004_PONPY Uncharacterized protein C12orf4 homolog - *Pongo pygmaeus* (Bornean orangutan) |
| CLC14_HUMAN C-type lectin domain family 14 member A precursor - *Homo sapiens* (Human) |
| COBA2_MOUSE Collagen alpha-2(XI) chain precursor - *Mus musculus* (Mouse) |
| CQ061_BUFBG UPF0451 protein C17orf61 homolog precursor - *Bufo bufo* *gargarizans* (Asian toad) |
| CTNA_DICDI Countin-1 precursor - *Dictyostelium discoideum* (Slime mold) |
| CYAA_YEAST Adenylate cyclase - *Saccharomyces cerevisiae* (Baker's yeast) |
| CYOE_ACIAD Protoheme IX farnesyltransferase - *Acinetobacter* sp. (strain ADP1) |
| DAPA_SACD2 Dihydrodipicolinate synthase - *Saccharophagus degradans* (strain 2-40/ATCC 43961/DSM 17024) |
| DAPF_BUCBP Diaminopimelate epimerase - *Buchnera aphidicola* subsp. *Baizongia pistaciae* |
| DBF20_YEAST Serine/threonine-protein kinase DBF20 - *Saccharomyces cerevisiae* (Baker's yeast) |
| DOT1L_HUMAN Histone-lysine N-methyltransferase, H3 lysine-79 specific - *Homo sapiens* (Human) |
| DPO4_METAC DNA polymerase IV - *Methanosarcina acetivorans* |
| EAA3_MOUSE Excitatory amino acid transporter 3 - *Mus musculus* (Mouse) |
| EF1A_SPOFR Elongation factor 1-alpha - *Spodoptera frugiperda* (Fall armyworm) |
| ENV_HV190 Envelope glycoprotein gp160 precursor - Human immunodeficiency virus type 1 (isolate 90CF056 group M subtype H) (HIV-1) |
| ETV6_MOUSE Transcription factor ETV6 - *Mus musculus* (Mouse) |
| EX5B_BUCAI Exodeoxyribonuclease V beta chain - *Buchnera aphidicola* subsp. *Acyrthosiphon pisum* (*Acyrthosiphon pisum* symbiotic bacterium) |
| EX7L_LACS1 Exodeoxyribonuclease 7 large subunit - *Lactobacillus salivarius* subsp. *salivarius* (strain UCC118) |
| FACR1_CHICK Fatty acyl-CoA reductase 1 - *Gallus gallus* (Chicken) |
| FTSK_BORBU DNA translocase ftsK - *Borrelia burgdorferi* (Lyme disease spirochete) |
| GO:0004364; F:glutathione transferase activity; NAS |
| GO:0005515; F:protein binding; IPI |
| GO:0005622; C:intracellular; IEA GO:0006412; P:protein biosynthesis; IEA |
| GO:0005737; C:cytoplasm; IEA GO:0008152; P:metabolism; IEA |
| GO:0016021; C:integral to membrane; IEA GO:0004872; F:receptor activity; IEA |
| GO:0016301; F:kinase activity; IEA |
| GP126_HUMAN Probable G-protein coupled receptor 126 precursor - *Homo sapiens* (Human) |
| GP182_MOUSE G-protein coupled receptor 182 - *Mus musculus* (Mouse) |
| GPDA_LACRF Glycerol-3-phosphate dehydrogenase [NAD(P)+] - *Lactobacillus reuteri* (strain ATCC 23272 / DSM 20016 / F275) |
| GSPN_KLEPN General secretion pathway protein N - *Klebsiella pneumoniae* |
| GST8_CAEEL Probable glutathione S-transferase 8 - *Caenorhabditis elegans* |
| heterogeneous nuclear ribonucleoprotein 27c [*Aedes aegypti*] |
| hypothetical protein GSPATT00034562001 [*Paramecium tetraurelia* strain d4-2] |
| hypothetical protein GSPATT00039216001 [*Paramecium tetraurelia* strain d4-2] |
| hypothetical protein PC105494.00.0 [*Plasmodium chabaudi* *chabaudi*] |
| hypothetical protein TA05475 [*Theileria annulata* strain Ankara] |
| ICB1_HUMAN Induced by contact to basement membrane 1 protein - *Homo sapiens* (Human) |
| IF2H_MOUSE Eukaryotic translation initiation factor 2 subunit 3, Y-linked - *Mus musculus* (Mouse) |
| ISPD_ACIAD 2-C-methyl-D-erythritol 4-phosphate cytidylyltransferase - *Acinetobacter* sp. (strain ADP1) |
| K1033_MOUSE Uncharacterized protein KIAA1033 - *Mus musculus* (Mouse) |
| KGUA_VIBCH Guanylate kinase - *Vibrio cholerae* |
| KNG1_MOUSE Kininogen-1 precursor [Contains: Kininogen-1 heavy chain; Bradykinin; Kininogen-1 light chain] - *Mus musculus* (Mouse) |
| KR412_HUMAN Keratin-associated protein 4-12 - *Homo sapiens* (Human) |
| KTNA1_HUMAN Katanin p60 ATPase-containing subunit A1 - *Homo sapiens* (Human) |
| LEF2_NPVOP Late expression factor 2 - *Orgyia pseudotsugata* |
| LGMN_MOUSE Legumain precursor - *Mus musculus* (Mouse) |
| MAGA2_HUMAN Melanoma-associated antigen 2 - *Homo sapiens* (Human) |
| MEC2_CAEEL Mechanosensory protein 2 - *Caenorhabditis elegans* |
| MGR2_XENTR Protein MGR2 homolog - *Xenopus tropicalis* (Western clawed frog) (*Silurana tropicalis*) |
| MID1_YEAST Stretch-activated cation channel MID1 precursor - *Saccharomyces cerevisiae* (Baker's yeast) |
| MTB2_BACSU Modification methylase BglII - *Bacillus subtilis* |
| MURD_TRIEI UDP-N-acetylmuramoylalanine--D-glutamate ligase - *Trichodesmium erythraeum* (strain IMS101) |
| MYBA_DICDI Myb-like protein A - *Dictyostelium discoideum* (Slime mold) |
| NADH dehydrogenase subunit 4 [*Bathymodiolus* sp.] |
| NASP_RAT Nuclear autoantigenic sperm protein - *Rattus norvegicus* (Rat) |
| NCTR1_MACFA Natural cytotoxicity triggering receptor 1 precursor - *Macaca fascicularis* (Crab eating macaque) (Cynomolgus monkey) |
| NOG1_RAT Nucleolar GTP-binding protein 1 - *Rattus norvegicus* (Rat) |
| NU2M_MYTED NADH-ubiquinone oxidoreductase chain 2 - *Mytilus edulis* (Blue mussel) |
| NU3M_RECAM NADH-ubiquinone oxidoreductase chain 3 - *Reclinomonas americana* |
| NU5M_MYTED NADH-ubiquinone oxidoreductase chain 5 - *Mytilus edulis* (Blue mussel) |
| NUDC_RAT Nuclear migration protein nudC - *Rattus norvegicus* (Rat) |
| OUTS_DICD3 Lipoprotein outS precursor - *Dickeya dadantii* (strain 3937) (*Erwinia chrysanthemi* (strain 3937) |
| PDE3B_RAT cGMP-inhibited 3',5'-cyclic phosphodiesterase B - *Rattus norvegicus* (Rat) |
| PK2L2_MOUSE Polycystic kidney disease 2-like 2 protein - *Mus musculus* (Mouse) |
| PLMN_RAT Plasminogen precursor - *Rattus norvegicus* (Rat) |
| PNCB_METMA Nicotinate phosphoribosyltransferase - *Methanosarcina mazei* (Methanosarcina frisia) |
| PPIA_RABIT Peptidyl-prolyl cis-trans isomerase A - *Oryctolagus cuniculus* (Rabbit) |
| PSME3_PONPY Proteasome activator complex subunit 3 - *Pongo pygmaeus* (Bornean orangutan) |
| PURQ_BARQU Phosphoribosylformylglycinamidine synthase 1 - *Bartonella quintana* (Rochalimaea quintana) |
| putative heat shock protein [*Saccharophagus degradans* 2-40] |
| Q13489 *homo sapiens* (human). baculoviral iap repeat-containing protein 3 (inhibitor of apoptosis protein 1) (hiap1) (hiap-1) (c-iap2) (tnfr2-traf signaling complex protein 1) (iap homolog c) (apoptosis inhibitor 2) (api2). |
| Q17286 *barbatia virescens*. hemoglobin (heterodimeric). |
| Q32n81 *xenopus laevis* (african clawed frog). hypothetical protein. |
| Q32np0 *xenopus laevis* (african clawed frog). hypothetical protein. |
| Q3lf65 *mytilus galloprovincialis* (mediterranean mussel). heat shock cognate 71 |
| Q3t0v7 *bos taurus* (bovine). mgc127033 protein. |
| Q3y546 *petromyzon marinus* (sea lamprey). carbonic anhydrase. |
| Q4ac21 *hydroides elegans* (calcareous tube worm). camp-dependent protein kinase a catalytic subunit. |
| Q4gxm8 *georissus* sp. apv-2005. ribosomal protein l6e (fragment). |
| Q4h451 *crassostrea gigas* (pacific oyster). ribosomal protein s5. |
| Q4l223 *penaeus monodon* (penoeid shrimp). signal transducer and activator of transcription. |
| Q4pm17 *ixodes scapularis* (black-legged tick) (deer tick). ribosomal protein l35a. |
| Q4pm80 *ixodes scapularis* (black-legged tick) (deer tick). atp synthase c subunit. |
| Q4ruz5 *tetraodon nigroviridis* (green puffer). chromosome 12 scaf14993, whole genome shotgun sequence. |
| Q4tmv7 *erythrobacter litoralis* htcc2594. hypothetical protein. |
| Q4vbu6 *brachydanio rerio* (zebrafish) (danio rerio). hypothetical protein zgc:109991. |
| Q54tr4 *dictyostelium discoideum* (slime mold). hypothetical protein |
| Q5bl46 *xenopus tropicalis* (western clawed frog) (*silurana tropicalis*). lsm2 homolog, u6 small nuclear rna associated. |
| Q5blj9 m ribosomal protein l27 (10, 11 days embryo whole body cdna, riken full- length enriched library, clone:2810446a16 product:ribosomal protein l27, full insert sequence) (crl-1722 l5178y-r cdna, riken full-length enriched library, clone:i7. |
| Q5dbb2 *schistosoma japonicum* (blood fluke). sjchgc04204 protein. |
| Q5dgp6 *schistosoma japonicum* (blood fluke). sjchgc05042 protein. |
| Q5en79 *aurelia aurita* (moon jelly). ganglioside m2 activator-like protein. |
| Q5zkx0 *gallus gallus* (chicken). hypothetical protein. |
| Q61908 *mus musculus* (mouse). p8 mtcp-1 protein (mature t-cell proliferation-1 type a) (mtcp-1 type a) (p8mtcp1). |
| Q63zk1 *xenopus laevis* (african clawed frog). loc494790 protein. |
| Q640k1 *xenopus laevis* (african clawed frog). loc494655 protein. |
| Q67uu9 *oryza sativa* (japonica cultivar-group). putative keratin associated protein. |
| Q684l6 *lithobius forficatus*. translation initiation factor 2 gamma subunit. |
| Q69bj5 *cebus apella* (brown-capped capuchin). ubiquinol-cytochrome c oxidoreductase subunit 9 and rieske iron sulfur protein (fragment). |
| Q6bd10 *mytilus galloprovincialis* (mediterranean mussel). elongation factor 1 alpha. |
| Q6ddl4 *xenopus laevis* (african clawed frog). loc398446 protein. |
| Q6e6j7 *cricetulus griseus* (chinese hamster). heterochromatin protein 1 beta. |
| Q6h3x5 *pinctada fucata* (pearl oyster). qm protein. |
| Q6nri5 *xenopus laevis* (african clawed frog). mgc83793 protein. |
| Q6paa7 *xenopus laevis* (african clawed frog). mgc68517 protein. |
| Q6pc91 *brachydanio rerio* (zebrafish) (danio rerio). basic transcription factor 3-like 4. |
| Q6qm13 *lytechinus variegatus* (sea urchin). guanine nucleotide-binding protein g(q) alpha subunit (ec 3.6.5.1). |
| Q70sh0 *cepaea hortensis*. sialic acid binding lectin precursor. |
| Q75r50 *lymnaea stagnalis* (great pond snail). dec-3. |
| Q7pqg7 *anopheles gambiae* str. pest. ensangp00000011832. |
| Q7pqt6 *anopheles gambiae* str. pest. ensangp00000014750 (fragment). |
| Q7pyg5 *anopheles gambiae* str. pest. ensangp00000018356 (fragment). |
| Q7q8r0 *anopheles gambiae* str. pest. ensangp00000017984 (fragment). |
| Q7qik2 *anopheles gambiae* str. pest. ensangp00000021738. |
| Q7zuc4 *brachydanio rerio* (zebrafish) (*danio rerio*). ccdc6 protein (coiled-coil domain containing 6). |
| Q86j15 *dictyostelium discoideum* (slime mold). similar to kaposi's sarcoma-associated herpesvirus (kshv) (human herpesvirus 8). orf73 homolog (hypothetical protein). |
| Q86lp9 *branchiostoma belcheri* (amphoxius). hypothetical gaba(a) receptor-associated protein like-2. |
| Q8c847 *mus musculus* (mouse). 16 days embryo head cdna, riken full-length enriched library, clone:c130062g03 product:galactosidase, beta 1, full insert sequence. |
| Q8cdt7 *mus musculus* (mouse). protein c10orf107 homolog. |
| Q8is80 *euprymna scolopes*. 60s acidic ribosomal protein (fragment). |
| Q8it43 *theromyzon tessulatum* (leech). cystatin b. |
| Q8k3x6 *mus musculus* (mouse). harmonin-interacting ankyrin-repeat containing protein (harp). |
| Q8r562 *mus musculus* (mouse). ribonucleoprotein. |
| Q8sz15 *drosophila melanogaster* (fruit fly). re23670p (cg10914-pa). |
| Q8vhs2 *mus musculus* (mouse). crumbs protein homolog 1 precursor. |
| Q99jc0 *rattus norvegicus* (rat). rrna promoter binding protein. |
| Q9bnw3 *chaetopleura apiculata* (common eastern chiton). elongation factor-2 (fragment). |
| Q9cpt5 m 10, 11 days embryo whole body cdna, riken full-length enriched library, clone:2810436c08 product:hypothetical protein, full insert sequence (adult male kidney cdna, riken full-length enriched library, clone:0610030b22 product:dna segme. |
| Q9cqk5 m 18-day embryo whole body cdna, riken full-length enriched library, clone:1110002k21 product:hypothetical protein, full insert sequence (keratinocytes associated protein 2) (adult male kidney cdna, riken full-length enriched library, cl. |
| Q9czj2 *mus musculus* (mouse). heat shock 70 kda protein 12b. |
| Q9lii5 *arabidopsis thaliana* (mouse-ear cress). *arabidopsis thaliana* genomic dna, chromosome 3, tac clone:k24m9. |
| Q9u5r6 *entodinium caudatum*. putative ribosomal protein (fragment). |
| Q9vvu6 *drosophila melanogaster* (fruit fly). cg6841-pa (ld04472p). |
| Q9w0y2 *drosophila melanogaster* (fruit fly). hypothetical upf0131 protein cg2811. |
| RDH13_HUMAN Retinol dehydrogenase 13 - *Homo sapiens* (Human) |
| RF1_CLONN Peptide chain release factor 1 - *Clostridium novyi* (strain NT) |
| RIR1_CAEEL Ribonucleoside-diphosphate reductase large subunit - *Caenorhabditis elegans* |
| RL16_NEUCR 60S ribosomal protein L16 - *Neurospora crassa* |
| RL23_DROME 60S ribosomal protein L23 - *Drosophila melanogaster* (Fruit fly) |
| RPOB_AMBTC DNA-directed RNA polymerase subunit beta - *Amborella trichopoda* |
| RR4_ASTLO Plastid 30S ribosomal protein S4 - *Astasia longa* (Euglenophycean alga) |
| RS14_DROME 40S ribosomal protein S14 - *Drosophila melanogaster* (Fruit fly) |
| RS25_BRABE 40S ribosomal protein S25 - *Branchiostoma belcheri* (Amphioxus) |
| RS4_DROME 40S ribosomal protein S4 - *Drosophila melanogaster* (Fruit fly) |
| RS4_IXOSC 40S ribosomal protein S4 - *Ixodes scapularis* (Black-legged tick) (Deer tick) |
| RS7_BIFAA 30S ribosomal protein S7 - *Bifidobacterium adolescentis* (strain ATCC 15703 / DSM 20083) |
| RSHL3_HUMAN Radial spokehead-like protein 3 - *Homo sapiens* (Human) |
| S230_PLAFO Transmission-blocking target antigen S230 precursor - *Plasmodium falciparum* (isolate NF54) |
| SAHH_CAEEL Adenosylhomocysteinase - *Caenorhabditis elegans* |
| similar to PHD finger protein 15, [*Monodelphis domestica*] |
| SMC2_SCHPO Structural maintenance of chromosomes protein 2 - *Schizosaccharomyces pombe* (Fission yeast) |
| SNX41_YEAST Sorting nexin-41 - *Saccharomyces cerevisiae* (Baker's yeast) |
| SPD2A_DANRE SH3 and PX domain-containing protein 2A - *Danio rerio* (Zebrafish) (*Brachydanio rerio*) |
| SPD2A_MOUSE SH3 and PX domain-containing protein 2A - *Mus musculus* (Mouse) |
| SYF1_DICDI Pre-mRNA-splicing factor SYF1 - *Dictyostelium discoideum* (Slime mold) |
| TBAT_ONCMY Tubulin alpha chain, testis-specific - *Oncorhynchus mykiss* (Rainbow trout) (*Salmo gairdneri*) |
| TBB7_CHICK Tubulin beta-7 chain - *Gallus gallus* (Chicken) |
| TEKT3_MOUSE Tektin-3 - *Mus musculus* (Mouse) |
| transmembrane BAX inhibitor motif containing 4 [*Homo sapiens*] |
| TYRO_STRAT Tyrosinase - *Streptomyces antibioticus* |
| Vacuolar H ATPase family member (vha-17) [*Caenorhabditis elegans*] |
| VG56_ICHV1 Uncharacterized gene 56 protein - *Ictalurid herpesvirus* 1 (IcHV-1) (Channel catfish herpesvirus) |
| VIGLN_MOUSE Vigilin - *Mus musculus* (Mouse) |
| VP26B_PONPY Vacuolar protein sorting-associated protein 26B - *Pongo pygmaeus* (Bornean orangutan) |
| WBS22_BOVIN Uncharacterized methyltransferase WBSCR22 - *Bos taurus* (Bovine) |
| Y1428_HELPY UPF0063 protein HP_1428 - *Helicobacter pylori* (Campylobacter pylori) |
| Y539_AQUAE Uncharacterized protein aq_539 - *Aquifex aeolicus* |
| Y917_AQUAE Uncharacterized protein aq_917 - *Aquifex aeolicus* |
| YL769_MIMIV Putative ankyrin repeat protein L769 - *Acanthamoeba polyphaga* mimivirus (APMV) |
| ZDHC6_DICDI Putative ZDHHC-type palmitoyltransferase 6 - *Dictyostelium discoideum* (Slime mold) |

Table S11. Genes commonly regulated by SOX and MOX at MG

| EST1048; EST1062; EST112; EST1137; EST1213; EST203; EST377; EST385; EST437; EST651; EST687 |
| --- |
| CA151_MOUSE UPF0327 protein C1orf151 homolog - *Mus musculus* (Mouse) |
| CSLC4_ARATH Xyloglucan glycosyltransferase 4 - *Arabidopsis thaliana* (Mouse-ear cress) |
| DAF19_CAEEL RFX-like transcription factor daf-19 - *Caenorhabditis elegans* |
| DISP1_DANRE Protein dispatched homolog 1 - *Danio rerio* (Zebrafish) (*Brachydanio rerio*) |
| GO:0016021; C:integral to membrane; IEA GO:0005488; F:binding; IEA |
| HEM1_PROM9 Glutamyl-tRNA reductase - *Prochlorococcus marinus* (strain MIT 9312) |
| hypothetical protein GSPATT00002614001 [*Paramecium tetraurelia* |
| MATK_ATRJA Maturase K - *Atractylodes japonica* |
| Q3hri5 *mytilus edulis* (blue mussel). x-box binding protein 1 (fragment). |
| Q3uj73 *mus musculus* (mouse). cdna, riken full-length enriched library, clone:i920030a10 product:tubulin beta-2 chain homolog. |
| Q4rf58 *tetraodon nigroviridis* (green puffer). chromosome 14 scaf15120, whole genome shotgun sequence. |
| Q4spx2 *tetraodon nigroviridis* (green puffer). chromosome 7 scaf14536, whole genome shotgun sequence. |
| Q4vbt4 *brachydanio rerio* (zebrafish) (danio rerio). translation elongation factor 1-gamma. |
| Q56tz4 *rattus norvegicus* (rat). macrophage-inducible c-type lectin. |
| Q6gq33 *xenopus laevis* (african clawed frog). mgc80379 protein. |
| Q8wp36 *suberites domuncula* (sponge). col protein. |
| Q8ws60 *branchiostoma floridae* (florida lancelet) (amphioxus). endonuclease/reverse transcriptase. |
| Q95w89 *branchiostoma belcheri* (amphoxius). 60s ribosomal protein l37a. |
| Q9i9e4 *pleurodeles waltlii* (iberian ribbed newt). putative nuclear movement protein pnudc. |
| RUD3_YEAST GRIP domain-containing protein RUD3 - *Saccharomyces cerevisiae* (Baker's yeast) |
| TRME_PROM0 tRNA modification GTPase trmE - *Prochlorococcus marinus* (strain MIT 9301) |
| unnamed protein product [*Candida glabrata*] |
| YCX2_CYAPA Uncharacterized 24.3 kDa protein in psbH-rpl11 intergenic region - *Cyanophora paradoxa* |
| YKAA_CAEEL Uncharacterized amino-acid permease B0303.11 - *Caenorhabditis elegans* |

Table S12. Genes commonly regulated by SOX and MOX at Rb

| EST100; EST1015; EST102; EST1022; EST1040; EST1042; EST1048; EST1081; EST1122; EST1129; EST1146; EST1172; EST1182; EST1230; EST1249; EST1250; EST1259; EST129; EST1327; EST134; EST1343; EST136; EST1361; EST1362; EST1377; EST1382; EST1394; EST1415; EST1446; EST1487; EST1511; EST1525; EST1529; EST161; EST164; EST202; EST239; EST253; EST281; EST352; EST354; EST355; EST37; EST384; EST389; EST390; EST392; EST461; EST47; EST513; EST52; EST560; EST571; EST602; EST635; EST658; EST668; EST675; EST694; EST695; EST699; EST706; EST727; EST733; EST742; EST743; EST761; EST763; EST774; EST78; EST787; EST789; EST793; EST80; EST802; EST813; EST82; EST825; EST832; EST843; EST846; EST854; EST884; EST894; EST902; EST928; EST960; EST966; EST977 |
| --- |
| 209L2_MACMU CD209 antigen-like protein 2 - *Macaca mulatta* (Rhesus macaque) |
| AAS_KLEP7 Bifunctional protein aas [Includes: 2-acylglycerophosphoethanolamine acyltransferase - *Klebsiella pneumoniae* subsp. *pneumoniae* (strain ATCC 700721 / MGH 78578) |
| ACOD_CYPCA Acyl-CoA desaturase - *Cyprinus carpio* (Common carp) |
| ACTP2_CYMEC Echotoxin-2 precursor - *Cymatium echo* (Giant triton) (Monoplex echo) |
| ANTA_HYDMA Antistasin precursor - *Hydra magnipapillata* (Hydra) |
| ATX1_PLAFA Probable cation-transporting ATPase 1 - *Plasmodium falciparum* |
| BSC6_YEAST Bypass of stop codon protein 6 - *Saccharomyces cerevisiae* (Baker's yeast) |
| CD97_BOVIN CD97 antigen precursor - *Bos taurus* (Bovine) |
| CHS1_CRYNV Chitin synthase 1 - *Cryptococcus neoformans* var. *grubii* (Filobasidiella neoformans var. grubii) |
| COAT4_MIMIV Probable capsid protein 4 - *Acanthamoeba polyphaga* mimivirus (APMV) |
| COX1_MYTED Cytochrome c oxidase subunit 1 - *Mytilus edulis* (Blue mussel) |
| CTNA_DICDI Countin-1 precursor - *Dictyostelium discoideum* (Slime mold) |
| DAZP1_MOUSE DAZ-associated protein 1 - *Mus musculus* (Mouse) |
| DBF20_YEAST Serine/threonine-protein kinase DBF20 - *Saccharomyces cerevisiae* (Baker's yeast) |
| DHGL_DROME Glucose dehydrogenase [acceptor] precursor - *Drosophila melanogaster* (Fruit fly) |
| diaphanous [*Aedes aegypti*] |
| EAA3_MOUSE Excitatory amino acid transporter 3 - *Mus musculus* (Mouse) |
| ERF1X_ARATH Eukaryotic peptide chain release factor subunit 1-1 - *Arabidopsis thaliana* (Mouse-ear cress) |
| ETV6_MOUSE Transcription factor ETV6 - *Mus musculus* (Mouse) |
| FTSK_BORBU DNA translocase ftsK - *Borrelia burgdorferi* (Lyme disease spirochete) |
| GBLP_NICPL Guanine nucleotide-binding protein subunit beta-like protein - *Nicotiana plumbaginifolia* (Leadwort-leaved tobacco) |
| GO:0004364; F:glutathione transferase activity; NAS |
| GO:0005488; F:binding; IEA |
| GO:0005529; F:sugar binding; IEA |
| GO:0016021; C:integral to membrane; IEA GO:0005488; F:binding; IEA |
| GO:0016021; C:integral to membrane; IEA GO:0015031; P:protein transport; IEA |
| GPDA_LACRF Glycerol-3-phosphate dehydrogenase [NAD(P)+] - *Lactobacillus reuteri* (strain ATCC 23272 / DSM 20016 / F275) |
| GRN_HUMAN Granulins precursor - *Homo sapiens* (Human) |
| GSPN_KLEPN General secretion pathway protein N - *Klebsiella pneumoniae* |
| IAA3_ARATH Auxin-responsive protein IAA3 - *Arabidopsis thaliana* (Mouse-ear cress) |
| KTHY_HUMAN Thymidylate kinase - *Homo sapiens* (Human) |
| LEF2_NPVOP Late expression factor 2 - *Orgyia pseudotsugata* |
| MCAT_MOUSE Mitochondrial carnitine/acylcarnitine carrier protein - *Mus musculus* (Mouse) |
| MDM31_YEAST Mitochondrial distribution and morphology protein 31, mitochondrial precursor - *Saccharomyces cerevisiae* (Baker's yeast) |
| MGR2_XENTR Protein MGR2 homolog - *Xenopus tropicalis* (Western clawed frog) (Silurana tropicalis) |
| MID1_YEAST Stretch-activated cation channel MID1 precursor - *Saccharomyces cerevisiae* (Baker's yeast) |
| MTB2_BACSU Modification methylase BglII - *Bacillus subtilis* |
| NASP_RAT Nuclear autoantigenic sperm protein - *Rattus norvegicus* (Rat) |
| NCTR1_MACFA Natural cytotoxicity triggering receptor 1 precursor - *Macaca fascicularis* (Crab eating macaque) (Cynomolgus monkey) |
| NOL11_PONPY Nucleolar protein 11 - *Pongo pygmaeus* (Bornean orangutan) |
| NU2M_MYTED NADH-ubiquinone oxidoreductase chain 2 - *Mytilus edulis* (Blue mussel) |
| NUDC_RAT Nuclear migration protein nudC - *Rattus norvegicus* (Rat) |
| O14463 *schizosaccharomyces pombe* (fission yeast). thioredoxin (tr). |
| O44231 *anthocidaris crassispina* (sea urchin). outer arm dynein light chain 1. |
| P41824 *aplysia californica* (california sea hare). y-box factor homolog (apy1). |
| P42578 *lymnaea stagnalis* (great pond snail). yolk ferritin precursor (ec 1.16.3.1). |
| PDE3B_RAT cGMP-inhibited 3',5'-cyclic phosphodiesterase B - *Rattus norvegicus* (Rat) |
| PGES2_DANRE Prostaglandin E synthase 2 - *Danio rerio* (Zebrafish) (*Brachydanio rerio*) |
| PK2L2_MOUSE Polycystic kidney disease 2-like 2 protein - *Mus musculus* (Mouse) |
| predicted protein [*Nematostella vectensis* |
| Q17286 *barbatia virescens*. hemoglobin (heterodimeric). |
| Q32sg8 *brachydanio rerio* (zebrafish) (*danio rerio*). sodium calcium exchanger 1h. |
| Q33604 *lamna nasus* (porbeagle) (*squalus nasus*). cytochrome b. |
| Q4h451 *crassostrea gigas* (pacific oyster). ribosomal protein s5. |
| Q4pm17 *ixodes scapularis* (black-legged tick) (deer tick). ribosomal protein l35a. |
| Q4ruz5 *tetraodon nigroviridis* (green puffer). chromosome 12 scaf14993, whole genome shotgun sequence. |
| Q5i0s5 xenopus tropicalis (western clawed frog) (silurana tropicalis). c1ql1-prov protein (fragment). |
| Q5rgn5 *brachydanio rerio* (zebrafish) (*danio rerio*). novel protein similar to human and mouse complement component 1, q subcomponent-like 1 (c1ql1) (fragment). |
| Q5tmp1 *anopheles gambiae* str. pest. ensangp00000028262. |
| Q5zkx0 *gallus gallus* (chicken). hypothetical protein. |
| Q67uu9 oryza sativa (japonica cultivar-group). putative keratin associated protein. |
| Q6bd10 *mytilus galloprovincialis* (mediterranean mussel). elongation factor 1 alpha. |
| Q6ddl4 *xenopus laevis* (african clawed frog). loc398446 protein. |
| Q6pbr1 *brachydanio rerio* (zebrafish) (danio rerio). loc402878 protein (fragment). |
| Q6pc91 *brachydanio rerio* (zebrafish) (danio rerio). basic transcription factor 3-like 4. |
| Q70ph4 *crassostrea gigas* (pacific oyster). omega class glutathione s-transferase. |
| Q75r50 *lymnaea stagnalis* (great pond snail). dec-3. |
| Q7pqg7 *anopheles gambiae* str. pest. ensangp00000011832. |
| Q7pyg5 *anopheles gambiae* str. pest. ensangp00000018356 (fragment). |
| Q7q8r0 *anopheles gambiae* str. pest. ensangp00000017984 (fragment). |
| Q86lp9 *branchiostoma belcheri* (amphoxius). hypothetical gaba(a) receptor-associated protein like-2. |
| Q8cdt7 *mus musculus* (mouse). protein c10orf107 homolog. |
| Q8is80 *euprymna scolopes*. 60s acidic ribosomal protein (fragment). |
| Q8ita5 *aequipecten irradians* (bay scallop). ribosomal protein l9. |
| Q8itb8 *aequipecten irradians* (bay scallop). ribosomal protein l23a (fragment). |
| Q8itc2 *aequipecten irradians* (bay scallop). ribosomal protein l11. |
| Q8tfa7 *neocallimastix frontalis* (rumen fungus). adp/atp carrier. |
| Q99jc0 *rattus norvegicus* (rat). rrna promoter binding protein. |
| Q9i9e4 *pleurodeles waltlii* (iberian ribbed newt). putative nuclear movement protein pnudc. |
| Q9lii5 *arabidopsis thaliana* (mouse-ear cress). arabidopsis thaliana genomic dna, chromosome 3, tac clone:k24m9. |
| Q9u5r6 *entodinium caudatum*. putative ribosomal protein (fragment). |
| RDH13_HUMAN Retinol dehydrogenase 13 - *Homo sapiens* (Human) |
| RPN1_RAT Dolichyl-diphosphooligosaccharide--protein glycosyltransferase subunit 1 precursor - *Rattus norvegicus* (Rat) |
| RS14_DROME 40S ribosomal protein S14 - *Drosophila melanogaster* (Fruit fly) |
| RS4_DROME 40S ribosomal protein S4 - *Drosophila melanogaster* (Fruit fly) |
| RS4_IXOSC 40S ribosomal protein S4 - *Ixodes scapularis* (Black-legged tick) (Deer tick) |
| RSBNL_XENTR Round spermatid basic protein 1-like - *Xenopus tropicalis* (Western clawed frog) (*Silurana* *tropicalis*) |
| S230_PLAFO Transmission-blocking target antigen S230 precursor - *Plasmodium falciparum* (isolate NF54) |
| SAHH_CAEEL Adenosylhomocysteinase - *Caenorhabditis elegans* |
| SP2M_BACSU Stage II sporulation protein M - *Bacillus subtilis* |
| SYF1_DICDI Pre-mRNA-splicing factor SYF1 - *Dictyostelium discoideum* (Slime mold) |
| SYH_CYTH3 Histidyl-tRNA synthetase - *Cytophaga hutchinsonii* (strain ATCC 33406 / NCIMB 9469) |
| SYL_FERNB Leucyl-tRNA synthetase - *Fervidobacterium nodosum* (strain ATCC 35602/DSM 5306/Rt17-B1) |
| TBAT_ONCMY Tubulin alpha chain, testis-specific - Oncorhynchus mykiss (Rainbow trout) (Salmo gairdneri) |
| transmembrane BAX inhibitor motif containing 4 [*Homo sapiens*] |
| TRPP_BACSU Probable tryptophan transport protein - *Bacillus subtilis* |
| Vacuolar H ATPase family member (vha-17) [*Caenorhabditis elegans*] |
| VP26B_PONPY Vacuolar protein sorting-associated protein 26B - *Pongo pygmaeus* (Bornean orangutan) |
| VSH_MUMPK Small hydrophobic protein - Mumps virus (strain Kilham) |
| Y539_AQUAE Uncharacterized protein aq_539 - *Aquifex aeolicus* |
| YCF2_PHYPA Protein ycf2 - *Physcomitrella patens* (Moss) |
| YL769_MIMIV Putative ankyrin repeat protein L769 - *Acanthamoeba polyphaga* mimivirus (APMV) |
| ZDHC6_DICDI Putative ZDHHC-type palmitoyltransferase 6 - *Dictyostelium discoideum* (Slime mold) |

Table S13. Genes commonly regulated by SOX at Rb and MG

| EST1213; EST1224; EST1298; EST1343; EST1382; EST185; EST31; EST355; EST37; EST385; EST437; EST438; EST481; EST610; EST764; EST832; EST916 |
| --- |
| CA151_MOUSE UPF0327 protein C1orf151 homolog - *Mus musculus* (Mouse) |
| DAF19_CAEEL RFX-like transcription factor daf-19 - *Caenorhabditis elegans* |
| DPO3B_CHLTR DNA polymerase III subunit beta - *Chlamydia trachomatis* |
| EAA3_MOUSE Excitatory amino acid transporter 3 - *Mus musculus* (Mouse) |
| FTSK_BORBU DNA translocase ftsK - *Borrelia burgdorferi* (Lyme disease spirochete) |
| GO:0004563; F:beta-N-acetylhexosaminidase activity; IEA GO:0005975; P:carbohydrate metabolism; IEA |
| GO:0005529; F:sugar binding; IEA |
| GO:0006950; P:response to stress; IEA |
| GO:0016021; C:integral to membrane; IEA GO:0005488; F:binding; IEA |
| MDMC_STRMY O-methyltransferase mdmC - *Streptomyces mycarofaciens* |
| Q4s0q0 *tetraodon nigroviridis* (green puffer). chromosome 2 scaf14781, whole genome shotgun sequence. |
| Q5f1m8 *mytilus galloprovincialis* (mediterranean mussel). nadh dehydrogenase subunit 4. |
| Q6ddm3 *xenopus laevis* (african clawed frog). pecr-prov protein. |
| Q7pyg5 *anopheles gambiae* str. pest. ensangp00000018356 (fragment). |
| Q8ws60 *branchiostoma floridae* (florida lancelet) (amphioxus). endonuclease/reverse transcriptase. |
| Q95nn1 *tribolium castaneum* (red flour beetle). tryptophan oxygenase. |
| Q9i9e4 *pleurodeles waltlii* (iberian ribbed newt). putative nuclear movement protein pnudc. |
| Q9vf83 *drosophila melanogaster* (fruit fly). cg31301-pa (ld33178p). |
| RSMF_SHEON Ribosomal RNA small subunit methyltransferase F - *Shewanella oneidensis* |

Table S14. Genes commonly regulated by MOX at Rb and MG

| EST1006; EST1048; EST1091; EST1161; EST1196; EST1230; EST1259; EST1264; EST1273; EST1322; EST1327; EST1350; EST1360; EST1394; EST143; EST1432; EST1446; EST147; EST1511; EST1539; EST161; EST171; EST192; EST241; EST256; EST265; EST349; EST367; EST387; EST487; EST611; EST631; EST635; EST644; EST651; EST668; EST675; EST693; EST745; EST787; EST825; EST928; EST966; EST975 |
| --- |
| AGAP007259-PA [*Anopheles gambiae* str. PEST] |
| ANX11_BOVIN Annexin A11 - *Bos taurus* (Bovine) |
| ATX1_PLAFA Probable cation-transporting ATPase 1 - *Plasmodium falciparum* |
| BSC6_YEAST Bypass of stop codon protein 6 - *Saccharomyces cerevisiae* (Baker's yeast) |
| CQ061_BUFBG UPF0451 protein C17orf61 homolog precursor - *Bufo bufo gargarizans* (Asian toad) |
| DBF20_YEAST Serine/threonine-protein kinase DBF20 - *Saccharomyces cerevisiae* (Baker's yeast) |
| EAF7_YEAST Chromatin modification-related protein EAF7 - *Saccharomyces cerevisiae* (Baker's yeast) |
| EF1A_SPOFR Elongation factor 1-alpha - *Spodoptera frugiperda* (Fall armyworm) |
| ERF1X_ARATH Eukaryotic peptide chain release factor subunit 1-1 - *Arabidopsis thaliana* (Mouse-ear cress) |
| GO:0016021; C:integral to membrane; IEA GO:0015031; P:protein transport; IEA |
| hypothetical protein TA05475 [*Theileria annulata* strain Ankara] |
| NOG1_RAT Nucleolar GTP-binding protein 1 - *Rattus norvegicus* (Rat) |
| NOL11_PONPY Nucleolar protein 11 - *Pongo pygmaeus* (Bornean orangutan) |
| O44231 *anthocidaris crassispina* (sea urchin). outer arm dynein light chain 1. |
| P41824 *aplysia californica* (california sea hare). y-box factor homolog (apy1). |
| P42578 *lymnaea stagnalis* (great pond snail). yolk ferritin precursor (ec 1.16.3.1). |
| P56974 *mus musculus* (mouse). pro-neuregulin-2, membrane-bound isoform precursor (pro-nrg2) [contains: neuregulin-2 (nrg-2) (divergent of neuregulin 1) (don-1)]. |
| predicted protein [*Nematostella vectensis*] |
| Q32n81 *xenopus laevis* (african clawed frog). hypothetical protein. |
| Q32sg8 *brachydanio rerio* (zebrafish) (danio rerio). sodium calcium exchanger 1h. |
| Q4gxm8 *georissus* sp. apv-2005. ribosomal protein l6e (fragment). |
| Q4pm17 *ixodes scapularis* (black-legged tick) (deer tick). ribosomal protein l35a. |
| Q4rf58 *tetraodon nigroviridis* (green puffer). chromosome 14 scaf15120, whole genome shotgun sequence. |
| Q4tmv7 *erythrobacter litoralis* htcc2594. hypothetical protein. |
| Q4v910 *brachydanio rerio* (zebrafish) (*danio rerio*). loc553269 protein (fragment). |
| Q5i0s5 *xenopus tropicalis* (western clawed frog) (*silurana tropicalis*). c1ql1-prov protein (fragment). |
| Q5xj47 *brachydanio rerio* (zebrafish) (*danio rerio*). h1m protein (fragment). |
| Q6e6j7 *cricetulus griseus* (chinese hamster). heterochromatin protein 1 beta. |
| Q6gq33 *xenopus laevis* (african clawed frog). mgc80379 protein. |
| Q6j0s6 *branchiostoma belcheri* tsingtaunese. duf614 protein. |
| Q6pc91 *brachydanio rerio* (zebrafish) (danio rerio). basic transcription factor 3-like 4. |
| Q6qm13 *lytechinus variegatus* (sea urchin). guanine nucleotide-binding protein g(q) alpha subunit (ec 3.6.5.1). |
| Q7pqg7 *anopheles gambiae* str. pest. ensangp00000011832. |
| Q7qia8 *anopheles gambiae* str. pest. ensangp00000020311. |
| Q7qlc5 *anopheles gambiae* str. pest. ensangp00000001833. |
| Q7zyn6 *xenopus laevis* (african clawed frog). loc398452 protein (fragment |
| Q86lp9 *branchiostoma belcheri* (amphoxius). hypothetical gaba(a) receptor-associated protein like-2. |
| Q8r562 *mus musculus* (mouse). ribonucleoprotein. |
| Q8vhs2 *mus musculus* (mouse). crumbs protein homolog 1 precursor. |
| Q99jc0 *rattus norvegicus* (rat). rrna promoter binding protein. |
| Q9cqk5 m 18-day embryo whole body cdna, riken full-length enriched library, clone:1110002k21 product:hypothetical protein, full insert sequence (keratinocytes associated protein 2) (adult male kidney cdna, riken full-length enriched library, cl. |
| Q9i9e4 *pleurodeles waltlii* (iberian ribbed newt). putative nuclear movement protein pnudc. |
| Q9u5r6 *entodinium caudatum*. putative ribosomal protein (fragment). |
| RS14_DROME 40S ribosomal protein S14 - *Drosophila melanogaster* (Fruit fly) |
| RS7_DANRE 40S ribosomal protein S7 - *Danio rerio* (Zebrafish) (*Brachydanio rerio*) |
| RSHL3_HUMAN Radial spokehead-like protein 3 - *Homo sapiens* (Human) |
| S230_PLAFO Transmission-blocking target antigen S230 precursor - *Plasmodium falciparum* (isolate NF54) |
| SAHH_CAEEL Adenosylhomocysteinase - *Caenorhabditis elegans* |
| SPD2A_DANRE SH3 and PX domain-containing protein 2A - *Danio rerio* (Zebrafish) (*Brachydanio rerio*) |
| TETX_CLOTE Tetanus toxin precursor - *Clostridium tetani* |
| thioredoxin 2 [*Gallus gallus*] |
| transmembrane BAX inhibitor motif containing 4 [*Homo sapiens*] |
| TYRO_STRAT Tyrosinase - *Streptomyces antibioticus* |
| Y1252_HAEIN Uncharacterized ABC transporter ATP-binding protein HI1252 - *Haemophilus influenzae* |
| YCF1_SOLBU Putative membrane protein ycf1 - *Solanum bulbocastanum* (Wild potato) |
| YCF2_PHYPA Protein ycf2 - *Physcomitrella patens* (Moss) |
| YURN_BACSU Probable ABC transporter permease protein yurN - *Bacillus subtilis* |
| ZDHC6_DICDI Putative ZDHHC-type palmitoyltransferase 6 - *Dictyostelium discoideum* (Slime mold) |

Table S15. Genes with higher expression in MG mussels with low ATP sulfurylase expression

| EST1010; EST1044; EST1045; EST1083; EST1118; EST1148; EST1173; EST120; EST1266; EST135; EST1362; EST143; EST1490; EST196; EST223; EST233; EST256; EST29; EST33; EST36; EST449; EST451; EST508; EST51; EST522; EST60; EST612; EST626; EST708; EST749; EST849; EST859; EST958; EST966; EST998 |
| --- |
| 5 sequences no_hits_found |
| COCA1_RABIT Collagen alpha-1(XII) chain - Oryctolagus cuniculus (Rabbit) |
| CYOE_ACIAD Protoheme IX farnesyltransferase - Acinetobacter sp. (strain ADP1) |
| CYST_ANTFO Probable sulfate transport system permease protein cysT - Anthoceros formosae (Hornwort) |
| esterase/lipase hi0193 [Plasmodium yoelii yoelii str |
| FBRL_DROME rRNA 2'-O-methyltransferase fibrillarin - Drosophila melanogaster (Fruit fly) |
| G3P3_RHIRA Glyceraldehyde-3-phosphate dehydrogenase 3 - Rhizomucor racemosus (Mucor circinelloides f. lusitanicus) |
| GO:0004563; F:beta-N-acetylhexosaminidase activity; IEA GO:0005975; P:carbohydrate metabolism; IEA |
| GO:0005515; F:protein binding; IPI |
| GO:0009044; F:xylan 1,4-beta-xylosidase activity; IEA GO:0005975; P:carbohydrate metabolism; IEA |
| GO:0016301; F:kinase activity; IEA |
| GR47B_DROME Putative gustatory receptor 47b - Drosophila melanogaster (Fruit fly) |
| IMB_DROME Importin subunit beta - Drosophila melanogaster (Fruit fly) |
| ISPZ_AERHH Probable intracellular septation protein - Aeromonas hydrophila subsp. hydrophila (strain ATCC 7966 / NCIB 9240) |
| KGUA_VIBCH Guanylate kinase - Vibrio cholerae |
| KNG1_MOUSE Kininogen-1 precursor [Contains: Kininogen-1 heavy chain; Bradykinin; Kininogen-1 light chain] - Mus musculus (Mouse) |
| LPE10_CANGA Inner membrane magnesium transporter LPE10, mitochondrial precursor - Candida glabrata (Yeast) (Torulopsis glabrata) |
| MTB2_BACSU Modification methylase BglII - Bacillus subtilis |
| NU1M_PODAN NADH-ubiquinone oxidoreductase chain 1 - Podospora anserina |
| NU5M_ASCSU NADH-ubiquinone oxidoreductase chain 5 - Ascaris suum (Pig roundworm) (Ascaris lumbricoides) |
| OR4X2_HUMAN Olfactory receptor 4X2 - Homo sapiens (Human) |
| PE2R2_CANFA Prostaglandin E2 receptor EP2 subtype - Canis familiaris (Dog) |
| phospholipase A2, group IVB [Bos taurus] |
| POLG_BYMV Genome polyprotein [Contains: P1 proteinase - Bean yellow mosaic virus |
| Q3zb81 homo sapiens (human). ctcl tumor antigen l14-2. |
| Q499u1 rattus norvegicus (rat). hypothetical protein rgd1311914_predicted. |
| Q503m5 brachydanio rerio (zebrafish) (danio rerio). loc569147 protein (fragment). |
| Q5bjm2 rattus norvegicus (rat). cyclin b1 interacting protein 1. |
| Q5dfa9 schistosoma japonicum (blood fluke). sjchgc06248 protein. |
| Q5f1m8 mytilus galloprovincialis (mediterranean mussel). nadh dehydrogenase subunit 4. |
| Q5uen8 toxoptera citricida (brown citrus aphid). putative 14-3-3 protein epsilon. |
| Q6inu7 xenopus laevis (african clawed frog). mgc80281 protein. |
| Q70ml7 crassostrea gigas (pacific oyster). signal sequence receptor beta-like protein (fragment). |
| Q7pqg7 anopheles gambiae str. pest. ensangp00000011832. |
| Q7pqt6 anopheles gambiae str. pest. ensangp00000014750 (fragment). |
| Q7q7q5 anopheles gambiae str. pest. ensangp00000021588 (fragment). |
| Q7q7z4 anopheles gambiae str. pest. ensangp00000022226 (fragment). |
| Q8ifu3 mastotermes darwiniensis. beta-1,4-endoglucanase precursor (ec 3.2.1.4). |
| Q8is80 euprymna scolopes. 60s acidic ribosomal protein (fragment). |
| Q8mhz1 sus scrofa (pig). diacylglycerol acyltransferase. |
| Q92797 homo sapiens (human). symplekin. |
| Q95v47 artemia sanfranciscana (brine shrimp) (artemia franciscana). 70 kda heat shock protein. |
| Q9u5r6 entodinium caudatum. putative ribosomal protein (fragment). |
| Q9vmv2 drosophila melanogaster (fruit fly). cg31650-pa, isoform a (cg31650-pb, isoform b) (ld34388p). |
| RDH13_HUMAN Retinol dehydrogenase 13 - Homo sapiens (Human) |
| RL18_RAT 60S ribosomal protein L18 - Rattus norvegicus (Rat) |
| RPOC2_THAPS DNA-directed RNA polymerase subunit beta'' - Thalassiosira pseudonana (Marine diatom) |
| RS4_DROME 40S ribosomal protein S4 - Drosophila melanogaster (Fruit fly) |
| SAHH_CAEEL Adenosylhomocysteinase - Caenorhabditis elegans |
| SC5D2_ARATH Putative Delta(7)-sterol-C5(6)-desaturase 2 - Arabidopsis thaliana (Mouse-ear cress) |
| SMC2_SCHPO Structural maintenance of chromosomes protein 2 - Schizosaccharomyces pombe (Fission yeast) |
| SPRT_HAEDU Protein sprT - Haemophilus ducreyi |
| STE6_YEAST Mating factor A secretion protein STE6 - Saccharomyces cerevisiae (Baker's yeast) |
| SYI_SULSO Isoleucyl-tRNA synthetase - Sulfolobus solfataricus |
| TBA_WHEAT Tubulin alpha chain - Triticum aestivum (Wheat) |
| TPPP3_XENLA Tubulin polymerization-promoting protein family member 3 - Xenopus laevis (African clawed frog) |
| VP80_NPVAC Capsid protein p80 - Autographa californica nuclear polyhedrosis virus (AcMNPV) |
| YKD0_YEAST Putative uncharacterized protein YKL030W - Saccharomyces cerevisiae (Baker's yeast) |

Table S16. Genes with higher expression in MG mussels with high ATP sulfurylase expression

| EST1011; EST1023; EST1038; EST1043; EST1075; EST1113; EST1125; EST1129; EST1133; EST1168; EST1239; EST124; EST1282; EST1287; EST1302; EST131; EST132; EST1340; EST136; EST1392; EST1416; EST1423; EST1428; EST1450; EST1467; EST1535; EST1543; EST1554; EST158; EST162; EST188; EST206; EST210; EST224; EST239; EST248; EST251; EST259; EST260; EST276; EST285; EST293; EST3; EST303; EST333; EST339; EST362; EST386; EST436; EST439; EST447; EST450; EST454; EST464; EST501; EST521; EST548; EST578; EST669; EST699; EST710; EST714; EST728; EST744; EST748; EST757; EST758; EST76; EST775; EST777; EST834; EST843; EST844; EST860; EST871; EST872; EST882; EST89; EST912; EST915; EST93; EST931; EST933; EST940; EST955; EST968; EST982; EST997 |
| --- |
| 10 sequences no_hits_found |
| ABCA9_MOUSE ATP-binding cassette sub-family A member 9 - Mus musculus (Mouse) |
| ACTP2_CYMEC Echotoxin-2 precursor - Cymatium echo (Giant triton) (Monoplex echo) |
| AL9A1_RAT 4-trimethylaminobutyraldehyde dehydrogenase - Rattus norvegicus (Rat) |
| Aminoadipate aminotransferase [Bos taurus] |
| ARSJ_MOUSE Arylsulfatase J precursor - Mus musculus (Mouse) |
| C1QBP_CERAE Complement component 1 Q subcomponent-binding protein, mitochondrial precursor - Cercopithecus aethiops (Green monkey) (Grivet) |
| CI021_HUMAN UPF0308 protein C9orf21 - Homo sapiens (Human) |
| Delta-6 fatty acid desaturase (Fatty acid desaturase 2) |
| DNLJ_BARQU DNA ligase - Bartonella quintana (Rochalimaea quintana) |
| DPO3B_CHLTR DNA polymerase III subunit beta - Chlamydia trachomatis |
| DSE1_YARLI Protein DSE1 - Yarrowia lipolytica (Candida lipolytica) |
| EF1A_BOMMO Elongation factor 1-alpha - Bombyx mori (Silk moth) |
| EF2_CAEEL Elongation factor 2 - Caenorhabditis elegans |
| EIF3D_HUMAN Eukaryotic translation initiation factor 3 subunit D - Homo sapiens (Human) |
| ERP5_YEAST Protein ERP5 precursor - Saccharomyces cerevisiae (Baker's yeast) |
| EX5B_BUCAI Exodeoxyribonuclease V beta chain - Buchnera aphidicola subsp. Acyrthosiphon pisum (Acyrthosiphon pisum symbiotic bacterium) |
| FACR1_CHICK Fatty acyl-CoA reductase 1 - Gallus gallus (Chicken) |
| GAT28_ARATH GATA transcription factor 28 - Arabidopsis thaliana (Mouse-ear cress) |
| GO:0005524; F:ATP binding; IEA GO:0000166; F:nucleotide binding; IEA |
| GO:0005737; C:cytoplasm; IEA GO:0006457; P:protein folding; IEA |
| GO:0005829; C:cytosol; IEA GO:0043234; C:protein complex; IEA |
| GO:0016020; C:membrane; IEA GO:0008654; P:phospholipid biosynthesis; IEA |
| GO:0050660; F:FAD binding; IEA GO:0006118; P:electron transport; IEA |
| GPI10_KLULA GPI mannosyltransferase 3 - Kluyveromyces lactis (Yeast) (Candida sphaerica) |
| HRH3_HUMAN Histamine H3 receptor - Homo sapiens (Human) |
| hypothetical protein PICST_85684 [Pichia stipitis CBS 6054] |
| LMBD2_CAEBR LMBR1 domain-containing protein 2 homolog - Caenorhabditis briggsae |
| MOG2A_XENLA 2-acylglycerol O-acyltransferase 2-A - Xenopus laevis (African clawed frog) |
| MYBA_DICDI Myb-like protein A - Dictyostelium discoideum (Slime mold) |
| NDPA_ENTS8 Nucleoid-associated protein ndpA - Enterobacter sakazakii (strain ATCC BAA-894) |
| NU1M_RANCA NADH-ubiquinone oxidoreductase chain 1 - Rana catesbeiana (Bull frog) |
| NU5M_CAEEL NADH-ubiquinone oxidoreductase chain 5 - Caenorhabditis elegans |
| NUOE2_RHIME NADH-quinone oxidoreductase subunit E 2 - Rhizobium meliloti (Sinorhizobium meliloti) |
| O97341 suberites domuncula (sponge). calmodulin (cam). |
| P08991 strongylocentrotus purpuratus (purple sea urchin). histone h2a variant (fragment). |
| P41824 aplysia californica (california sea hare). y-box factor homolog (apy1). |
| P51544 nordotis madaka (giant abalone). arginine kinase (ec 2.7.3.3) (ak). |
| PDR1_ORYSJ Probable pleiotropic drug resistance protein 1 - Oryza sativa subsp. japonica (Rice) |
| PPAX_BACP2 Pyrophosphatase ppaX - Bacillus pumilus (strain SAFR-032) |
| PRP19_RAT Pre-mRNA-processing factor 19 - Rattus norvegicus (Rat) |
| PUR6_RAT Multifunctional protein ADE2 [Includes: Phosphoribosylaminoimidazole-succinocarboxamide synthase - Rattus norvegicus (Rat) |
| Q25174 hydra attenuata (hydra) (hydra vulgaris). metalloproteinase 1. |
| Q32np0 xenopus laevis (african clawed frog). hypothetical protein. |
| Q4rmm1 tetraodon nigroviridis (green puffer). chromosome 10 scaf15019, whole genome shotgun sequence. |
| Q4sas9 tetraodon nigroviridis (green puffer). chromosome 3 scaf14679, whole genome shotgun sequence. |
| Q4t2f3 tetraodon nigroviridis (green puffer). chromosome undetermined scaf10277, whole genome shotgun sequence. (fragment |
| Q4x807 plasmodium chabaudi. hypothetical protein (fragment). |
| Q502k2 brachydanio rerio (zebrafish) (danio rerio). loc553453 protein (fragment). |
| Q503z0 brachydanio rerio (zebrafish) (danio rerio). hypothetical protein zgc:110003. |
| Q54qx1 dictyostelium discoideum (slime mold). hypothetical protein. |
| Q56fh3 lysiphlebus testaceipes. ribosomal protein p1. |
| Q592t6 lymnaea stagnalis (great pond snail). hypothetical protein (fragment). |
| Q5bx06 schistosoma japonicum (blood fluke). sjchgc04342 protein (fragment). |
| Q5bxc0 schistosoma japonicum (blood fluke). hypothetical protein (fragment). |
| Q5m781 xenopus laevis (african clawed frog). loc496257 protein. |
| Q5snx7 brachydanio rerio (zebrafish) (danio rerio). novel protein similar to vertebrate galactosylceramidase (galc). |
| Q5uqj2 mimivirus. putative ankyrin repeat protein r863. |
| Q5xub9 toxoptera citricida (brown citrus aphid). atp synthase oligomycin sensitivity conferral protein. |
| Q61908 mus musculus (mouse). p8 mtcp-1 protein (mature t-cell proliferation-1 type a) (mtcp-1 type a) (p8mtcp1). |
| Q61hc5 caenorhabditis briggsae. hypothetical protein cbg10794. |
| Q6awn0 drosophila melanogaster (fruit fly). re42209p (fragment). |
| Q6deu6 xenopus tropicalis (western clawed frog) (silurana tropicalis). midline 1 (opitz/bbb syndrome). |
| Q6p9h5 homo sapiens (human). gtpase, imap family member 6, isoform 1 (hypothetical protein dkfzp686a01175). |
| Q6pdk0 mus musculus (mouse). hypothetical protein. |
| Q6pti1 modiolus americanus (american horsemussel). atp synthase beta subunit (fragment). |
| Q6qr01 chiromantes haematocheir. hsp-90. |
| Q70ml9 crassostrea gigas (pacific oyster). hexokinase a-like protein (fragment). |
| Q71bb3 branchiostoma floridae (florida lancelet) (amphioxus). nadph-dependent fmn and fad containing oxidoreductase-like protein. |
| Q7psi5 anopheles gambiae str. pest. ensangp00000002667 (fragment). |
| Q7t2q9 brachydanio rerio (zebrafish) (danio rerio). transketolase (ec 2.2.1.1). |
| Q7zuy5 brachydanio rerio (zebrafish) (danio rerio). ing3 protein. |
| Q802v3 brachydanio rerio (zebrafish) (danio rerio). hypothetical protein zgc:55779. |
| Q86ei2 schistosoma japonicum (blood fluke). clone zzd355 mrna sequence. |
| Q8bsi0 mus musculus (mouse). 12 days embryo male wolffian duct includes surrounding region cdna, riken full-length enriched library, clone:6720468k13 product:weakly similar to hypothetical protein dkfzp434o2413.1 (fragment). |
| Q8ni36 homo sapiens (human). wd-repeat protein 36 (t-cell activation wd repeat protein) (ta-wdrp). |
| Q8wp36 suberites domuncula (sponge). col protein. |
| Q90zq6 oncorhynchus mykiss (rainbow trout) (salmo gairdneri). polyubiquitin. |
| Q9nj23 aequipecten irradians (bay scallop). myosin heavy chain striated muscle specific isoform (fragment). |
| Q9u8w8 tachypleus tridentatus (japanese horseshoe crab). techylectin-5a. |
| Q9v630 drosophila melanogaster (fruit fly). cg8983-pa, isoform a (erp60). |
| Q9v810 drosophila melanogaster (fruit fly). cg4798-pa, isoform a. |
| Q9vlm8 drosophila melanogaster (fruit fly). cg13391-pa, isoform a (cg13391-pb, isoform b). |
| R1AB_FIPV Replicase polyprotein 1ab - Feline coronavirus (strain FIPV WSU-79/1146) (FCoV) |
| RS8_RAT 40S ribosomal protein S8 - Rattus norvegicus (Rat) |
| similar to odorant receptor [Danio rerio] |
| SODF_PLAVI Superoxide dismutase [Fe] - Plasmodium vivax |
| SP2M_BACSU Stage II sporulation protein M - Bacillus subtilis |
| Sugar-specific permease, EIIA 1 domain:PTS system, trehalose-specific IIBC component:PTS system, glucose-like IIB component [Enterococcus faecium DO] |
| TES_XENTR Testin - Xenopus tropicalis (Western clawed frog) (Silurana tropicalis) |
| THYG_MOUSE Thyroglobulin precursor - Mus musculus (Mouse) |
| UBIQ_XENLA Ubiquitin - Xenopus laevis (African clawed frog) |
| V218_FOWPV Putative ankyrin repeat protein FPV218 - Fowlpox virus (FPV) |
| VPS52_SCHPO Vacuolar protein sorting-associated protein 52 - Schizosaccharomyces pombe (Fission yeast) |
| Y101_ARCFU Uncharacterized protein AF_0101 - Archaeoglobus fulgidus |
| YCF2_NYMAL Protein ycf2 - Nymphaea alba (White water-lily) |
| YCX91_PHAAO Uncharacterized protein ORF91 - Phalaenopsis aphrodite subsp. formosana (Moth orchid) |
| YR570_MIMIV Uncharacterized protein R570 - Acanthamoeba polyphaga mimivirus (APMV) |

Table S17. Genes commonly regulated by ATP sulfurylase expression and SOX content in MG mussels

| EST1282; EST276; EST464; EST521; EST60; EST775 |
| --- |
| Aminoadipate aminotransferase [Bos taurus] |
| DPO3B_CHLTR DNA polymerase III subunit beta - Chlamydia trachomatis |
| GO:0004563; F:beta-N-acetylhexosaminidase activity; IEA GO:0005975; P:carbohydrate metabolism; IEA |
| GO:0016020; C:membrane; IEA GO:0008654; P:phospholipid biosynthesis; IEA |
| Q5f1m8 mytilus galloprovincialis (mediterranean mussel). nadh dehydrogenase subunit 4. |
| Q61hc5 caenorhabditis briggsae. hypothetical protein cbg10794. |
| Q7t2q9 brachydanio rerio (zebrafish) (danio rerio). transketolase (ec 2.2.1.1). |
| Q8wp36 suberites domuncula (sponge). col protein. |
| RPOC2_PHYPA DNA-directed RNA polymerase subunit beta'' - Physcomitrella patens (Moss) |
| similar to odorant receptor [Danio rerio] |
| UBIQ_XENLA Ubiquitin - Xenopus laevis (African clawed frog) |

Table S18. Genes with higher expression in MG mussels with low pmoA expression

| EST1015; EST1099; EST114; EST1151; EST408; EST437; EST459; EST670; EST726; EST806 |
| --- |
| 2 sequences no_hits_found |
| GO:0016020; C:membrane; IEA GO:0004872; F:receptor activity; IEA |
| I5P2_HUMAN Type II inositol-1,4,5-trisphosphate 5-phosphatase precursor - Homo sapiens (Human) |
| ITPR_DROME Inositol 1,4,5-trisphosphate receptor - Drosophila melanogaster (Fruit fly) |
| MATK_LOIPR Maturase K - Loiseleuria procumbens (Alpine azalea) (Azalea procumbens) |
| NFYA_RAT Nuclear transcription factor Y subunit alpha - Rattus norvegicus (Rat) |
| NHR89_CAEEL Nuclear hormone receptor family member nhr-89 - Caenorhabditis elegans |
| Q32ns3 xenopus laevis (african clawed frog). hypothetical protein. |
| Q3hri5 mytilus edulis (blue mussel). x-box binding protein 1 (fragment). |
| Q4rel3 tetraodon nigroviridis (green puffer). chromosome 10 scaf15123, whole genome shotgun sequence. |
| Q4w7i9 nasutitermes takasagoensis. hypotheical protein (fragment). |
| Q6ppc9 haliotis rubra. nadh dehydrogenase subunit 5. |
| Q6pti1 modiolus americanus (american horsemussel). atp synthase beta subunit (fragment). |
| Q86ei2 schistosoma japonicum (blood fluke). clone zzd355 mrna sequence. |
| Q8wp36 suberites domuncula (sponge). col protein. |
| Q91423 catfish. phosphoinositide-specific phospholipase c (fragment). |
| YR570_MIMIV Uncharacterized protein R570 - Acanthamoeba polyphaga mimivirus (APMV) |

Table S19. Genes with higher expression in MG mussels with high pmoA expression

| EST1004; EST1010; EST1026; EST1057; EST1123; EST1176; EST1184; EST120; EST1201; EST1205; EST122; EST126; EST1263; EST1264; EST1273; EST1316; EST1318; EST1381; EST1400; EST1414; EST1418; EST1437; EST1448; EST1451; EST1455; EST1459; EST1476; EST1477; EST1479; EST1481; EST1482; EST1497; EST1509; EST1530; EST1547; EST17; EST172; EST186; EST193; EST204; EST212; EST216; EST238; EST240; EST252; EST292; EST349; EST367; EST409; EST427; EST429; EST457; EST46; EST492; EST551; EST554; EST591; EST611; EST62; EST664; EST69; EST820; EST848; EST857; EST858; EST897; EST917; EST92; EST988; EST990 |
| --- |
| 7 sequences no_hits_found |
| 209L2_MACMU CD209 antigen-like protein 2 - Macaca mulatta (Rhesus macaque) |
| ACL6A_MOUSE Actin-like protein 6A - Mus musculus (Mouse) |
| AGAP007259-PA [Anopheles gambiae str. PEST] |
| ALGL_PSEPF Alginate lyase precursor - Pseudomonas fluorescens (strain PfO-1) |
| ASPM_SAIBB Abnormal spindle-like microcephaly-associated protein homolog - Saimiri boliviensis boliviensis (Bolivian squirrel monkey) |
| ATPBM_HEVBR ATP synthase subunit beta, mitochondrial precursor - Hevea brasiliensis (Para rubber tree) |
| ATPG_DROME ATP synthase subunit gamma, mitochondrial precursor - Drosophila melanogaster (Fruit fly) |
| ATPK_DROME Putative ATP synthase subunit f, mitochondrial - Drosophila melanogaster (Fruit fly) |
| CAH1_MONDO Carbonic anhydrase 1 - Monodelphis domestica (Short-tailed gray opossum) |
| CC50A_CHICK Cell cycle control protein 50A - Gallus gallus (Chicken) |
| CO1A2_CHICK Collagen alpha-2(I) chain precursor - Gallus gallus (Chicken) |
| CQ061_BUFBG UPF0451 protein C17orf61 homolog precursor - Bufo bufo gargarizans (Asian toad) |
| DPO5_YEAST DNA polymerase V - Saccharomyces cerevisiae (Baker's yeast) |
| EF1B_RABIT Elongation factor 1-beta - Oryctolagus cuniculus (Rabbit) |
| FLP_YEAST Site-specific recombinase Flp - Saccharomyces cerevisiae (Baker's yeast) |
| GO:0005515; F:protein binding; IPI |
| GO:0005634; C:nucleus; IEA GO:0008270; F:zinc ion binding; IEA |
| GO:0005737; C:cytoplasm; IEA GO:0008152; P:metabolism; IEA |
| GPDA_LACRF Glycerol-3-phosphate dehydrogenase [NAD(P)+] - Lactobacillus reuteri (strain ATCC 23272 / DSM 20016 / F275) |
| IAA3_ARATH Auxin-responsive protein IAA3 - Arabidopsis thaliana (Mouse-ear cress) |
| IF2B_BOVIN Eukaryotic translation initiation factor 2 subunit 2 - Bos taurus (Bovine) |
| Interleukin 8 receptor, alpha [Homo sapiens] |
| KCC1_YEAST Calcium/calmodulin-dependent protein kinase I - Saccharomyces cerevisiae (Baker's yeast) |
| MVP_DISOM Major vault protein - Discopyge ommata (Electric ray) |
| NU1M_DROSU NADH-ubiquinone oxidoreductase chain 1 - Drosophila subobscura (Fruit fly) |
| NU3M_DROYA NADH-ubiquinone oxidoreductase chain 3 - Drosophila yakuba (Fruit fly) |
| NU6M_CERCA NADH-ubiquinone oxidoreductase chain 6 - Ceratitis capitata (Mediterranean fruit fly) |
| O13075 gallus gallus (chicken). nuclear factor nf-kb1. |
| OPDA_ECOLI Oligopeptidase A - Escherichia coli (strain K12) |
| P35003 haliotis rufescens (california red abalone). chymotrypsin-like serine proteinase precursor (ec 3.4.21.-). |
| predicted protein [Nematostella vectensis] |
| PTGES_MACFA Prostaglandin E synthase - Macaca fascicularis (Crab eating macaque) (Cynomolgus monkey) |
| Q27802 tripneustes gratilla (hawaian sea urchin). dynein heavy chain isotype 1b (ec 3.6.1.3). |
| Q30hu9 crassostrea virginica (eastern oyster). serine protease inhibitor 1. |
| Q4gx99 biphyllus lunatus. ribosomal protein l36e. |
| Q4rwx8 tetraodon nigroviridis (green puffer). chromosome 15 scaf14981, whole genome shotgun sequence. (fragment). |
| Q4spx2 tetraodon nigroviridis (green puffer). chromosome 7 scaf14536, whole genome shotgun sequence. (fragment). |
| Q54pk9 dictyostelium discoideum (slime mold). hypothetical protein ddb0216246. |
| Q5bxc0 schistosoma japonicum (blood fluke). hypothetical protein (fragment). |
| Q5d016 brachydanio rerio (zebrafish) (danio rerio). zgc:110742. |
| Q5fw45 xenopus tropicalis (western clawed frog) (silurana tropicalis). mgc107807 protein. |
| Q5m8z2 xenopus tropicalis (western clawed frog) (silurana tropicalis). hypothetical loc496649. |
| Q5ttj1 anopheles gambiae str. pest. ensangp00000028072 (fragment). |
| Q5u275 xenopus laevis (african clawed frog). loc495666 protein. |
| Q5xha7 xenopus laevis (african clawed frog). loc495048 protein. |
| Q658l8 homo sapiens (human). hypothetical protein dkfzp666p126. |
| Q6bcz3 drosophila pseudoobscura (fruit fly). cg4928 (fragment). |
| Q6deg0 brachydanio rerio (zebrafish) (danio rerio). zgc:92367. |
| Q6j0s6 branchiostoma belcheri tsingtaunese. duf614 protein. |
| Q6qm13 lytechinus variegatus (sea urchin). guanine nucleotide-binding protein g(q) alpha subunit (ec 3.6.5.1). |
| Q6usc1 chlamys farreri. cyclophilin a. |
| Q70ml7 crassostrea gigas (pacific oyster). signal sequence receptor beta-like protein (fragment). |
| Q70mp2 crassostrea gigas (pacific oyster). ribosomal protein s3a (fragment). |
| Q70sj8 suberites domuncula (sponge). beta-tubulin. |
| Q7pqt6 anopheles gambiae str. pest. ensangp00000014750 (fragment). |
| Q7yqm3 pongo pygmaeus (orangutan). transcriptional regulator atrx (x-linked helicase ii) (x-linked nuclear protein) (xnp). |
| Q7yzz5 oikopleura dioica. putative alpha-tubulin. |
| Q7zvf8 brachydanio rerio (zebrafish) (danio rerio). diaphorase (nadh) (cytochrome b5 reductase 1). |
| Q86iv5 dictyostelium discoideum (slime mold). similar to dictyostelium discoideum (slime mold). countin (component of the counting factor (cf) complex). |
| Q86lp9 branchiostoma belcheri (amphoxius). hypothetical gaba(a) receptor-associated protein like-2. |
| Q8i6n0 lymnaea stagnalis (great pond snail). camp-responsive element binding protein 2. |
| Q8is80 euprymna scolopes. 60s acidic ribosomal protein (fragment). |
| Q8mv00 tribolium castaneum (red flour beetle). polyubiquitin. |
| Q8r562 mus musculus (mouse). ribonucleoprotein. |
| Q8t697 aplysia californica (california sea hare). beta-thymosin (thymosin-beta). |
| Q8vhs2 mus musculus (mouse). crumbs protein homolog 1 precursor. |
| Q9bpl8 metagonimus yokogawai. cysteine proteinase (fragment). |
| Q9gv70 tegula pfeifferi (pfeiffer's top shell). vitelline coat protein 41. |
| Q9jhg1 mus musculus (mouse). phosphatidylinositol n-acetylglucosaminyltransferase subunit p (ec 2.4.1.198) (phosphatidylinositol-glycan biosynthesis, class p protein) (pig-p) (down syndrome critical region protein 5 homolog). |
| Q9vi23 drosophila melanogaster (fruit fly). cg9727-pa (ld40317p). |
| Q9y156 drosophila melanogaster (fruit fly). cg4778-pa (bcdna.gh02976). |
| Q9z125 mus musculus (mouse). oasis protein. |
| RECR_CAMJD Recombination protein recR - Campylobacter jejuni subsp. doylei (strain ATCC BAA-1458 / RM4099 / 269.97) |
| RIPK5_XENTR Receptor-interacting serine/threonine-protein kinase 5 - Xenopus tropicalis (Western clawed frog) (Silurana tropicalis) |
| RL2_TRIEI 50S ribosomal protein L2 - Trichodesmium erythraeum (strain IMS101) |
| RL21_RAT 60S ribosomal protein L21 - Rattus norvegicus (Rat) |
| RL34_AEDTR 60S ribosomal protein L34 - Aedes triseriatus (Mosquito) (Ochlerotatus triseriatus) |
| RL4_MOUSE 60S ribosomal protein L4 - Mus musculus (Mouse) |
| RL4_RAT 60S ribosomal protein L4 - Rattus norvegicus (Rat) |
| RPB4_MOUSE DNA-directed RNA polymerase II subunit RPB4 - Mus musculus (Mouse) |
| RR3_EPIVI Plastid 30S ribosomal protein S3 - Epifagus virginiana (Beechdrops) |
| RS17_BOVIN 40S ribosomal protein S17 - Bos taurus (Bovine) |
| RS4_DROME 40S ribosomal protein S4 - Drosophila melanogaster (Fruit fly) |
| S22AN_RAT Solute carrier family 22 member 23 - Rattus norvegicus (Rat) |
| SECA2_MYCTU Protein translocase subunit secA 2 - Mycobacterium tuberculosis |
| SPRT_HAEDU Protein sprT - Haemophilus ducreyi |
| SYL_WIGBR Leucyl-tRNA synthetase - Wigglesworthia glossinidia brevipalpis |
| TSN8_BOVIN Tetraspanin-8 - Bos taurus (Bovine) |
| UBC9_ARATH SUMO-conjugating enzyme UBC9 - Arabidopsis thaliana (Mouse-ear cress) |
| VNCS_AEDEV Non-capsid protein NS-1 - Aedes densonucleosis virus (strain GKV 002 002) (Aedes densovirus) |
| Y064_RICTY Uncharacterized protein RT0064 precursor - Rickettsia typhi |
| Y1407_ARCFU Uncharacterized protein AF_1407 - Archaeoglobus fulgidus |
| YNEF_ECOLI Uncharacterized protein yneF - Escherichia coli (strain K12) |
| ZDHC6_DICDI Putative ZDHHC-type palmitoyltransferase 6 - Dictyostelium discoideum (Slime mold) |
| ZEB1_CHICK Zinc finger E-box-binding homeobox 1 - Gallus gallus (Chicken) |

Table S20. Genes commonly regulated by pmoA expression and MOX content in MG mussels

| EST1123; EST1184; EST1201; EST1205; EST126; EST1263; EST1264; EST1273; EST1316; EST1318; EST1400; EST1414; EST1418; EST1437; EST1448; EST1455; EST1459; EST1476; EST1477; EST1479; EST1481; EST1482; EST1497; EST1509; EST216; EST292; EST349; EST367; EST409; EST492; EST551; EST591; EST611; EST664; EST858; EST897 |
| --- |
| AGAP007259-PA [Anopheles gambiae str. PEST] |
| ALGL_PSEPF Alginate lyase precursor - Pseudomonas fluorescens (strain PfO-1) |
| ASPM_SAIBB Abnormal spindle-like microcephaly-associated protein homolog - Saimiri boliviensis boliviensis (Bolivian squirrel monkey) |
| ATPBM_HEVBR ATP synthase subunit beta, mitochondrial precursor - Hevea brasiliensis (Para rubber tree) |
| ATPK_DROME Putative ATP synthase subunit f, mitochondrial - Drosophila melanogaster (Fruit fly) |
| CAH1_MONDO Carbonic anhydrase 1 - Monodelphis domestica (Short-tailed gray opossum) |
| CC50A_CHICK Cell cycle control protein 50A - Gallus gallus (Chicken) |
| CQ061_BUFBG UPF0451 protein C17orf61 homolog precursor - Bufo bufo gargarizans (Asian toad) |
| FLP_YEAST Site-specific recombinase Flp - Saccharomyces cerevisiae (Baker's yeast) |
| GO:0005515; F:protein binding; IPI |
| GO:0005634; C:nucleus; IEA GO:0008270; F:zinc ion binding; IEA |
| IF2B_BOVIN Eukaryotic translation initiation factor 2 subunit 2 - Bos taurus (Bovine) |
| Interleukin 8 receptor, alpha [Homo sapiens] |
| KCC1_YEAST Calcium/calmodulin-dependent protein kinase I - Saccharomyces cerevisiae (Baker's yeast) |
| MVP_DISOM Major vault protein - Discopyge ommata (Electric ray) |
| O13075 gallus gallus (chicken). nuclear factor nf-kb1. |
| OPDA_ECOLI Oligopeptidase A - Escherichia coli (strain K12) |
| P35003 haliotis rufescens (california red abalone). chymotrypsin-like serine proteinase precursor (ec 3.4.21.-). |
| predicted protein [Nematostella vectensis] |
| Q27802 tripneustes gratilla (hawaian sea urchin). dynein heavy chain isotype 1b (ec 3.6.1.3). |
| Q30hu9 crassostrea virginica (eastern oyster). serine protease inhibitor 1. |
| Q4gx99 biphyllus lunatus. ribosomal protein l36e. |
| Q4gxm8 georissus sp. apv-2005. ribosomal protein l6e (fragment). |
| Q4spx2 tetraodon nigroviridis (green puffer). chromosome 7 scaf14536, whole genome shotgun sequence. (fragment). |
| Q5u275 xenopus laevis (african clawed frog). loc495666 protein. |
| Q6deg0 brachydanio rerio (zebrafish) (danio rerio). zgc:92367. |
| Q6j0s6 branchiostoma belcheri tsingtaunese. duf614 protein. |
| Q6qm13 lytechinus variegatus (sea urchin). guanine nucleotide-binding protein g(q) alpha subunit (ec 3.6.5.1). |
| Q6usc1 chlamys farreri. cyclophilin a. |
| Q70mp2 crassostrea gigas (pacific oyster). ribosomal protein s3a (fragment). |
| Q7yzz5 oikopleura dioica. putative alpha-tubulin. |
| Q86iv5 dictyostelium discoideum (slime mold). similar to dictyostelium discoideum (slime mold). countin (component of the counting factor (cf) complex). |
| Q86lp9 branchiostoma belcheri (amphoxius). hypothetical gaba(a) receptor-associated protein like-2. |
| Q8mv00 tribolium castaneum (red flour beetle). polyubiquitin. |
| Q8r562 mus musculus (mouse). ribonucleoprotein. |
| Q8t697 aplysia californica (california sea hare). beta-thymosin (thymosin-beta). |
| Q8vhs2 mus musculus (mouse). crumbs protein homolog 1 precursor. |
| Q9gv70 tegula pfeifferi (pfeiffer's top shell). vitelline coat protein 41. |
| Q9jhg1 mus musculus (mouse). phosphatidylinositol n-acetylglucosaminyltransferase subunit p (ec 2.4.1.198) (phosphatidylinositol-glycan biosynthesis, class p protein) (pig-p) (down syndrome critical region protein 5 homolog). |
| Q9z125 mus musculus (mouse). oasis protein. |
| RL21_RAT 60S ribosomal protein L21 - Rattus norvegicus (Rat) |
| RL34_AEDTR 60S ribosomal protein L34 - Aedes triseriatus (Mosquito) (Ochlerotatus triseriatus) |
| RL4_MOUSE 60S ribosomal protein L4 - Mus musculus (Mouse) |
| RL4_RAT 60S ribosomal protein L4 - Rattus norvegicus (Rat) |
| RPB4_MOUSE DNA-directed RNA polymerase II subunit RPB4 - Mus musculus (Mouse) |
| RR3_EPIVI Plastid 30S ribosomal protein S3 - Epifagus virginiana (Beechdrops) |
| RS17_BOVIN 40S ribosomal protein S17 - Bos taurus (Bovine) |
| SECA2_MYCTU Protein translocase subunit secA 2 - Mycobacterium tuberculosis |
| YNEF_ECOLI Uncharacterized protein yneF - Escherichia coli (strain K12) |
| ZDHC6_DICDI Putative ZDHHC-type palmitoyltransferase 6 - Dictyostelium discoideum (Slime mold) |

Table S21. Genes with higher expression in Rb mussels with low ATP sulfurylase expression

| EST1005; EST1073; EST1090; EST1094; EST1150; EST1169; EST1225; EST1261; EST1406; EST141; EST1438; EST1486; EST1545; EST261; EST323; EST393; EST434; EST443; EST45; EST541; EST645; EST756; EST872; EST879; EST881; EST896; EST912; EST915; EST924; EST945; EST963 |
| --- |
| 11 sequences no_hits_found |
| alpha-2 type I collagen [Gallus gallus] |
| AMPN_FELCA Aminopeptidase N - Felis silvestris catus (Cat) |
| CALM_METSE Calmodulin - Metridium senile (Brown sea anemone) (Frilled sea anemone) |
| CINAL_CYTH3 CinA-like protein - Cytophaga hutchinsonii (strain ATCC 33406 / NCIMB 9469) |
| CLPH_ONCVO Calponin homolog OV9M - Onchocerca volvulus |
| CP4E2_DROME Cytochrome P450 4e2 - Drosophila melanogaster (Fruit fly) |
| CRIM1_CHICK Cysteine-rich motor neuron 1 protein precursor - Gallus gallus (Chicken) |
| cytochrome bd-type quinol oxidase subunit 2 [Exiguobacterium sibiricum 255-15] |
| DTML3_DICDI Dictomallein-3 precursor - Dictyostelium discoideum (Slime mold) |
| DYHC_ONCMY Dynein heavy chain - Oncorhynchus mykiss (Rainbow trout) (Salmo gairdneri) |
| DYL2_RAT Dynein light chain 2, cytoplasmic - Rattus norvegicus (Rat) |
| EFHC2_DANRE EF-hand domain-containing family member C2 - Danio rerio (Zebrafish) (Brachydanio rerio) |
| FOJO_DROME Protein four-jointed [Contains: Protein four-jointed, secreted isoform] - Drosophila melanogaster (Fruit fly) |
| FR1L6_HUMAN Fer-1-like protein 6 - Homo sapiens (Human) |
| GCP_PROMS Probable O-sialoglycoprotein endopeptidase - Prochlorococcus marinus (strain AS9601) |
| GO:0005576; C:extracellular region; IEA GO:0006030; P:chitin metabolism; IEA |
| GO:0016020; C:membrane; IEA GO:0008654; P:phospholipid biosynthesis; IEA |
| GO:0016787; F:hydrolase activity; IEA GO:0008270; F:zinc ion binding; IEA |
| HEM1_PROM9 Glutamyl-tRNA reductase - Prochlorococcus marinus (strain MIT 9312) |
| HSP72_RAT Heat shock-related 70 kDa protein 2 - Rattus norvegicus (Rat) |
| HSP7C_ORYLA Heat shock cognate 71 kDa protein - Oryzias latipes (Medaka fish) (Japanese ricefish) |
| hypothetical protein PY00647 [Plasmodium yoelii yoelii str. 17XNL] |
| LMBD2_CAEBR LMBR1 domain-containing protein 2 homolog - Caenorhabditis briggsae |
| LRP8_HUMAN Low-density lipoprotein receptor-related protein 8 precursor - Homo sapiens (Human) |
| MDTK_KLEP7 Multidrug resistance protein mdtK - Klebsiella pneumoniae subsp. pneumoniae (strain ATCC 700721 / MGH 78578) |
| NALCN_RAT Sodium leak channel non-selective protein - Rattus norvegicus (Rat) |
| NOT1_SCHPO General negative regulator of transcription subunit 1 - Schizosaccharomyces pombe (Fission yeast) |
| NU1M_RANCA NADH-ubiquinone oxidoreductase chain 1 - Rana catesbeiana (Bull frog) |
| NU3M_DROYA NADH-ubiquinone oxidoreductase chain 3 - Drosophila yakuba (Fruit fly) |
| PELP1_MACMU Proline-, glutamic acid- and leucine-rich protein 1 - Macaca mulatta (Rhesus macaque) |
| Q4pmz6 ixodes scapularis (black-legged tick) (deer tick). putative secreted protein. |
| Q5tnp5 anopheles gambiae str. pest. ensangp00000028994 (fragment). |
| Q5xub9 toxoptera citricida (brown citrus aphid). atp synthase oligomycin sensitivity conferral protein. |
| Q6qm13 lytechinus variegatus (sea urchin). guanine nucleotide-binding protein g(q) alpha subunit (ec 3.6.5.1). |
| Q6wn55 branchiostoma belcheri tsingtaunese. adenosylhomocysteinase. |
| Q7t2q9 brachydanio rerio (zebrafish) (danio rerio). transketolase (ec 2.2.1.1). |
| Q8itb7 aequipecten irradians (bay scallop). ribosomal protein s4 (fragment). |
| R1AB_IBVBC Replicase polyprotein 1ab - Avian infectious bronchitis virus (strain Beaudette CK) (IBV) |
| RL21_RAT 60S ribosomal protein L21 - Rattus norvegicus (Rat) |
| RL7_DROME 60S ribosomal protein L7 - Drosophila melanogaster (Fruit fly) |
| RUD3_YEAST GRIP domain-containing protein RUD3 - Saccharomyces cerevisiae (Baker's yeast) |
| S22AN_RAT Solute carrier family 22 member 23 - Rattus norvegicus (Rat) |
| SEMG1_SAGOE Semenogelin-1 precursor - Saguinus oedipus (Cotton-top tamarin) |
| UBC9_ARATH SUMO-conjugating enzyme UBC9 - Arabidopsis thaliana (Mouse-ear cress) |
| UCRI_SAISC Cytochrome b-c1 complex subunit Rieske, mitochondrial precursor - Saimiri sciureus (Common squirrel monkey) |
| unnamed protein product [Tetraodon nigroviridis] |
| Y075_HELAH UPF0078 membrane protein Hac_0075 - Helicobacter acinonychis (strain Sheeba) |
| YD387_YEAST Probable metabolite transport protein YDR387C - Saccharomyces cerevisiae (Baker's yeast) |
| YKAA_CAEEL Uncharacterized amino-acid permease B0303.11 - Caenorhabditis elegans |

Table S22. Genes with higher expression in Rb mussels with high ATP sulfurylase expression

| EST1019; EST1068; EST1086; EST1120; EST1132; EST1155; EST1186; EST1200; EST124; EST1253; EST1334; EST1348; EST142; EST1493; EST1523; EST1550; EST159; EST170; EST179; EST277; EST306; EST370; EST377; EST419; EST430; EST449; EST451; EST484; EST528; EST560; EST564; EST580; EST584; EST606; EST63; EST656; EST670; EST689; EST692; EST698; EST711; EST713; EST723; EST741; EST782; EST791; EST817; EST837; EST904; EST948; EST955; EST98 |
| --- |
| 8 sequences no_hits_found |
| apical endosomal glycoprotein [Strongylocentrotus purpuratus] |
| ATKB_LEPIN Potassium-transporting ATPase B chain - Leptospira interrogans |
| BAZ2B_CHICK Bromodomain adjacent to zinc finger domain protein 2B - Gallus gallus (Chicken) |
| CALM_CANAL Calmodulin - Candida albicans (Yeast) |
| CF165_MACFA Uncharacterized protein C6orf165 homolog - Macaca fascicularis (Crab eating macaque) (Cynomolgus monkey) |
| COX1_BLAGE Cytochrome c oxidase subunit 1 - Blattella germanica (German cockroach) |
| DAXX_RAT Death domain-associated protein 6 - Rattus norvegicus (Rat) |
| DHSA_PONPY Succinate dehydrogenase [ubiquinone] flavoprotein subunit, mitochondrial precursor - Pongo pygmaeus (Bornean orangutan) |
| GGT1_RAT Gamma-glutamyltranspeptidase 1 precursor - Rattus norvegicus (Rat) |
| GO:0005525; F:GTP binding; IEA GO:0006412; P:protein biosynthesis; IEA |
| IF2P_SCHPO Eukaryotic translation initiation factor 5B - Schizosaccharomyces pombe (Fission yeast) |
| LENG8_DANRE Leukocyte receptor cluster member 8 homolog - Danio rerio (Zebrafish) (Brachydanio rerio) |
| MANC_DICDI Alpha-mannosidase C precursor - Dictyostelium discoideum (Slime mold) |
| NADH dehydrogenase subunit 2 [Mytilus galloprovincialis] |
| OXAA_BLOFL Inner membrane protein oxaA - Blochmannia floridanus |
| PHLPP_RAT PH domain leucine-rich repeat protein phosphatase - Rattus norvegicus (Rat) |
| PK1IP_DANRE p21-activated protein kinase-interacting protein 1-like - Danio rerio (Zebrafish) (Brachydanio rerio) |
| Q32ns3 xenopus laevis (african clawed frog). hypothetical protein. |
| Q3zlc7 oreochromis mossambicus (mozambique tilapia) (tilapia mossambica). selenoprotein w2a |
| Q4sas9 tetraodon nigroviridis (green puffer). chromosome 3 scaf14679, whole genome shotgun sequence. |
| Q542v3 m 8 days embryo whole body cdna, riken full-length enriched library, clone:5730499p16 product:splicing factor, arginine/serine-rich 4 (srp75), full insert sequence (activated spleen cdna, riken full- length enriched library, clone:f83011. |
| Q56tz4 rattus norvegicus (rat). macrophage-inducible c-type lectin. |
| Q5f3z5 gallus gallus (chicken). hypothetical protein. |
| Q5r8x0 pongo pygmaeus (orangutan). hypothetical protein dkfzp459c019. |
| Q5tx21 anopheles gambiae str. pest. ensangp00000028140 (fragment). |
| Q5u7a3 gallus gallus (chicken). adenysuccinate lyase. |
| Q5xj47 brachydanio rerio (zebrafish) (danio rerio). h1m protein (fragment). |
| Q6axk1 brachydanio rerio (zebrafish) (danio rerio). zgc:100952. |
| Q6ddx3 xenopus laevis (african clawed frog). mgc81610 protein. |
| Q6f6a1 oryzias latipes (medaka fish) (japanese ricefish). cathepsin l. |
| Q6iah9 homo sapiens (human). loc56901 protein. |
| Q6iwn5 branchiostoma belcheri tsingtaunese. ependymin related protein-1. |
| Q6iwn5 branchiostoma belcheri tsingtaunese. ependymin related protein-1. |
| Q6nwf4 brachydanio rerio (zebrafish) (danio rerio). vacuolar protein sorting protein 25. |
| Q6p267 brachydanio rerio (zebrafish) (danio rerio). zgc:73362. |
| Q6pba6 brachydanio rerio (zebrafish) (danio rerio). plasminogen. |
| Q6ppa1 phytophthora brassicae. kazal-like serine protease inhibitor pbraepi1 (fragment). |
| Q70mt3 crassostrea gigas (pacific oyster). putative ribosomal protein s25 (fragment). |
| Q71bb3 branchiostoma floridae (florida lancelet) (amphioxus). nadph-dependent fmn and fad containing oxidoreductase-like protein. |
| Q76mj8 homo sapiens (human). tdt interacting factor 2. |
| Q8avr7 xenopus laevis (african clawed frog). loc398545 protein (fragment). |
| Q8r179 mus musculus (mouse). kelch repeat and btb domain containing protein 4 (btb and kelch domain containing protein 4). |
| Q91423 catfish. phosphoinositide-specific phospholipase c (fragment). |
| Q9ndl1 patinopecten yessoensis (ezo giant scallop) (yesso scallop). myosin (fragment). |
| ROL3_CAEBR Protein roller-3 precursor - Caenorhabditis briggsae |
| RS3_RAT 40S ribosomal protein S3 - Rattus norvegicus (Rat) |
| SDCB1_RAT Syntenin-1 - Rattus norvegicus (Rat) |
| SPG3_YEAST Stationary phase protein 3 - Saccharomyces cerevisiae (Baker's yeast) |
| SYA_HELPY Alanyl-tRNA synthetase - Helicobacter pylori (Campylobacter pylori) |
| SYI_SULSO Isoleucyl-tRNA synthetase - Sulfolobus solfataricus |
| TYSY_BACFR Thymidylate synthase - Bacteroides fragilis |
| VG2_SPV1R Gene 2 protein - Spiroplasma virus SpV1-R8A2 B (SpV1) (Spiroplasma virus 1) |
| YCF2_EPIVI Protein ycf2 - Epifagus virginiana (Beechdrops) |
| ZO26_XENLA Oocyte zinc finger protein XlCOF26 - Xenopus laevis (African clawed frog) |

Table S23. Genes commonly regultated by ATP sulfurylase expression and SOX content in Rb mussels

| EST323; EST393; EST434; EST45; EST560; EST896 |
| --- |
| apical endosomal glycoprotein [Strongylocentrotus purpuratus] |
| COX1_MYTED Cytochrome c oxidase subunit 1 - Mytilus edulis (Blue mussel) |
| CP4E2_DROME Cytochrome P450 4e2 - Drosophila melanogaster (Fruit fly) |
| cytochrome bd-type quinol oxidase subunit 2 [Exiguobacterium sibiricum 255-15] |
| DYHC_ONCMY Dynein heavy chain - Oncorhynchus mykiss (Rainbow trout) (Salmo gairdneri) |
| HSP7C_ICTPU Heat shock cognate 71 kDa protein - Ictalurus punctatus (Channel catfish) |
| LRP8_HUMAN Low-density lipoprotein receptor-related protein 8 precursor - Homo sapiens (Human) |
| Q6wn55 branchiostoma belcheri tsingtaunese. adenosylhomocysteinase. |
| Q8itb7 aequipecten irradians (bay scallop). ribosomal protein s4 (fragment). |
| YCF2_MORIN Protein ycf2 - Morus indica (Mulberry) |
| YD387_YEAST Probable metabolite transport protein YDR387C - Saccharomyces cerevisiae (Baker's yeast) |

Table S24. Genes with higher expression in Rb mussels with low pmoA expression

| EST1021; EST1062; EST110; EST1115; EST1124; EST1159; EST1161; EST1181; EST1194; EST1197; EST1199; EST1206; EST1222; EST1226; EST1280; EST1316; EST133; EST1331; EST1353; EST1378; EST1384; EST1411; EST1425; EST1491; EST1506; EST1514; EST1524; EST1530; EST162; EST167; EST168; EST201; EST206; EST216; EST225; EST262; EST269; EST271; EST280; EST318; EST320; EST339; EST368; EST38; EST402; EST416; EST511; EST514; EST525; EST534; EST545; EST583; EST589; EST59; EST596; EST61; EST618; EST636; EST644; EST651; EST655; EST669; EST674; EST688; EST693; EST709; EST735; EST736; EST780; EST786; EST790; EST8; EST807; EST826; EST833; EST857; EST892; EST952; EST97; EST995 |
| --- |
| 14 sequences no_hits_found |
| ACE2_YEAST Metallothionein expression activator - Saccharomyces cerevisiae (Baker's yeast) |
| ACL6A_MOUSE Actin-like protein 6A - Mus musculus (Mouse) |
| ACTC_BIOPF Actin, cytoplasmic - Biomphalaria pfeifferi (Bloodfluke planorb) |
| CAC1H_MOUSE Voltage-dependent T-type calcium channel subunit alpha-1H - Mus musculus (Mouse) |
| Caffeoyl-CoA O-methyltransferase [Crocosphaera watsonii WH 8501] |
| CAH1_MONDO Carbonic anhydrase 1 - Monodelphis domestica (Short-tailed gray opossum) |
| calreticulin [Crassostrea gigas] |
| CEL_HUMAN Bile salt-activated lipase precursor - Homo sapiens (Human) |
| CQ061_BUFBG UPF0451 protein C17orf61 homolog precursor - Bufo bufo gargarizans (Asian toad) |
| DAPA_SACD2 Dihydrodipicolinate synthase - Saccharophagus degradans (strain 2-40 / ATCC 43961 / DSM 17024) |
| DISP1_DANRE Protein dispatched homolog 1 - Danio rerio (Zebrafish) (Brachydanio rerio) |
| DMBT1_MOUSE Deleted in malignant brain tumors 1 protein precursor - Mus musculus (Mouse) |
| DPO4_METAC DNA polymerase IV - Methanosarcina acetivorans |
| EF1A_SPOFR Elongation factor 1-alpha - Spodoptera frugiperda (Fall armyworm) |
| EF1B_RABIT Elongation factor 1-beta - Oryctolagus cuniculus (Rabbit) |
| EF2_CAEEL Elongation factor 2 - Caenorhabditis elegans |
| EF2_PONPY Elongation factor 2 - Pongo pygmaeus (Bornean orangutan) |
| EGL1_ARATH Transcription factor EGL1 - Arabidopsis thaliana (Mouse-ear cress) |
| ERP5_YEAST Protein ERP5 precursor - Saccharomyces cerevisiae (Baker's yeast) |
| EX7L_LACS1 Exodeoxyribonuclease 7 large subunit - Lactobacillus salivarius subsp. salivarius (strain UCC118) |
| EXOL_RHIME Succinoglycan biosynthesis protein exoL - Rhizobium meliloti (Sinorhizobium meliloti) |
| FUCO2_HUMAN Plasma alpha-L-fucosidase precursor - Homo sapiens (Human) |
| GO:0005840; C:ribosome; IEA GO:0006412; P:protein biosynthesis; IEA |
| GO:0005840; C:ribosome; IEA GO:0006412; P:protein biosynthesis; IEA |
| GO:0016020; C:membrane; IEA GO:0005529; F:sugar binding; IEA |
| HS12A_HUMAN Heat shock 70 kDa protein 12A - Homo sapiens (Human) |
| hypothetical protein GSPATT00034562001 [Paramecium tetraurelia strain d4-2] |
| hypothetical protein PC301867.00.0 [Plasmodium chabaudi chabaudi] |
| hypothetical protein TA05475 [Theileria annulata strain Ankara] |
| hypothetical protein VvadDRAFT_0340 [Victivallis vadensis ATCC BAA-548] |
| ICB1_HUMAN Induced by contact to basement membrane 1 protein - Homo sapiens (Human) |
| ITPR_DROME Inositol 1,4,5-trisphosphate receptor - Drosophila melanogaster (Fruit fly) |
| KNOB_PLAFN Knob-associated histidine-rich protein precursor - Plasmodium falciparum (isolate NF7/Ghana) |
| LRRC4_MOUSE Leucine-rich repeat-containing 4 protein precursor - Mus musculus (Mouse) |
| MLP2_YEAST Protein MLP2 - Saccharomyces cerevisiae (Baker's yeast) |
| MLTA_BUCAP Membrane-bound lytic murein transglycosylase A homolog - Buchnera aphidicola subsp. Schizaphis graminum |
| MTER1_RAT mTERF domain-containing protein 1, mitochondrial precursor - Rattus norvegicus (Rat) |
| MURD_TRIEI UDP-N-acetylmuramoylalanine--D-glutamate ligase - Trichodesmium erythraeum (strain IMS101) |
| NOG1_RAT Nucleolar GTP-binding protein 1 - Rattus norvegicus (Rat) |
| OUTS_DICD3 Lipoprotein outS precursor - Dickeya dadantii (strain 3937) (Erwinia chrysanthemi (strain 3937)) |
| P35003 haliotis rufescens (california red abalone). chymotrypsin-like serine proteinase precursor (ec 3.4.21.-). |
| PAI2_RAT Plasminogen activator inhibitor 2 type A - Rattus norvegicus (Rat) |
| PLMN_RAT Plasminogen precursor - Rattus norvegicus (Rat) |
| POB_DANRE Protein pob - Danio rerio (Zebrafish) (Brachydanio rerio) |
| PPIF_RAT Peptidyl-prolyl cis-trans isomerase, mitochondrial precursor - Rattus norvegicus (Rat) |
| predicted protein [Nematostella vectensis] |
| PRP5_DEBHA Pre-mRNA-processing ATP-dependent RNA helicase PRP5 - Debaryomyces hansenii (Yeast) (Torulaspora hansenii) |
| Q32n81 xenopus laevis (african clawed frog). hypothetical protein. |
| Q4l223 penaeus monodon (penoeid shrimp). signal transducer and activator of transcription. |
| Q4s6j5 tetraodon nigroviridis (green puffer). chromosome undetermined scaf14725, whole genome shotgun seq. |
| Q54tr4 dictyostelium discoideum (slime mold). hypothetical protein. |
| Q5bxc0 schistosoma japonicum (blood fluke). hypothetical protein (fragment). |
| Q5dbb2 schistosoma japonicum (blood fluke). sjchgc04204 protein. |
| Q5dgp6 schistosoma japonicum (blood fluke). sjchgc05042 protein. |
| Q684l6 lithobius forficatus. translation initiation factor 2 gamma subunit. |
| Q6ddb9 xenopus tropicalis (western clawed frog) (silurana tropicalis). mgc89796 protein. |
| Q6deg0 brachydanio rerio (zebrafish) (danio rerio). zgc:92367. |
| Q6h3x5 pinctada fucata (pearl oyster). qm protein. |
| Q6pc91 brachydanio rerio (zebrafish) (danio rerio). basic transcription factor 3-like 4. |
| Q70sh0 cepaea hortensis. sialic acid binding lectin precursor. |
| Q8r562 mus musculus (mouse). ribonucleoprotein. |
| Q8t772 branchiostoma floridae (florida lancelet) (amphioxus). hypothetical protein. |
| Q8vcx5 m 7 days embryo whole body cdna, riken full-length enriched library, clone:c430019e07 product:similar to calcium binding atopy-related autoantigen 1 homolog (adult male liver tumor cdna, riken full-length enriched library, clone:c730016l. |
| Q956b7 anodonta woodiana. nadh dehydrogenase subunit 2. |
| Q9cpt5 m 10, 11 days embryo whole body cdna, riken full-length enriched library, clone:2810436c08 product:hypothetical protein, full insert sequence (adult male kidney cdna, riken full-length enriched library, clone:0610030b22 product:dna segme. |
| Q9udw1 homo sapiens (human). ubiquinol-cytochrome c reductase complex 7.2 kda protein (ec 1.10.2.2) (cytochrome c1, nonheme 7 kda protein) (complex iii subunit x) (7.2 kda cytochrome c1-associated protein subunit). |
| Q9vvu6 drosophila melanogaster (fruit fly). cg6841-pa (ld04472p). |
| R1A6_SOLDE Putative late blight resistance protein homolog R1A-6 - Solanum demissum (Wild potato) |
| RL16_NEUCR 60S ribosomal protein L16 - Neurospora crassa |
| RL5_ANOGA 60S ribosomal protein L5 - Anopheles gambiae (African malaria mosquito) |
| RPOB_AMBTC DNA-directed RNA polymerase subunit beta - Amborella trichopoda |
| RS11_XENLA 40S ribosomal protein S11 - Xenopus laevis (African clawed frog) |
| RS13_ICTPU 40S ribosomal protein S13 - Ictalurus punctatus (Channel catfish) |
| RS7_DANRE 40S ribosomal protein S7 - Danio rerio (Zebrafish) (Brachydanio rerio) |
| RSHL3_HUMAN Radial spokehead-like protein 3 - Homo sapiens (Human) |
| SAR1B_BOVIN GTP-binding protein SAR1b - Bos taurus (Bovine) |
| SMC2_SCHPO Structural maintenance of chromosomes protein 2 - Schizosaccharomyces pombe (Fission yeast) |
| SYP_BUCAI Prolyl-tRNA synthetase - Buchnera aphidicola subsp. Acyrthosiphon pisum (Acyrthosiphon pisum symbiotic bacterium) |
| TBA_XENLA Tubulin alpha chain - Xenopus laevis (African clawed frog) |
| TEKT3_MOUSE Tektin-3 - Mus musculus (Mouse) |
| VIGLN_MOUSE Vigilin - Mus musculus (Mouse) |
| Y087_BUCAI Uncharacterized protein BU087 - Buchnera aphidicola subsp. Acyrthosiphon pisum (Acyrthosiphon pisum symbiotic bacterium) |
| Y356_MYCGE Uncharacterized protein MG356 - Mycoplasma genitalium |
| Y917_AQUAE Uncharacterized protein aq_917 - Aquifex aeolicus |
| YCF2_MORIN Protein ycf2 - Morus indica (Mulberry) |
| YCF2_PHYPA Protein ycf2 - Physcomitrella patens (Moss) |

Table S25. Genes with higher expression in Rb mussels with high pmoA expression

| EST1000; EST1006; EST1010; EST1056; EST1078; EST1118; EST1160; EST1218; EST1243; EST1248; EST1251; EST1266; EST1270; EST1275; EST1292; EST1312; EST1325; EST1355; EST1363; EST1376; EST1390; EST1424; EST1440; EST1457; EST1460; EST1464; EST1472; EST1474; EST1484; EST1485; EST1496; EST1505; EST1512; EST1539; EST175; EST237; EST268; EST287; EST307; EST328; EST331; EST340; EST343; EST364; EST37; EST4; EST405; EST412; EST459; EST485; EST515; EST529; EST532; EST553; EST569; EST621; EST626; EST632; EST638; EST642; EST659; EST666; EST672; EST680; EST69; EST707; EST716; EST725; EST729; EST731; EST750; EST751; EST784; EST810; EST849; EST891; EST90; EST930; EST99 |
| --- |
| 6 sequences no_hits_found |
| ACTP2_CYMEC Echotoxin-2 precursor - Cymatium echo (Giant triton) (Monoplex echo) |
| APU_THETU Amylopullulanase precursor - Thermoanaerobacter thermosulfurogenes (Clostridium thermosulfurogenes) |
| ARI3A_XENLA AT-rich interactive domain-containing protein 3A - Xenopus laevis (African clawed frog) |
| CAPSD_TTVV9 Capsid protein - Torque teno virus (isolate Japanese macaque/Japan/Mf-TTV9/2000) (TTV) |
| CG4686-PA [Brugia malayi] |
| CO1A2_RAT Collagen alpha-2(I) chain precursor - Rattus norvegicus (Rat) |
| CXB6_HUMAN Gap junction beta-6 protein - Homo sapiens (Human) |
| E2IG5_PONPY E2-induced gene 5 protein homolog - Pongo pygmaeus (Bornean orangutan) |
| GNS_HUMAN N-acetylglucosamine-6-sulfatase precursor - Homo sapiens (Human) |
| GO:0004560; F:alpha-L-fucosidase activity; IEA GO:0005975; P:carbohydrate metabolism; IEA |
| GO:0016020; C:membrane; IEA GO:0007264; P:small GTPase mediated signal transduction; IEA |
| GO:0016020; C:membrane; IEA GO:0008654; P:phospholipid biosynthesis; IEA |
| GPI10_KLULA GPI mannosyltransferase 3 - Kluyveromyces lactis (Yeast) (Candida sphaerica) |
| hypothetical protein OsI_028693 [Oryza sativa (indica cultivar-group)] |
| hypothetical protein PFL1535w [Plasmodium falciparum 3D7] |
| K1B16_MOUSE Kallikrein 1-related peptidase b16 precursor - Mus musculus (Mouse) |
| KHDR3_MOUSE KH domain-containing, RNA-binding, signal transduction-associated protein 3 - Mus musculus (Mouse) |
| LEU3_BUCUN 3-isopropylmalate dehydrogenase - Buchnera aphidicola subsp. Uroleucon sonchi |
| LPE10_CANGA Inner membrane magnesium transporter LPE10, mitochondrial precursor - Candida glabrata (Yeast) (Torulopsis glabrata) |
| MDCB_ACICA Probable 2-(5''-triphosphoribosyl)-3'-dephosphocoenzyme-A synthase - Acinetobacter calcoaceticus |
| NADH dehydrogenase subunit 4L [Cryptopygus antarcticus] |
| NCAN_RAT Neurocan core protein precursor - Rattus norvegicus (Rat) |
| NTP1_AMEPV Nucleoside triphosphatase I - Amsacta moorei entomopoxvirus (AmEPV) |
| NUD1_YEAST Protein NUD1 - Saccharomyces cerevisiae (Baker's yeast) |
| O13075 gallus gallus (chicken). nuclear factor nf-kb1. 3/2004 Length = 1005 |
| O13849 schizosaccharomyces pombe (fission yeast). carboxypeptidase y precursor (ec 3.4.16.5) (cpy). |
| PF2L_PLAFP PPF2L antigen - Plasmodium falciparum (isolate Palo Alto / Uganda) |
| programmed cell death [Culex pipiens quinquefasciatus] |
| Q00277 schistosoma mansoni (blood fluke). glutathione peroxidase (ec 1.11.1.9) (gpx). |
| Q3b7g5 brachydanio rerio (zebrafish) (danio rerio). hypothetical protein (fragment). |
| Q499u1 rattus norvegicus (rat). hypothetical protein rgd1311914_predicted. |
| Q4pm50 ixodes scapularis (black-legged tick) (deer tick). nadh dehydrogenase 1 alpha subcomplex-like. |
| Q4rwx8 tetraodon nigroviridis (green puffer). chromosome 15 scaf14981, whole genome shotgun sequence. (fragment). |
| Q4tga8 tetraodon nigroviridis (green puffer). chromosome undetermined scaf3817, whole genome shotgun sequence (chromosome 1 scaf14573, whole genome shotgun sequence). |
| Q4vbt4 brachydanio rerio (zebrafish) (danio rerio). translation elongation factor 1-gamma. |
| Q54a45 homo sapiens (human). peptidylglycine alpha-amidating monooxygenase. |
| Q54ym7 dictyostelium discoideum (slime mold). hypothetical protein. |
| Q5f0i7 mytilus galloprovincialis (mediterranean mussel). nadh dehydrogenase subunit 5. |
| Q5t8y9 homo sapiens (human). chromosome 10 open reading frame 8. |
| Q5tuc7 anopheles gambiae str. pest. ensangp00000026688. |
| Q5xj54 brachydanio rerio (zebrafish) (danio rerio). thioredoxin-like 2. |
| Q6gmj4 brachydanio rerio (zebrafish) (danio rerio). hypothetical protein zgc:91942. |
| Q6pbw3 brachydanio rerio (zebrafish) (danio rerio). ribosomal protein s14. |
| Q6usc1 chlamys farreri. cyclophilin a. |
| Q76ik7 ciona intestinalis. pol-like protein. |
| Q7zyt3 xenopus laevis (african clawed frog). similar to ribosomal protein s3. |
| Q8c0s0 mus musculus (mouse). adult male testis cdna, riken full-length enriched library, clone:4932411n23 product:weakly similar to cyclin-d binding myb-like protein. |
| Q8iu34 crassostrea gigas (pacific oyster). vitellogenin. |
| Q91733 xenopus laevis (african clawed frog). elongation factor-1 delta (eef1d protein). |
| Q9bnw3 chaetopleura apiculata (common eastern chiton). elongation factor-2 (fragment). |
| Q9p5z0 neurospora crassa. related to cofilin. |
| Q9vpi4 drosophila melanogaster (fruit fly). cg31973-pa, isoform a. |
| Q9vq62 drosophila melanogaster (fruit fly). hypothetical protein npc2 precursor (niemann pick type c2 protein homolog). |
| Q9y092 drosophila melanogaster (fruit fly). cg8604-pa (amphiphysin) (ld19810p). |
| R1AB_BEV Replicase polyprotein 1ab - Berne virus (BEV) |
| RPOA_EAVBU Replicase polyprotein 1ab - Equine arteritis virus (strain Bucyrus) (EAV) |
| RS6_APLCA 40S ribosomal protein S6 - Aplysia californica (California sea hare) |
| SMRD2_HUMAN SWI/SNF-related matrix-associated actin-dependent regulator of chromatin subfamily D member 2 - Homo sapiens (Human) |
| SQSTM_RAT Sequestosome-1 - Rattus norvegicus (Rat) |
| SYL_FERNB Leucyl-tRNA synthetase - Fervidobacterium nodosum (strain ATCC 35602 / DSM 5306 / Rt17-B1) |
| SYL_WIGBR Leucyl-tRNA synthetase - Wigglesworthia glossinidia brevipalpis |
| TEKT3_HUMAN Tektin-3 - Homo sapiens (Human) |
| TETX_CLOTE Tetanus toxin precursor - Clostridium tetani |
| THN22_ARATH Thionin-2.2 precursor [Contains: Thionin-2.2; Acidic protein] - Arabidopsis thaliana (Mouse-ear cress) |
| TM104_PONPY Transmembrane protein 104 - Pongo pygmaeus (Bornean orangutan) |
| TR112_CAEEL TRM112-like protein - Caenorhabditis elegans |
| TSN8_BOVIN Tetraspanin-8 - Bos taurus (Bovine) |
| UNC5D_MOUSE Netrin receptor UNC5D precursor - Mus musculus (Mouse) |
| ZFAND_DICDI AN1-type zinc finger protein - Dictyostelium discoideum (Slime mold) |

Table S26. Genes commonly regulated by pmoA expression and MOX content in Rb mussels

| EST1006; EST1118; EST1159; EST1160; EST1161; EST1181; EST1194; EST1218; EST1222; EST1248; EST1331; EST1363; EST1390; EST1411; EST1484; EST1491; EST1505; EST1506; EST1514; EST1524; EST1539; EST262; EST280; EST318; EST339; EST37; EST4; EST416; EST583; EST589; EST596; EST644; EST655; EST659; EST669; EST688; EST693; EST709; EST735; EST736; EST786; EST790; EST849; EST97; EST995 |
| --- |
| ACTP2_CYMEC Echotoxin-2 precursor - Cymatium echo (Giant triton) (Monoplex echo) |
| CO1A2_CHICK Collagen alpha-2(I) chain precursor - Gallus gallus (Chicken) |
| CQ061_BUFBG UPF0451 protein C17orf61 homolog precursor - Bufo bufo gargarizans (Asian toad) |
| DAPA_SACD2 Dihydrodipicolinate synthase - Saccharophagus degradans (strain 2-40 / ATCC 43961 / DSM 17024) |
| DPO4_METAC DNA polymerase IV - Methanosarcina acetivorans |
| EF1A_SPOFR Elongation factor 1-alpha - Spodoptera frugiperda (Fall armyworm) |
| EX7L_LACS1 Exodeoxyribonuclease 7 large subunit - Lactobacillus salivarius subsp. salivarius (strain UCC118) |
| hypothetical protein GSPATT00034562001 [Paramecium tetraurelia strain d4-2] |
| hypothetical protein OsI_028693 [Oryza sativa (indica cultivar-group)] |
| hypothetical protein PFL1535w [Plasmodium falciparum 3D7] |
| hypothetical protein TA05475 [Theileria annulata strain Ankara] |
| ICB1_HUMAN Induced by contact to basement membrane 1 protein - Homo sapiens (Human) |
| MURD_TRIEI UDP-N-acetylmuramoylalanine--D-glutamate ligase - Trichodesmium erythraeum (strain IMS101) |
| NCAN_RAT Neurocan core protein precursor - Rattus norvegicus (Rat) |
| NOG1_RAT Nucleolar GTP-binding protein 1 - Rattus norvegicus (Rat) |
| NUD1_YEAST Protein NUD1 - Saccharomyces cerevisiae (Baker's yeast) |
| OUTS_DICD3 Lipoprotein outS precursor - Dickeya dadantii (strain 3937) (Erwinia chrysanthemi (strain 3937) |
| PF2L_PLAFP PPF2L antigen - Plasmodium falciparum (isolate Palo Alto / Uganda) |
| PLMN_RAT Plasminogen precursor - Rattus norvegicus (Rat) |
| predicted protein [Nematostella vectensis] |
| programmed cell death [Culex pipiens quinquefasciatus] |
| Q32n81 xenopus laevis (african clawed frog). hypothetical protein. |
| Q4l223 penaeus monodon (penoeid shrimp). signal transducer and activator of transcription. |
| Q4pm50 ixodes scapularis (black-legged tick) (deer tick). nadh dehydrogenase 1 alpha subcomplex-like. |
| Q54tr4 dictyostelium discoideum (slime mold). hypothetical protein. |
| Q5dbb2 schistosoma japonicum (blood fluke). sjchgc04204 protein. |
| Q5dgp6 schistosoma japonicum (blood fluke). sjchgc05042 protein. |
| Q684l6 lithobius forficatus. translation initiation factor 2 gamma subunit. |
| Q6pc91 brachydanio rerio (zebrafish) (danio rerio). basic transcription factor 3-like 4. |
| Q70sh0 cepaea hortensis. sialic acid binding lectin precursor. |
| Q8iu34 crassostrea gigas (pacific oyster). vitellogenin. |
| Q8r562 mus musculus (mouse). ribonucleoprotein. |
| Q9bnw3 chaetopleura apiculata (common eastern chiton). elongation factor-2 (fragment). |
| Q9cpt5 m 10, 11 days embryo whole body cdna, riken full-length enriched library, clone:2810436c08 product:hypothetical protein, full insert sequence (adult male kidney cdna, riken full-length enriched library, clone:0610030b22 product:dna segme. |
| Q9vvu6 drosophila melanogaster (fruit fly). cg6841-pa (ld04472p). |
| RL16_NEUCR 60S ribosomal protein L16 - Neurospora crassa |
| RPOB_AMBTC DNA-directed RNA polymerase subunit beta - Amborella trichopoda |
| RS6_APLCA 40S ribosomal protein S6 - Aplysia californica (California sea hare) |
| RSHL3_HUMAN Radial spokehead-like protein 3 - Homo sapiens (Human) |
| SMC2_SCHPO Structural maintenance of chromosomes protein 2 - Schizosaccharomyces pombe (Fission yeast) |
| SMRD2_HUMAN SWI/SNF-related matrix-associated actin-dependent regulator of chromatin subfamily D member 2 - Homo sapiens (Human) |
| SQSTM_RAT Sequestosome-1 - Rattus norvegicus (Rat) |
| SYL_FERNB Leucyl-tRNA synthetase - Fervidobacterium nodosum (strain ATCC 35602/DSM 5306/ Rt17-B1) |
| TBA3_RAT Tubulin alpha-3 chain - Rattus norvegicus (Rat) |
| TEKT3_MOUSE Tektin-3 - Mus musculus (Mouse) |
| TETX_CLOTE Tetanus toxin precursor - Clostridium tetani |
| TM104_PONPY Transmembrane protein 104 - Pongo pygmaeus (Bornean orangutan) |
| TR112_CAEEL TRM112-like protein - Caenorhabditis elegans |
| VIGLN_MOUSE Vigilin - Mus musculus (Mouse) |
| Y917_AQUAE Uncharacterized protein aq_917 - Aquifex aeolicus |
| YCF2_PHYPA Protein ycf2 - Physcomitrella patens (Moss) |
